# Supervised exercise therapy compared with no exercise therapy to reverse debilitating effects of androgen deprivation therapy in patients with prostate cancer: a systematic review and meta-analysis

**DOI:** 10.1038/s41391-021-00450-0

**Published:** 2021-09-06

**Authors:** Anja Ussing, Marie-Louise Kirkegaard Mikkelsen, Brigitta Rasmussen Villumsen, Johnny Wejlgaard, Pernille Envold Bistrup, Kirsten Birkefoss, Thomas Bandholm

**Affiliations:** 1 Danish Health Authority, Copenhagen, Denmark; 2 The Parker Institute, Copenhagen University Hospital, Bispebjerg and Frederiksberg, Copenhagen, Denmark; 3 Department of Urology, NIDO | danmark, Regional Hospital Gødstrup, Holstebro, Denmark; 4 Copenhagen Centre for Cancer and Health, Copenhagen, Denmark; 5 Psychological aspects of Cancer, Danish Cancer Society Research Center, Danish Cancer Society, Copenhagen, Denmark; 6 Department of Physiotherapy and Occupational Therapy, Copenhagen University Hospital, Hvidovre, Denmark; 7 Department of Clinical Research, Copenhagen University Hospital, Hvidovre, Denmark; 8 Physical Medicine and Rehabilitation Research-Copenhagen, Copenhagen University Hospital, Hvidovre, Denmark

**Keywords:** Prostate cancer, Cancer therapy

## Abstract

**Background:**

Androgen deprivation therapy (ADT) in patients with prostate cancer can have several debilitating side effects. Supervised exercise is recommended to ameliorate these negative effects.

**Objective:**

To systematically evaluate the effect of supervised exercise therapy compared to no exercise therapy in patients with prostate cancer undergoing ADT, primarily according to the patient critical outcomes, ‘disease-specific quality of life’ and ‘walking performance’ measured at end of treatment.

**Methods:**

We searched PubMed/Medline, Embase, Cochrane Library, Cinahl and Pedro, to identify randomised controlled trials (RCTs), which investigated the effect of supervised exercise therapy compared to no exercise therapy in patients with prostate cancer receiving ADT, last search: June 2021. Two independent reviewers extracted data, and assessed risk of bias using Cochrane Risk of Bias Tool and evaluated the certainty of evidence using the GRADE-method.

**Results:**

Eigthteen RCTs (*n* = 1477) comprised patients with prostate cancer stages T1-T4 were included in the meta-analyses. Compared to no exercise therapy, supervised exercise therapy showed clinically relevant improvements in ‘disease-specific quality of life’ and ‘walking performance’. The standardised mean differences were 0.43 (95% confidence interval (CI): 0.29, 0.58) and −0.41 (95% CI: −0.60, −0.22), respectively. The overall certainty of evidence was moderate due to serious risk of bias.

**Conclusions:**

Evidence of moderate quality shows that supervised exercise therapy probably is superior to no exercise therapy in improving ‘disease-specific quality of life’ and ‘walking performance’ in patients with prostate cancer undergoing ADT. The results apply to all patients receiving androgen deprivation therapy regardless of cancer stage. The results support a strong recommendation for supervised exercise therapy for managing side effects in this population.

**Protocol registration:**

NKR-38-Focused-questions-PICOs-for-updating1.ashx (sst.dk)

## Introduction

Prostate cancer is the second most common cancer type among men worldwide and the fifth most frequent cause of cancer deaths in men [1]. Prostate tumour growth is dependent on stimulation from the male hormone testosterone [2]. Androgen deprivation therapy (ADT) significantly lowers the levels of testosterone or inactivate the function of the hormone. This lead to slowing tumour growth or shrinking the tumour size [3]. Therefore, ADT is foundational in treatment of metastatic prostate cancer, and used as an important adjuvant therapy in locally advanced prostate cancer [3–6]. Up to half of all patients with prostate cancer will undergo ADT at some time point during their treatment course [7]. The benefits of ADT in delaying prostate cancer progression, relieving symptoms and prolonging survival for patients with advanced disease is widely documented [4–6]. Unfortunately, ADT can have several detrimental side effects including; increases in fat mass, low bone mineral density, loss of muscle mass and reduced muscle strength [8–10]. These side effects can lead to significant reductions in physical performance of everyday activities [8, 11] and reduced quality of life [12], and they can increase the risk of cardiovascular diseases, diabetes, fractures [5, 8, 9] and depression [8, 13, 14]. The management of these considerable negative effects is an essential part of the supportive cancer care for men receiving ADT [9, 12, 15].

Randomized controlled trials (RCT) have shown that supervised exercise can ameliorate many of the debilitating effects of ADT [16–22], and supervised exercise is recommended as a strategy to manage these side effects [9, 15]. Some systematic reviews have demonstrated beneficial effects of exercise therapy in reversing side effects of ADT in meta-analyses [23–26], but several RCTs have been published since these reviews were conducted [20, 21, 27–31].

None of the previous reviews have used the GRADE-approach (Grades of Recommendation, Assessment, Development and Evaluation). The strength of the GRADE-method includes pre-specification of the inclusion criteria and pre-specified outcomes, judged as critical or important to patients. This process ideally involves asking patients [32, 33].

The aim of this review was to systematically evaluate the effect of supervised exercise therapy compared with no exercise therapy in patients with prostate cancer undergoing ADT using GRADE. The effect was assessed using the patient critical outcomes ‘disease-specific quality of life’ and ‘physical performance’ measured by walking performance at end of treatment.

## Methods

This systematic review and meta-analysis was conducted according to the guidelines of the Cochrane Collaboration [34] and based on the GRADE-approach [35]. The reporting adheres to the Preferred Reporting Items for Systematic Reviews and Meta-analyses (PRISMA) recommendations [36]. The work was conducted as a part of updating a national clinical guideline on rehabilitation of patients with prostate cancer published by the Danish Health Authorities in 2021 [37]. As such, the protocol was pre-specified including specification of detailed inclusion and exclusion criteria regarding populations, interventions, comparators and outcomes. The protocol was approved by the management of the Danish Health Authority before the literature search was conducted and is publicly available at NKR-38-Focused-questions-PICOs-for-updating1.ashx (sst.dk).

### Data Sources and Search Strategy

First, to identify systematic reviews with relevant RCTs to be included in the synthesis, we performed a search for systematic reviews on 18th January 2016 including records published from 2005-2016. In this step, we included a systematic review by Bourke et al. [16]. Next, a systematic search for primary trials was conducted on 24th February 2016. The search was limited to the last search in the identified review (January 2015). This search was updated on 16th June 2021. Searches were conducted in PubMed/MEDLINE, EMBASE, Cochrane Library, Cinahl and Pedro. No restrictions regarding publication status were applied. Language was limited to English, Danish, Swedish and Norwegian. See the full search protocols in Supplementary appendix.

### Trial selection

We used Covidence systematic review software (Veritas Health Innovation, Melbourne, Australia) for screening, data extraction and risk of bias assessments. One reviewer screened all titles and abstracts for eligibility (AU), and two reviewers independently assessed records selected for full-text review (AU and BV, JV or MLK). Disagreements were resolved through discussions. If necessary, a third reviewer (BV or JV) was consulted to reach consensus. Reference lists of the included trials and relevant identified systematic reviews were hand searched for more relevant trials.

Pre-specified eligibility criteria were based on the Population, Intervention, Comparison, Outcome, and Time (PICOT) framework [33, 38]. We included RCTs that investigated the effect of supervised exercise therapy compared to no exercise therapy in patients with prostate cancer receiving ADT. Both published and unpublished trials could be included as long as results were available in an abstract or at a website. The PICOT eligibility criteria for inclusion and exclusion of trials were as follows:

### Population

Adult patients diagnosed with prostate cancer currently receiving ADT. All types of ADT could be included. Trials in which more than 60% of the population received ADT could also be included. When such trials were included in an analysis, we performed a pre-specified sensitivity analysis with exclusion of these trials. Trials with a subgroup of patients on ADT (<60%) were included as long as data were provided separately for the ADT-population.

### Intervention

Supervised exercise therapy was defined as: a regimen of physical exercises that was instructed, supervised, and monitored by a health care professional. We included trials with physical exercises involving the whole body at a moderate to high intensity e.g. resistance training involving the upper and lower extremity with an intensity of minimum 60% of one repetition maximum (RM) and/or aerobic (cardiovascular) exercise at a minimum of 60% of the estimated maximum heart rate. Supervision had to be given at least twice per week and length of interventions minimum two months.

### Comparator

We defined ‘no exercise therapy’ as: no treatment, usual care not including physical exercise therapy, waiting list, and sham training e.g. stretching or relaxation training. Home-based exercise after initial instruction was not included as comparator.

### Outcome

According to GRADE, outcomes were predefined as critical or important to patients [33, 38]. Patient representatives from the Danish Association of Prostate Cancer contributed to the ratings of outcomes.

Critical (primary) outcomes were ‘disease-specific quality of life’ and ‘physical performance measured by walking performance’. The preferred measure for disease-specific quality of life was The Functional Assessment of Cancer Therapy–Prostate (FACT-P) (range 0–156, Minimum Clinically Important Difference (MCID) 6–10 points [39]. For ‘walking performance’ we preferred 400 m walking test when available (MCID: 20–30 s) [40].

Important (not critical, secondary) outcomes included ‘health related quality of life’, e.g. SF-26 or EQ-5D, ‘physical performance measured by sit to stand performance, ‘muscle strength’, ‘VO2 peak’, ‘prevalence of depression, cardiovascular diseases and diabetes’, ‘fractures’, ‘exercise related injuries’ and ‘dropouts for all causes’.

### Time point of interest

The primary time point of interest was end of treatment for all outcomes except for the outcomes ‘fractures’, ‘prevalence of depression’, ‘prevalence of cardiovascular diseases’ and ‘prevalence of diabetes’ for which the time point of interest was longest follow-up.

### Data extraction and quality assessments

Two authors (AU and MLK) independently extracted data using a predefined extraction template in Covidence including information of trial design, trial population, baseline characteristics, interventions, comparators and outcome measures. Discrepancies were resolved through discussion.

We evaluated the internal validity of the included systematic review using the AMSTAR tool [41] and assessed risk of bias using Cochrane Risk of Bias tool [42], which evaluates random sequence generation, allocation concealment, blinding of personnel, patients and outcome assessors, incomplete outcome data, selective outcome reporting and other sources of bias. Two independent reviewers (AU and MLK) performed all quality assessments. Consensus was reached through discussions.

### Certainty of evidence

The certainty of evidence per outcome across trials was assessed using GRADE [43]. According to GRADE, evidence from RCTs starts at “high certainty” and can be downgraded to “moderate”, “low”, or “very low” certainty based on limitations in trial design (risk of bias), indirectness, imprecision, inconsistency, and publication bias. The overall certainty of evidence was determined by the lowest certainty level for the critical outcomes [43]. All assessments were decided by consensus in the guideline panel.

### Statistical analyses

Meta-analyses were performed as random effects model using Review Manager 5.3 (The Nordic Cochrane Centre, The Cochrane Collaboration, Copenhagen, Denmark) as variation between studies were anticipated. Missing values for standard deviations were calculated from the available data when possible. Continuous outcomes were reported as mean differences (MD) when data were reported at the same measurement scale, otherwise standardised mean differences (SMD) were calculated. To support interpretation, SMD estimates for critical outcomes were transformed to MD estimates, using the method described by Thorlund et al. [44]. Dichotomous outcomes were reported as Risk Ratios. For all estimates, 95% confidence intervals (CIs) were provided. Absolute effect estimates per 1000 individuals and corresponding CIs were calculated based on the presumed risk in the control group and the estimated risk ratio. When at least one trial reported no events in one group for a dichotomous outcome, the absolute effect was based on a risk difference analysis. Heterogeneity was assessed by visual inspection of the forest plot, and by interpreting the I² statistic and Chi²-test. We generated funnel plots to judge publication bias when ten or more trials were included in an analysis.

To explore potential heterogeneity, the following subgroup analyses were pre-specified: group vs individual training, timing of exercise after starting ADT (>1 month vs <1 month) and modalities e.g. football (European soccer), resistance or aerobic exercise. In addition, pre-planned sensitivity analyses excluding trials where less than 90% of the patients received ADT were conducted.

## Results

### Selection of trials

In the search for systematic reviews we identified 732 records. We excluded 644 by screening of title and abstracts, and 68 records were assessed for inclusion by full-text review. One systematic review was included [16]. From this review, we included seven RCTs after full text review. In the search for primary studies (February 2016) we identified 149 records of which 17 were assessed for inclusion by full-text review and one RCT was included. The update of this search (June 2021) identified another 871 records, of which 109 were assessed by full-text review and 10 RCTs were included. In total 18 RCTs (25 publications) were included [17–22, 27–31, 45–58]. See PRISMA flow charts for the trial selection process in Supplementary Figure S1, S2. A complete list of excluded trials assessed in full-text with reasons for exclusion is given in Supplementary Table S1.

### Trial characteristics

The 18 eligible trials included 1,477 men with prostate cancer for our comparisons of interest. For 16 of the 18 included trials a protocol was available [18–22, 27–31, 45–50, 52–56], but in two trials the protocol was registered retrospectively [18, 49, 50, 52, 54]. In the majority of the trials, all participants (100%) received ADT [18–22, 28, 30, 31, 45–56] or data were reported separately for the ADT population [27]. In one trial 95% received ADT [29] and in another trial 61% received ADT [17]. The included trials comprised patients with cancer stage T1-T4. Nine trials included patients with stage T1-T4 cancer [17, 21, 22, 27, 31, 47, 50–54, 56], five trials included stage T3-T4 [18, 19, 29, 30, 45], and in four trials cancer stages were unclear [20, 28, 46, 48].

Four trials included men, who had started ADT within the last month [21, 22, 30, 46, 48]. In the remaining trials, the participants had been on ADT between two month and three years. One trial had no information on ADT duration [17]. The interventions comprised supervised exercise therapy with moderate to high intensity between 60–90% of 1 RM (repetition maximum) for resistance exercise and between 55–85% of the estimated maximum heart rate for aerobic exercise. The majority of the exercise programs were progressive in nature [17–22, 27–31, 45–48, 50, 52–58].

The included interventions consisted of a combination of resistance and aerobic exercise [18, 20–22, 29–31, 45–50, 52, 54], resistance or aerobic exercise [17], solely resistance exercise [19, 28, 51, 55, 56] and football training [27, 53]. The duration of the interventions was 12 weeks [20, 28–30, 45–47], 16 weeks [19, 48, 51, 53], 6 months [17, 21, 22, 27, 50, 52, 54] and 12 months [18, 31, 49, 55, 56] for our comparisons of interests, respectively. Trial characteristics are presented in Table 1.

**Table 1:** Characteristics of included trials.

Author, year, country, trial registration	Participants *n*, mean age, (SD), *n* (%) on ADT, Time on ADT mean (SD)	Design and funding	Intervention (*s*)	Comparison	Duration	Outcomes of interest
Bjerre, 2009, Denmark [26] Trial registration: Clinicaltrials.gov identifier: NCT02430792	*n* = 97 on ADT (214 in total) We only extracted data for the ADT population, except for the outcome dropouts. Tumour stage: T1–T4 Football group (*n* = 46) Age, years: 67.8 (6.2) Number on ADT: 46 (42%) Time on ADT in days, median (IQR): 512.5 days (208-88) Control group (*n* = 41) Age, years: 69.0 (6.2) Number on ADT: 41(39%) Time on ADT in days, median (IQR): 580 days (235–1089)	RCT multicenter (5 sites), parallel group, two arms. Funding: TrygFonden and Danish Cancer Society	Recreational football 20 min warm-up, 20 min practicing dribbling, passing and shooting and 20 min of 5–7-a-side football. One hour twice weekly for 6 months.	Usual care A phone-based counselling session (5–15 min) as part of the information on group allocation, as well as information via email on the current physical activity guidelines.	6 months	Disease-specific quality of life: Fact-P Health related quality of life: SF-36, physical and mental component Fractures: Number of patients with a fracture Exercise related injuries: Number of injuries, only reported for the football group Dropouts: Number of participants
Bourke, 2014, UK [41] Trial registration: ISRCTN.com identifier: ISRCTN88605738	*n* = 100, Tumour stage: T3–T4 Supervised exercise group (*n* = 50) Age, years: 71 (6) Number on ADT: 50 (100) Time on ADT in months: 33 (33) Control group (*n* = 50) Age, years: 71 (8) Number on ADT: 50 (100) Time on ADT in months: 30 (30)	RCT, single center, parallel group, two arms. Funding: None	Supervised aerobic and resistance exercise^a^ in groups + a dietary advice intervention and behaviour change support. Aerobic exercise: 30 min at 55–85% of the age predicted maximum heart rate. Resistance exercise: Starting with 2–4 sets of 8–12 repetitions at 60% of 1 RM. Twice a week from weeks 1–6, and once per week from weeks 7–12. Home-based training ones a week for the first 6 weeks and twice per week during weeks 7–12.	Usual care Followed up in the urology clinic and seen by an oncology nurse specialist and urologist.	12 weeks	Disease-specific quality of life: Fact-P Fractures: Number of patients with a fracture. Exercise related injuries: Number of participants with skeletal related adverse events. Dropouts: Number of participants
Cormie, 2015, Australia [42] Trial registration: Anztct.org.au identifier: ACTRN 12610000691044	*n* = 63, Tumour stage: Not clear Supervised exercise group (*n* = 32) Age, years: 69.9 (5.5) Number on ADT: 32 (100) Time on ADT in days: 6.2 (1.16) Control group (*n* = 31) Age, years: 67.1 (7.5) Number on ADT: 31 (100) Time on ADT in days: 5.6 (2.0)	RCT, multicenter (six sites), parallel group, two arms. Funding: Abbvie Pty Ltd	Supervised aerobic and resistance exercises^a^ in groups, including standard warm-up and cool-down periods. Aerobic exercise: 20–30 min at 70–85% of estimated maximum heart rate Resistance exercise: 1–4 sets of 6–12 repetitions at 60–85% of 1 RM. 60 min twice weekly. Encouraged to home-based aerobic of 150 min of moderate intensity aerobic exercise each week.	Usual care Maintained standard medical care for the treatment of prostate cancer and were instructed to maintain their customary activity and dietary patterns throughout the intervention period. Participants in the control group were offered the exercise program after the completion of the intervention period.	3 months	Health related quality of life: SF-36, physical and mental component Physical function: Repeated chair raise, 5 repetitions. Muscle strength: Leg press, 1 RM Vo2 peak: ml/Kg/min Depressive symptoms: BSI 18 Fractures: Number of patients with a fracture Exercise related injuries: Numbers Dropouts: Number of participants
Dawson, 2018, USA [27] Trial registration: Clinicaltrials.gov identifier: NCT01909440	*n* = 37, Tumour stage: Not clear Exercise groups combinded (*n* = 16) Age, years: 68.6 (8.4) Number on ADT: 16 (100) Time on ADT in months: 14.6 (15.4) Control groups combined (*n* = 21) Age, years: 66.3 (9.0) Number on ADT: 19 (100) Time on ADT in months: 12.7 811.6)	RCT, single center, parallel group, four arms. Funding: National Strength and Conditioning Association and the California State University.	Group 1 and 2: Supervised resistance exercise^a^ in groups, with (group 1) and without (group 2) protein supplementation (50 g a day of whey protein isolate). 5 min warm-up Resistance exercise: 3 sets of 8–15 repetitions at 60–83% of 1 RM. 5 min stretching exercises 50 min three days a week.	Group 3 and 4: Home-based flexibility program with (group 3) and without (group 4) protein supplementation (50 g a day of whey protein isolate). Each stretching session lasted 5 min and matched the stretches performed by the exercise groups 5 min 3 times per week for 12 weeks.	12 weeks The results were given respectively for the two exercise groups combined and the two control groups combined	Disease-specific quality of life: Fact-P Physical function: 400 m walking test. Muscle strength: Leg press Depressive symptoms: CES-D Fractures: Number of patients with a fracture Exercise related injuries: Numbers Dropouts: Number of participants
Focth, 2018, USA [19] Trial registration: Clinicaltrials.gov identifier: NCT02050906	*n* = 32, Tumour stage: Not clear Supervised exercise group (*n* = 16) Age, years: 69.4 (9.0) Number on ADT: 16 (100) Time on ADT in months: 32.18 (27.28) Control group (*n* = 16) Age, years: 64.5 (8.6) Number on ADT:16 (100) Time on ADT in months:15.31 (19.39)	RCT, single center, parallel group, two arms. Funding: National Cancer Institute, USA	Supervised aerobic and resistance exercises^a^ in groups + a dietary intervention. Aerobic exercise: 10–20 min of light to moderate hard intensity. Resistance exercise: 3 sets of 8–12 repetitions (8–12 RM). One hour twice per week. Encouraged to independent home-based exercise and physical activity up to150 min of per week.	Usual care Usual prostate cancer treatment and standard disease management education, as well as additional educational literature describing the American Institute of Cancer Research dietary and physical activity guidelines and education.	12 weeks	Physical function: 400 m walking test. Muscle strength: Leg extension. Fractures: Number of patients with a fracture Exercise related injuries: Numbers Dropouts: Number of participants
Galvao, 2010, Australia [43] Trial registration: Anztct.org.au identifier: ACTRN 12607000263493	*n* = 57, Tumour stage: T1–T4 Supervised exercise group (*n* = 29) Age, years: 69.5 (7.3) Number on ADT: 29 (100) Time on ADT in months: 18.2 (38.5) Control group (*n* = 28) Age, years: 70.1 (7.3) Number on ADT: 28 (100) Time on ADT in months: 10.1 (26.8)	RCT, single center, parallel group, two arms. Funding: Cancer Council of Western Australia.	Supervised aerobic and resistance exercises^a^ in groups Aerobic exercise: 15–20 min at 65–80% of estimated maximum heart rate. Resistance exercise: 2–4 sets of 6–12 RM Flexibility exercises Two times per week	Usual care Control participants could undergo the training after the assessment period had been completed.	12 weeks	Disease-specific quality of life: EORTC QLQ-C30^c^ Health related quality of life: SF-36, physical and mental component Physical function: Repeated chair raise, 5 repetitions Fractures: Number of patients with a fracture Exercise related injuries: Numbers Dropouts: Number of participants
Galvao, 2018, Australia [28] Trial registration: Anztct.org.au identifier: ACTRN 12611001158954	*n* = 57, Tumour stage: T4 Supervised exercise group (*n* = 28) Age, years: 69.7 (7.6) Number on ADT: 27 (96.4) Time on ADT in months, median (IQR): 2.0 (1.0–6.3) Control group (*n* = 29) Age, years: 70.4 (9.3) Number on ADT: 27 (93.1) Time on ADT in days, median IQR): 4.0 (1.0–9.0)	RCT, multicenter, parallel group, two arms. Funding: Prostate Cancer Foundation of Australia	Supervised aerobic and resistance exercise^a^ in groups 10 min warm-up Aerobic exercise: 20–30 min at 60–85% of estimated maximum heart rate. Resistance exercise: three sets of 10–12 RM. 5 min cool down with flexibility exercises 60 min three times per week	Usual care Participants were asked to maintain customary physical activity and dietary patterns over the intervention period.	3 months	Health related quality of life: SF-36, physical component Physical function: 400 m walking test. Fractures: Number of patients with a fracture Exercise related injuries: Number of participants with exercise related injuries. Dropouts: Number of participants.
Harrison, 2018, USA [44] Trial registration: Clinicaltrials.gov identifier: NCT02256111	*N* = 26, Tumour stage: Not clear Supervised exercise group (*n* = 13) Age, years: 65.66 (8.11) Number on ADT: 13 (100) Initiating ADT, exercises began 4 weeks prior to starting ADT Control group (*n* = 13) Age, years: 64.37 (8.31) Number on ADT: 13 (100) Initiating ADT	RCT, parallel group, two arms. Funding: Pheizer (Medivation), Astellas	Supervised aerobic and resistance exercise^a, b^ Aerobic exercise: at 55–80% of estimated maximum heart rate. Resistance exercise: 60–85% of 1 RM. Three times per week.	No supervised exercise training.	16 weeks	Disease-specific quality of life: Fact-P Physical function: 6 min walking test. Physical function: Chair raise test, seconds for five repetitions Muscle strength: Leg press Vo2 peak: ml/Kg/min Fractures: Number of patients with a fracture Exercise related injuries: Numbers Dropouts: Number of participants
Hojan, 2017, Poland [17, 45] Trial registration: ISRCTN.com identifier: ISRCTN80765858 Retrospectively registered	*n* = 72, Tumour stage: T3-T4 Supervised exercise group (*n* = 36) Age, years: 65.7 (6.2) Number on ADT: 36 (100) Control group (*n* = 36) Age, years: 67.9 (4.9) Number on ADT: 36 (100)	RCT, single center, parallel group, two arms. Funding: Greater Poland Cancer Centre	Supervised aerobic exercise^a^ in groups and individual resistance exercises 5 min warm-up Aerobic exercise: 30–40 min at 65–70% of estimated maximum heart rate. Resistance exercise: 2 sets of 8 repetitions at 70–75% of 1 RM. 5 min relaxation period 65 min five times per week for the first 8 weeks and 90 min 3 times per week for the next 10 months	Usual care Standard physical activity recommendations via printed materials to perform 30 min of moderate physical activity 5 days/week and instructions not to begin any formal physical activities and perform usual daily activity at home.	12 months	Disease-specific quality of life: EORTS QLQ-C30^c^ Physical function: 400 m walking test. Muscle strength: Leg press Depressive symptoms: CES-D Fractures: Number of patients with a fracture Exercise related injuries: Numbers Dropouts: Number of participants
Ndjavera, 2020, UK [29] Trial registration: Clinicaltrials.gov identifier: NCT03776045	*n* = 50, Tumour stage: Cancer stage: T3-T4 Supervised exercise group (*n* = 24) Age, years: 71.4 (5.4) Number on ADT: 24 (100) Initiating ADT Control group (*n* = 26) Age, years: 72.5 (4.2) Number on ADT: 26 (100) Initiating ADT	RCT, single center, parallel group, two arms. Funding: Not reported	Supervised aerobic and resistance exercise^a. b^ 5 min warm-up Aerobic exercise: 6 ×5 min at 55–85 % of estimated maximum heart rate. Resistance exercise: 2–4 sets of 10 repetitions at 11–15 of perceived exertion 60 min twice per week Encouraged to increase habitual physical activity and engage in 30 min of self -directed structured exercise/activity 30 min 3 days each week	Standard care. No supervised exercise or specific physical activity recommendations. The control group were offered some supervised exercise sessions after completing the study.	12 weeks	Disease-specific quality of life: Fact-P Muscle strength: Hand grip strength. Vo2 peak: ml/Kg/min Fractures: Number of patients with a fracture Exercise related injuries: Numbers Dropouts: Number of participants.
Newton 2020/Taaffe, 2019, Australia [20, 21] Trial registration: Anztct.org.au identifier: ACTRN 12612000097842	*n* = 104, Tumour stage: T1–T4 Supervised exercise group (*n* = 54) Age, years: 69.0 (6.3) Number on ADT (%): 54 (100) Time on ADT in days: 6.4 (2.1) Control group (*n* = 50) Age, years: 67.5 (7.7) Number on ADT: 50 (100) Time on ADT in days: 5.7 (1.9)	RCT, partial cross over (parallel for our time point of interest), two arms. Funding: Cancer Australia, Prostate Cancer Foundation of Australia and Beyond Blue	Supervised aerobic, impact and resistance exercise^a^ in groups Warm-up Impact exercise: bounding, hopping, skipping, leaping, and drop jumping activities Aerobic exercise: 25–40 min at 60–85% of estimated maximum heart rate. Resistance exercise: 2–4 sets of 6–12 repetitions at 6–12 RM cool down of stretching activities 60 min three times per week.	Usual care/delayed exercise. Usual care for the first 6 months followed by 6 months of supervised exercise (same program as the intervention group, but first after 6 months).	6 months + 6 months. We use data for the end of the first 6 months since the control group hereafter received supervised exercise.	Disease-specific quality of life: EORTC QLQ-C30^c^ Physical function: 400 m walking test. Physical function: Repeated chair raise, 5 repetitions. Muscle strength: Leg press. Fractures: Number of patients with a fracture Exercise related injuries: Numbers Dropouts: Number of participants
Nilsen, 2015, Norway^(18)^ Trial registration: Clinicaltrials.gov identifier: NCT00658229	*N* = 58, Tumour stage: T3–T4 Supervised exercise group (*n* = 28) Age, years: 66 (6.6) Number on ADT: 28 (100) Time on ADT in months: 9.0 (1.6) Control group (*n* = 30) Age, years: 66 (5) Number on ADT: 30 (100) Time on ADT in months: 9.0 (1.8)	RCT, single center, parallel group, two arms. Main funding: Norwegian Foundation of Health and rehabilitation and the Norwegian Cancer Society.	Supervised resistance exercise^a, b^ Resistance exercise: 1–3 sets of 6–10 RM (2 times a week) 2–3 sets of 10 repetitions at 80–90% of 1 RM (ones a week) Three times per week	Usual care	16 weeks	Disease-specific quality of life: EORTC QLQ-C30^c^ Physical function: Repeated chair raise, repetitions in 30 seconds. Muscle strength: Leg press. Fractures: Number of patients with a fracture Exercise related injuries: Numbers Dropouts: Number of participants
Segal 2003, Canada [47] Trial registration: no reference to a protocol	*N* = 155, Tumour stage: T1–T4 Supervised exercise group (*n* = 82) Age, years: 68.2 (7.9) Number on ADT: 82 (100) Time on ADT in months: 12.3 (18.7) Control group (*n* = 73) Age, years: 67.7 (7.5) Number on ADT: 73 (100) Time on ADT in months: 13.2 (21.9)	RCT, multicenter (two sites), parallel group, two arms. Funding: National Cancer Institute of Canada	Supervised resistance exercise^a^, individual training Resistance exercise: 2 sets of 8–12 repetitions at 60–70 % of 1 RM. Three times per week	Waiting list. The control group were offered an identical exercise intervention after the 12-week waiting period.	12 weeks	Disease-specific quality of life: Fact-P Muscle strength: Leg press, repetition maximum Dropouts: Number of participants
Segal 2009, Canada [16] Trial registration: no reference to a protocol	*n* = 121, Tumour stage: T1-T4 Resistance exercise group (*n* = 40) Age, years: 66.4 (7.6) Number on ADT: 23 (57.5) Aerobic exercise group (*n* = 40) Age, years:66.2 (6.8) Number on ADT: 25 (62.5) Control group (*n* = 41) Age, years: 65.3 (7.0) Number of participants on ADT: 26 (63.4)	RCT, multicenter (seven sites), parallel group, three arms. Funding: Canadian Prostate Cancer Research Fund.	Group 1: Supervised resistance exercise^a, b^ 2 sets of 8–12 repetitions at 60–70 % of 1 RM. Group 2: Supervised aerobic exercise^a, b^ at 55–75 % of estimated maximum heart rate. For both groups: Sessions of 15 min increasing to 45 min, three times per week for 24 weeks.	Usual care Participants were asked not to initiate exercise. They were offered a program, post intervention assessments.	24 weeks	Disease-specific quality of life: Fact-P Muscle strength: Leg press. Vo2 peak: ml/Kg/min. Fractures: Number of patients with a fracture Exercise related injuries: Numbers of patients with adverse events related to exercise. Dropouts: Number of participants.
Taaffe 2017/ Newton 2019/ Wall 2017/Galvao 2021 Australia ^46,48,50^ Trial registration: Anztct.org.au identifier: ACTRN 12609000200280 Retrospectively registered	*n* = 163, Tumour stage: T1–T4 Supervised impact loading and resistance exercise (*n* = 58) Age, years: 68 9 (9.1) Number of on ADT: 57 (100) Time on ADT in months: 4.2 (4.5) Supervised aerobic + resistance exercise (*n* = 54) Age, years: 69.0 (9.3) Number on ADT: 54 (100) Time on ADT in months: 5.3 (7.6) Control group (*n* = 58) Age, years: 68.4. (9.1) Number on ADT: 48 (100) Time on ADT in months: 3.7 (3.7)	RCT, multicenter, partial crossover (parallel for our time point of interest), three arms. Funding: National Health and Medical Research Council, Prostate Cancer Foundation of Australia, Cancer Council of Western Australia, and Queensland.	Group 1: Supervised impact loading and resistance exercise^a^ in groups Impact exersice: bounding, hopping, skipping, leaping, and drop jumping activities Resistance exercise: 2–4 sets of 6–12 repetitions at 6–12 RM Group 2: Supervised aerobic and resistance exercise* in groups Aerobic exercise: 20–30 min at 60–75% of estimated maximum heart rate. Resistance exercise: Identical to group 1. For both groups: Sessions twice per week and encouraged to home-based impact r aerobic training twice weekly	Usual care/delayed exercise 6 months of usual care followed by 6 months supervised exercise.	6 months + 6 months. We use data for the end of the first 6 months since the control group hereafter received supervised exercise.	Disease-specific quality of life: EORTC QLQ-C30^c^ Physical function: 400 m walking test.Muscle strength: Leg press. Fractures: Number of patients with a fracture Exercise related injuries: Numbers of participants with adverse events related to exercise. Depressive symptoms: BSI 18 Dropouts: Number of participants
Uth 2014, Denmark [49] Trial registration: Clinicaltrials.gov identifier: NCT01711892	*N* = 57, Tumour stage: T1–T4 Football training group Age, years: 67.1 (7.1) Number on ADT: 29 (100) Time on ADT in days, median (IQR):376 (285–833) Control group Age, years: 66.5 (4.9) Number on ADT: 28 (100) Time on ADT in days, median (IQR): 560 (283–1049)	RCT, multicenter (two sies), parallel group, two arms. Main foundation: Danish Cancer Society and The Novo Nordisk Foundation.	Football training 15 min warm-up (running, dribbling, passing, shooting, balance, and muscle strength exercises) 2–3 ×15 min of 5–7-a-side small-sided football games. 45–60 min two times per week for the first 4 weeks hereafter 3 times per week	Usual care Participants were encouraged to maintain their baseline physical activity level and were offered 12 weeks football training after the assessment period had been completed.	12 weeks	Physical function: Repeated chair raise, repetitions in 30 seconds. Muscle strength: Knee extensor, 1 RM. Vo2 peak: ml/Kg/min. Fractures: Number of patients with a fracture Exercise related injuries: Numbers Dropouts: Number of participants
Via 2021 Australia Trial registration: Anztct.org.au identifier: ACTRN 12614000317695	*N* = 70, Tumour stage: T1-T4 Supervised exercise group (*n* = 34) Age, years: 71.4 (5.9) Number on ADT: 34 (100) Time on ADT in months, median (IQR): 8 (4–22) Control group (*n* = 36) Age, years: 71.1 (6.6) Number on ADT: 36 (100) Time on ADT in months, median (IQR): 13 (8–24)	RCT, single center, parallel group, two arms Funding: Facilities, equipment and internal funding were provided by Deakin University	Supervised aerobic, impact and resistance exercise^a^ Aerobic exercise: 15–25 min at 55–75 % of estimated maximum heart rate. Resistance exercise: 2 sets of 8–12 repetitions. (3–6 on the 10-point Rating of Perceived Exertion (RPE) scale Impact exercise: e.g. jumping, hopping, step-ups. Multi-nutrient supplement consisting of protein, calcium and vitamin D. 60 min per session Two supervised and one home-based session per week for the first 26 weeks. Hereafter only one supervised session per week	Usual care Ongoing care from a physician/specialist + vitamin d but received no additional education or access to exercise training or the protein-, calcium- and vitamin D-enriched nutritional supplement powder sachet	12 months	Physical function: 400 m walking test. Muscle strength: Leg press Fractures: Number of patients with a fracture Exercise related injuries: Number of participants with adverse events related to exercise. Dropouts: Number of participants
Winterstone 2015, USA [51, 52] Trial registration: Clinicaltrials.gov identifier: NCT00660686	*n* = 51, Tumour stage: T1–T4 Supervised exercise group (*n* = 29) Age, years: 69.9 (9.3) Number on ADT: 29 (100) Time on ADT in months: 39.0 (36.1) Control group (*n* = 22) Age, years: 70.5 (7.8) Number on ADT: 22 (100) Time on ADT in months: 28.5 (29.2)	RCT, single center, parallel group, two arms Funding: Live strong Foundation and TheraBand (providing exercise equipment, elastic bands)	Supervised resistance and impact exercise^a^ Resistance exercise: 8–15 repetition maximum Impact exercise: 50 two-footed jumps 30–60 min per session Two supervised and one home-based session per week	Placebo exercises Whole-body stretching and relaxation exercises aimed to minimize weight-bearing forces and muscle activation. A gentle exercise placebo group was used to equalize attention, maximize retention, and minimize contamination. Two supervised and one home-based session per week session	12 months	Disease-specific quality of life: EORTC QLQ-C30^d^ Physical function: Repeated chair raise, 5 repetitions. Physical function: Walking test, 4 meters, usual pace. Muscle strength: Leg press. Fractures: Number of patients with a fracture Exercise related injuries: Numbers of patients with adverse events related to exercise. Dropouts: Number of participants.

*BSI 18* Brief Symptom Inventory 18, *CES-D* Epidemiologic Studies depression Scale, *EORTC QLQ-C30* European Organization for the Research and Treatmant of Cancer Quality of Life Questionnaire Core 30, *Fact-P* Functional Assessment of Cancer Therapy-Prostate, *SF-36* 36-Item Short Form Health Survey.

^a^Both aerobic and resistance exercises were individually progressed, the resistance exercise comprised of 5–10 different exercises targeting both the upper and lower extremity.

^b^No information on group or individual exercises.

^c^Global Health Status and quality of life subscsale.

^d^Physical function subscale.

### Quality assessment

The AMSTAR evaluation of the included systematic review revealed that the review had adequate description of nine out of 11 domains (Supplementary Table S2). Concerning the rigour and transparency of the literature search and inclusion of primary trials (domain 1–4), we judged that the review was of sufficient quality to enable us to base our search for primary trials on their last search date.

### Risk of bias assessment

**Fig. 1:**
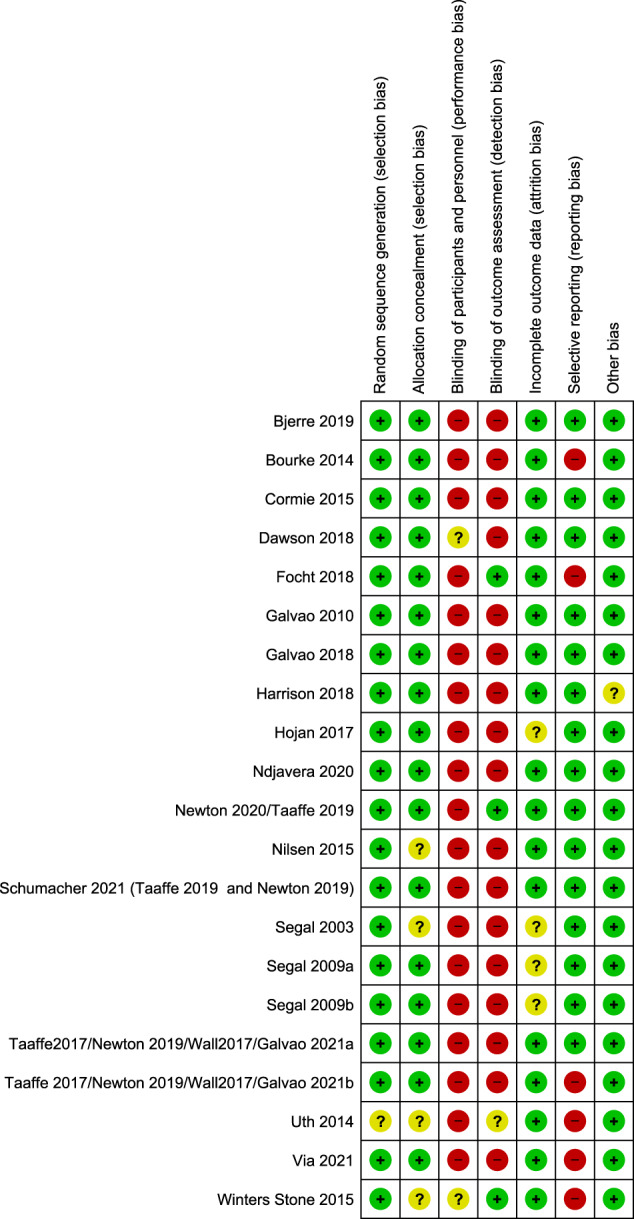
Risk of bias assessment of included trials. Assessed by the Cochrane risk of bias tool. Green (+): indicates low risk of bias, red (-): indicates high risk of bias, yellow (?): indicates unclear risk of bias.

Low risk of selection bias were assessed in 14 trials [17, 18, 20–22, 27–31, 45–50, 52, 54], and four trials [19, 51, 53, 56] had unclear risk of selection bias due to inadequate reporting regarding random sequence generation and/or allocation concealment. All included trials were assessed to have high risk of performance bias due to lack of blinding of participants and personnel, since blinding for the intervention was not feasible. Thirteen trials [17, 18, 21, 22, 27, 29, 30, 45–50, 52, 54, 57, 58] had high risk of detection bias, since the critical outcome ‘disease-specific quality of life’ was self-reported and participants not blinded. Three trials had unclear risk of attrition bias (incomplete outcome data) [17, 49, 51]. In total, five trials [20, 31, 53, 55, 56] were assessed to have high risk of selective reporting, primary due to omission of reporting ‘quality of life’ despite the fact that the outcome was stated in a protocol [20, 31, 53, 55, 56]. One trial was reported only in an abstract and at clinicaltrials.gov and had unclear risk of other bias, due to inadequate reporting [48]. See the risk of bias assessment in Fig. 1.

### Certainty of evidence (GRADE)

The results of the GRADE-process are shown in Table 2. The certainty of the evidence for the two critical outcomes ‘disease-specific quality of life’ and ‘physical performance, walking performance’ was downgraded one level due to serious risk of bias because of lack of blinding of participants and self-reported measures of disease-specific quality of life. Thus, the overall certainty of evidence for supervised exercise therapy compared with no exercise therapy was moderate. Since the funnel plots did not suggest publication bias, no downgrading for this item was performed (Supplementary Figures S3).

**Table 2:** GRADE Summary of Finding Table. Supervised exercise therapy compared to no exercise therapy for patients with prostate cancer receiving androgen deprivation therapy. Population: Patients with prostate cancer receiving androgen deprivation therapy. Intervention: Supervised exercise therapy. Comparison: No exercise therapy.

Outcomes	Anticipated absolute effects^*^ (95% CI)		Relative effect (95% CI)	№ of participants (studies)	Certainty of the evidence (GRADE)	Comments
	Risk with No exercise	Risk with supervised exercise				
Diagnose-specific quality of life (critical outcome)	–	SMD 0.43 higher (0.29 higher to 0.58 higher)	–	894 (12 RCTs)	⨁⨁⨁◯ MODERATE^a^	Supervised exercise therapy probably increases diagnose-specific quality of life.
Health related quality of life (important outcome) assessed with: SF-36, physical component Scale from: 0 to 100	The mean health related quality of life was 44.8	MD 1.34 higher (1.99 lower to 4.67 higher)	–	246 (4 RCTs)	⨁⨁⨁◯ MODERATE^a^	Supervised exercise therapy probably results in little to no difference in health related quality of life, SF-36, physical component.
Health related quality of life (important outcome) assessed with: SF-36, mental component	The mean health related quality of life was 49.2	MD 3.30 higher (0.87 higher to 5.74 higher)	–	198 (3 RCTs)	⨁⨁⨁◯ MODERATE^a^	Supervised exercise therapy probably results in little to no difference in health related quality of life, SF-36 mental component.
Physical performance, walking performance (critical outcome)	–	SMD 0.41 lower (0.60 lower to 0.22 lower)	–	667 (11 RCTs)	⨁⨁⨁◯ MODERATE^b^	Supervised exercise therapy probably improves physical performance, walking performance.
Physical performance, sit to stand (important outcome)	–	SMD 0.35 higher (0.14 higher to 0.56 higher)	–	463 (8 RCTs)	⨁⨁◯◯ LOW^b,c^	Supervised exercise therapy may result in an improvement in physical performance, sit to stand.
Muscle strenght (important outcome)	–	SMD 0.47 higher** (0.28 higher to 0.65 higher)	–	968 (15 RCTs)	⨁⨁⨁◯ MODERATE^b^	Supervised exercise therapy probably increases muscle strength.
VO2 peak (important outcome)	The mean VO2 peak was 0	MD 1.76 higher (0.82 higher to 2.69 higher)	–	406 (6 RCTs)	⨁⨁⨁◯ MODERATE^b^	Supervised exercise therapy probably increases VO2 peak.
Prevalence of depression (important outcome) assessed with: Depressive symptoms	–	SMD 0.23 lower (0.54 lower to 0.08 higher)	–	195 (3 RCTs)	⨁◯◯◯ VERY LOW^a,d,e^	The evidence is very uncertain about the effect of supervised exercise on depression.
Fractures, number of patients (important outcome)	2 per 1.000	1 more per 1.000*** (14 fewer to 16 more)	RR 1.86 (0.25 to 13.99)	1131 (17 RCTs)	⨁⨁◯◯ LOW^c,f^	The evidence is very uncertain about the effect of supervised exercise therapy on fractures.
Exercise related injuries), number of patients, risk ratio analysis (important outcome)	0 per 1.000	9 more per 1.000*** (9 fewer to 28 more)	RR 5.86 (1.55 to 22.06)	940 (15 RCTs)	⨁⨁⨁◯ MODERATE^c^	Supervised exercise probably increases exercise related injuries slightly.
Dropout all causes, risk ratio analysis	150 per 1.000	40 fewer per 1.000 (67 fewer to 0)	RR 0.73 (0.54 to 0.96)	1487 (16 RCTs)	⨁⨁⨁◯ MODERATE^c,g^	Supervised exercise probably results in little to no difference in dropout all causes

CI: confidence interval, GRADE: Grades of Recommendation, Assessment Development and Evaluation, SMD: standardised mean difference, MD: mean difference, RR: risk ratio

*The risk in the intervention group (and its 95% confidence interval) is based on the assumed risk in the comparison group and the relative effect of the intervention (and its 95% CI).

**A subgroup analysis was performed to explain moderate heterogeneity (I^2^ 48%). Dividing the trials in to type of exercises (aerobic, resistance, combined and football) did not reveal significant subgroup effects (*P* value 0.11) but reduced the heterogeneity to 0–1% among trials of combined exercise and football training. Among resistance trials the heterogeneity was substantial (I^2^ 69%). This was explained by the intensity of the resistance exercise. By excluding two high intensity trials the heterogeneity was reduced to 0% both in the subgroup analysis and the total analysis. Since the heterogeneity could be explained, we did not downgrade for inconsistency.

In a subgroup analysis including only resistance trials and dividing the trials into high and moderate intensity, we found a significant (subgroup effect *p* = 0.0004). The results for trials with moderate intensity was a SMD of 0.41 (95% CI: 0.19, 0.63) and for the high intensity trials the SMD was 1.44 (95% CI: 0.72, 0.63).

***The absolute numbers are calculated based on a risk difference analysis.

*Abbreviation: CI* Confidence interval, *GRADE* Grades of Recommendation, Assessment, Development and Evaluation, *SMD* Standardised mean difference, *MD* Mean difference, *RR* Risk ratio.

GRADE Working Group grades of evidence. High certainty: We are very confident that the true effect lies close to that of the estimate of the effect. Moderate certainty: We are moderately confident in the effect estimate. The true effect is likely to be close to the estimate of the effect, but there is a possibility that it is substantially different. Low certainty: Our confidence in the effect estimate is limited. The true effect may be substantially different from the estimate of the effect. Very low certainty: We have very little confidence in the effect estimate. The true effect is likely to be substantially different from the estimate of effect.

^a^Lack of blinding of personnel and participants and self-reported outcome.

^b^Lack of blinding of personnel and participants, lack of blinding in the outcome assessment.

^c^Wide CIs, CIs is overlapping the minimal clinical important difference.

^d^Difference between relevant and reported outcomes, our outcome of interest was prevalence of depression, the trials measure depressive symptoms.

^e^Few patients included in the trials (<100).

^f^Few events.

^g^Wide confidence interval, but the interval is not inaccurate in relation to a recommendation. The result does not indicate the supervised exercise therapy leads to increase in dropouts.

### Results for critical outcomes

**Fig. 2:**
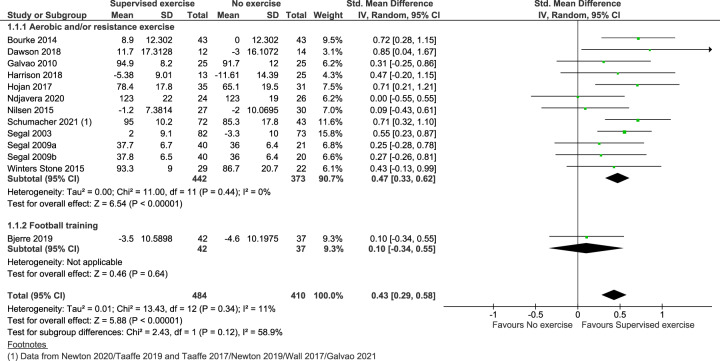
Forest plot of the critical outcome ‘disease-specific quality of life’. ADT: androgen deprivation therapy, CI: confidence interval, df: degrees of freedom, EORTC-CLQ-C30: The European Organization for Research and Treatment EORTC core quality of life questionnaire, Fact-P: The Functional Assessment of Cancer Therapy - Prostate (range 0-156), Std: standardised.

**Fig. 3:**
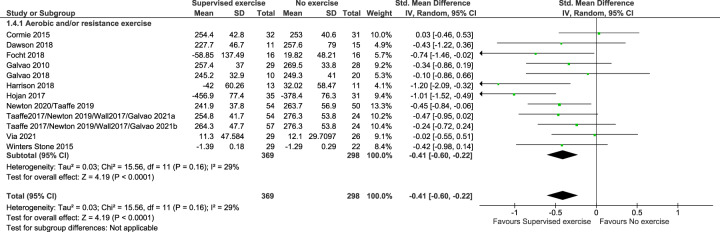
Forest plot of the critical outcome ‘physical performance’ measured by walking performance. ADT: androgen deprivation therapy, C:I confidence interval, df: degrees of freedom, Std: standardised.

For the critical (primary) outcome ‘disease-specific quality of life’ we found that supervised exercise therapy resulted in clinically relevant improvements compared to no exercise therapy. The standardised mean difference (SMD) was 0.43 (95% CI: 0.29, 0.58), see Fig. 2 and Table 2. When transformed to a mean estimate, the result corresponded to a mean improvement of 8 points on FACT-P (95% CI: 6, 11), range 0-156, Minimum Clinically Important Difference (MCID) 6–10 points [39]. The other critical outcome ‘physical performance measured by walking performance’ also showed clinically relevant improvements in favour of supervised exercise therapy, SMD was −0.41 (95 % CI: −0.60, − 0.22), see Fig. 3 and Table 2. The result corresponded to a mean reduction of 23 s on 400 m walking test (95 % CI: 13, 34), MCID 20–30 s [40]. The certainty of the evidence for the two critical outcomes was moderate.

### Results for important outcomes

The results for the important (secondary) outcomes are shown in Table 2 and the forest plots are shown in Supplementary Figures S3.

Evaluation of ‘physical performance measured by sit to stand performance’ showed a possible difference in effect in favour of supervised exercise therapy compared to no exercise therapy, SMD was 0.35 (95% CI: 0.14, 0.56) (low certainty), corresponding to one extra repetition on 30-second sit to stand test. This is just below the MCID of two repetitions on this test [59].

Evidence of moderate certainty showed that supervised exercise therapy improved muscle strength (SMD 0.47, 95% CI: 0.28, 0.65) and VO2 peak (MD 1.76 ml/kg/m, 95% CI: 0.82, 2.69) compared to no exercise therapy. The latter corresponds to an improvement of 8% (95% CI: 4%, 13%) compared to no exercise therapy, and the guideline panel considered this to represent a clinically relevant improvement for the examined population.

Supervised exercise therapy did not imply higher dropout compared to no exercise therapy (risk ratio 0.73, 95% CI: 0.54, 0.96) (moderate certainty). The absolute difference was 40 fewer dropouts per 1000 (95% CI: 67 fewer to zero) with supervised exercise therapy. The pre-planned subgroup analysis revealed a significant lower risk of dropping out when supervised exercise therapy started within one month after starting ADT (P-value 0.04). The risk ratio was 0.36 (95% CI: 0.18, 0.73) for early start vs 0.82 (95% CI: 0.61, 1.11) for later start of supervised exercise therapy.

More people had training related injuries with supervised exercise therapy compared to no exercise therapy, (risk ratio 5.86, 95% CI: 1.55, 22.06) but the absolute number of persons with injuries was low, nine per 1000 with supervised exercise therapy compared to zero per 1000 with no exercise therapy. One trial of football training was not included in the analysis since data were only reported for the football group [27]. The trial reported 60 training related injuries (19 overuse injuries and 41 acute injuries) among the 109 participants in the football group (41% on ADT).

Sixteen trials reported data regarding adverse events. Fractures were reported as adverse events in the two football trials. The analysis revealed an increased risk of fractures for supervised exercise therapy, the risk ratio was 1.86 (95% CI: 0.25, 13.99), but the absolute numbers were very low. Three persons with fractures per 1000 with supervised exercise therapy compared to two per 1000 with no exercise therapy. The absolute difference was one more with a fracture per 1000 with supervised exercise therapy (95% CI: 14 fewer to 16 more).

The important outcomes ‘prevalence of cardiovascular disease’, ‘prevalence of diabetes’ and ‘prevalence of depression’ were not reported in the included trials. Two trials reported data for depressive symptoms, these data were used as indirect evidence for the outcome ‘prevalence of depression’. The results showed no statistical significant or clinically relevant differences (very low certainty).

### Sensitivity and subgroup analyses

Sensitivity analyses with exclusion of one trial where only 61% of the population received ADT [17], did not change any results to a significant degree. No meaningful subgroup analysis regarding individual vs group exercise could be conducted, since only one trial reported individual exercise therapy. The other pre-planned subgroup analyses revealed no additional significant subgroup differences, besides the subgroup effect for dropout reported above.

## Discussion

Based on evidence of moderate quality our results show that supervised exercise therapy was superior to no exercise therapy on the two critical outcomes ‘diagnose-specific quality of life’ and ‘physical performance, walking performance’. The calculated SMD of 0.43 for ‘diagnose-specific quality of life’ was both statistically significant and clinically relevant. When transformed to MD, the result corresponded to an improvement of 8 point on FACT-P in favour of exercise therapy. This is above the MCID of 6–10 points [39].

Other authors have found effects on disease-specific quality of life somewhat smaller than we did [16, 25, 60, 61], but Teleni et al. reported results in line with ours [24]. In a meta-analysis not restricted to patients on ADT, Bourke et al. found no significant effect of exercise compared to usual care on cancer-specific quality of life, but in a sensitivity analysis restricted to high quality trials, they found a statistically significant result similar to ours [16]. The differences in results across meta-analyses could mainly be due to variations in populations [16, 60, 61] and inclusion criteria [16, 25, 60, 61].

Regarding the critical outcome ‘physical performance, walking performance’ we found a statistically significant and clinically relevant SMD of −0.41 in favour of exercise therapy. When transformed to MD, the estimate corresponded to a mean reduction of 23 s on 400 m walking test and lies within the range of the estimated MCID [40]. Our result is in line with Bourke et al. who evaluated sub-maximal aerobic fitness (primarily including outcomes for 400 meters walking test) and found an SMD of −0.49 [16]. In contrast, Keilani et al. reported a result just below the MCID with a reduction of 18 s [62]. Keilani et al. included trials with resistance exercises not restricted to RCT-designs and not limited to patients on ADT. These differences could explain variations in results.

The included trials comprised patients with stage T1-T4 prostate cancer and the heterogeneity in all analyses for critical outcomes and in nearly all analyses of the important outcomes were very low, meaning there was no important systematic variation between effect sizes in the included trials. This suggests that our results probably are appropriate for all patients with prostate cancer receiving ADT regardless of stages of prostate cancer.

The debilitating side effects of ADT leading to limitations in physical performance probably is an important factor in reducing quality of life in patients with prostate cancer on ADT. Performance status have been stated as a critical factor for quality of life among cancer patients [63], and increases in physical performance have been suggested to be directly related to maintenance or improvement of quality of life among cancer patients [62]. At the same time, physical exercises are proposed as the most important intervention to mitigate psychological side effects of ADT [64]. We did not analyze whether improvements in physical performance mediated the shown improvement in disease-specific quality of life, but it seems reasonable as improved physical performance can enable increased participation in everyday life. Other mechanism of actions could be improved self-efficacy and psychological benefits of interaction with other patients.

More people experienced exercise related injuries with supervised exercise therapy compared to no exercise therapy, but the absolute number of injuries was low. Thus, it appears that the number of injuries is not higher among persons on ADT compared to any other person participating in exercise therapy.

We found an increased risk of fractures with supervised exercise therapy compared to no exercise. Fractures were only reported in trials with football interventions. The certainty in the estimate was low and thus it is uncertain whether supervised exercise therapy protects against fractures in this population, as well as it is uncertain whether football entails a risk of fractures. The included trials were underpowered to detect an effect on fractures. A larger population and long follow-up may be needed to show the effect of exercise therapy on fractures.

When offering supervised exercise therapy especially to untrained persons, one should take into account, that there might be an increased risk of exercise related injuries with football and that it is uncertain whether football entails a risk of fractures. One should consider recommending other exercise modalities to persons not used to football training.

In pre-planned subgroup analyses, we found that early start of exercise therapy is just as effective as later commencement (all outcomes). Interestingly, we found that early start significantly reduced the risk of dropout. As delayed exercise therapy postpones the positive effect of exercise [21, 22, 50, 52], it should be recommended to start exercise therapy with the initiation of ADT [21].

We did not find any trials evaluating the effect of exercise therapy on prevalence of cardiovascular diseases, diabetes and depression. Similar to fractures, larger populations and longer follow-up may be needed to evaluate the effect on these outcomes.

### Strengths and limitations

This review was conducted using rigor and transparent methods in accordance with the Cochrane collaboration, the PRISMA recommendations and the GRADE-method. The strength of the GRADE-method includes pre-specification of the inclusion criteria and pre-assessment of critical and important outcomes, judged as critical or important to patients. This process ideally involves asking patients, as we did in our work. By this, we may have reduced the risk of using outcomes less relevant to patients. The GRADE-method represents a rigour and transparent method of formulation the research question (PICOT) and assessing the certainty in the evidence by assessing risk of bias, inconsistency, indirectness, imprecision and publication bias both per outcome and as overall certainty. Furthermore, we conducted a systematic literature search, and two independent reviewers conducting trial selection, data extraction and quality assessments. Limitations were literature searches restricted to English, Danish, Swedish and Norwegian language. Furthermore, the search for primary trials was based on the last search date of the included systematic review [16]. This may have resulted in not identifying all older relevant primary trials. However, supplemental hand searches for primary trials in both systematic reviews and primary trials have probably limited this risk.

All included trials were assessed to have high risk of performance bias due to lack of blinding of participants and personnel. We could have chosen not to downgrade for this aspect, since it could be argued that blinding for an exercise intervention is not feasible [16]. However, since lack of blinding can still affect the professionals delivering the intervention and the assessment of self-reported outcomes, we decided to maintain this assessment. Despite this, the certainty in the evidence was still moderate.

## Conclusion

Evidence of moderate quality shows that supervised exercise therapy is superior to no exercise therapy in improving ‘disease-specific quality of life’ and ‘physical performance’ measured by walking performance in patients with prostate cancer undergoing ADT. The results apply to all patients receiving ADT regardless of cancer stage. Based on moderate certainty of the evidence, the results support a strong recommendation of supervised exercise therapy for managing side effects of ADT in this population. To avoid postponement of the positive effects of supervised exercise therapy and to reduce dropouts, it should be recommended to start exercise therapy when initiating ADT.

## Supplementary information

Supplemental material

PRISMA checklist

## Data Availability

Data are available for bona fide researchers who request it from the authors.
