# Supplementary material for: Supervised exercise therapy compared with no exercise therapy to reverse debilitating effects of androgen deprivation therapy in patients with prostate cancer: a systematic review and meta-analysis
Source: Prostate Cancer Prostatic Dis. 2021 Sep 6;25(3):491–506. doi: 10.1038/s41391-021-00450-0 (PMC9385477; doi:10.1038/s41391-021-00450-0)
Supplement: Supplementary file 1 — Supplemental material [file 41391_2021_450_MOESM1_ESM.docx]

**Supplementary figure S1**


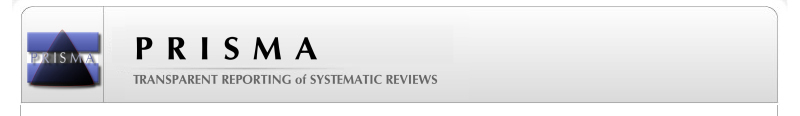
**PRISMA 2009 Flow Diagram**

**Search for systematics reviews (performed 18^th^ January 2016)**

Primary trials included from the identified systematic review (n = 7)

Full-text articles excluded, with reasons

(n = 67)

Wrong study design (n=19)

Wrong population (n=20)

Wrong intervention (n=21)

Wrong outcome (n=1)

Abstract (n=6)

Systematic review included
(n = 1)

Full-text articles assessed for eligibility
(n = 68)

Records excluded
(n = 644)

Records screened
(n = 732)

Records after duplicates removed
(n = 732)

Additional records identified through other sources
(n = 0)

Records identified through database searching
(n = 1,064)

## Identification

## Screening

## Eligibility

## Included

**Supplementarty Figure S2a**


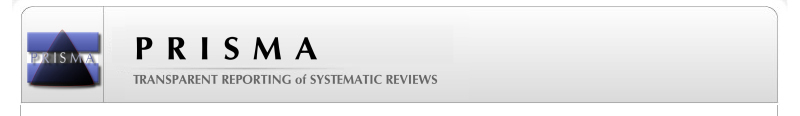
**PRISMA 2009 Flow Diagram**

**Search for primary studies (performed 24^th^ February 2016)**

Full-text articles assessed for eligibility
(n = 17)

Records excluded
(n = 132)

Records screened
(n = 149)

Records after duplicates removed
(n = 149)

Additional records identified through other sources
(n = 0)

Records identified through database searching
(n = 233)

## Identification

## Screening

## Eligibility

Full-text articles excluded, with reasons
(n = 16)

A protocol (n=4)

Wrong population (n=5)

Wrong intervention (n=4)

Study already included from the systematic review by Bourke et al. (n=3)

Primary studies included
(n = 1 )

## Included

**Supplementary figure S2b**


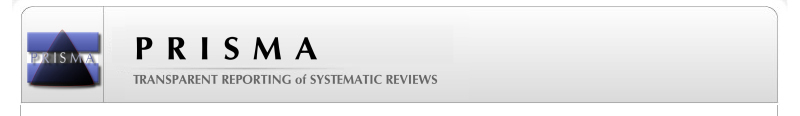
**PRISMA 2009 Flow Diagram**

**Updated search for primary studies**

**(performed 16^th^ June 2021)**

Full-text articles assessed for eligibility
(n = 109)

Records excluded
(n = 762)

Records screened
(n = 871)

Records after duplicates removed
(n = 871)

Additional records identified through other sources
(n = 0)

Records identified through database searching
(n = 1.554)

## Identification

## Screening

Full-text articles excluded, with reasons
(n = 94)

Wrong study design (n=30)

Wrong population (n=22)

Wrong intervention (n=22)

Wrong comparison (n=6)

Wrong outcomes (n=12)

Duplicate (n=2)

## Eligibility

Primary studies included
(n = 10) 15 publications

## Included

**Supplementary Table S1. List of excluded articles after full text-review**

| No. | Study (author and year) | Reference | Reason for exclusion |
| --- | --- | --- | --- |
| 1 | Aggarwal 2015 | Aggarwal R.R.; Rodvelt T.J.; Rabow M.W.; Macaire G.; Fedric R.; CorteseJimenez G.; Small, E. J.. A multidisciplinary clinic to mitigate the impact of androgen deprivation therapy in prostate cancer: A pilot study. Journal of Clinical Oncology 2015; Conference: 2015 Genitourinary Cancers Symposium. Orlando, FL United States. Conference Publication (var.pagings):date of Publication: 01 Mar 2015. | Wrong intervention, counselling no supervised training |
| 2 | Alibhai 2015 | Alibhai,Shabbir M. H.; Santa Mina,Daniel; Ritvo,Paul; Sabiston,Catherine; Krahn,Murray; Tomlinson,George; Matthew,Andrew; Segal,Roanne; Warde,Padraig; Durbano,Sara; O'Neill,Meagan; Culos-Reed,Nicole. A phase II RCT and economic analysis of three exercise delivery methods in men with prostate cancer on androgen deprivation therapy. BMC Cancer 2015;15(Journal Article):312England 2015. | A protocol |
| 3 | Alibhai 2018a | Alibhai S.M.H.; Durbano S.; O'Neill M.; Mina D.S.; Ritvo P.; Sabiston C.; Krahn M.D.; Tomlinson G.A.; Matthew A.; Warde P.R.; Timilshina N.; Segal R.; Nicole CulosReed, S. Effects of a 6-month moderate-intensity exercise program on metabolic parameters and bone mineral density in men on androgen deprivation therapy for prostate cancer. Journal of Clinical Oncology 2018; Conference: 2018 Genitourinary Cancers Symposium. United States (Journal Article): date of Publicaton: February 2018. | Abstract, fulltext available Alibhai 2019, |
| 4 | Alibhai 2018b | Alibhai, Shabbir M. H.; Ritvo, Paul; Santa Mina, Daniel; Sabiston, Catherine; Krahn, Murray; Tomlinson, George; Matthew, Andrew; Lukka, Himu; Warde, Padraig; Durbano, Sara; O'Neill, Meagan; Culos-Reed, S.. Protocol for a phase III RCT and economic analysis of two exercise delivery methods in men with PC on ADT. BMC Cancer 2018;18(1):1-18. | A protocol |
| 5 | Alibhai 2019 | Alibhai, Shabbir M. H.; Santa Mina, Daniel; Ritvo, Paul; Tomlinson, George; Sabiston, Catherine; Krahn, Murray; Durbano, Sara; Matthew, Andrew; Warde, Padraig; O'Neill, Meagan; Timilshina, Narhari; Segal, Roanne; Culos-Reed, Nicole. A phase II randomized controlled trial of three exercise delivery methods in men with prostate cancer on androgen deprivation therapy. BMC Cancer 2019;19(1):1-11. | Wrong comparator, three groups, all groups receive supervision |
| 6 | Armes 2016 | Armes J.; Harris J.; Tsianakas V.; Ream E.; Van Hemelrijck M.; Green J.; Purusthotham A.; Mucci L.; Robb K.; Fewster, J.. Can walk: A randomised feasibility trial of a walking intervention for people with recurrent or metastatic cancer. Psycho-oncology 2016; Conference: 2016 World Congress of Psycho-Oncology. Ireland (Journal Article): date of publication: Otober 2016. | Wrong intervention, recommendation of physical activity and a motivational interveiw |
| 7 | Asthon 2021 | Ashton, Ruth E.; Aning, Jonathan J.; Tew, Garry A.; Robson, Wendy A.; Saxton, John M.  Supported progressive resistance exercise training to counter the adverse side effects of robot-assisted radical prostatectomy: a randomised controlled trial. Supportive Care in Cancer 2021;(Journal Article): 2021 | Wrong patient population, patients treated with prostatectomy excluding patient receiving any other treatment |
| 8 | Baguley 2017 | Baguley, Brenton J.; Skinner, Tina L.; Leveritt, Michael D.; Wright, Olivia R. L. Nutrition therapy with high intensity interval training to improve prostate cancer-related fatigue in men on androgen deprivation therapy: a study protocol. BMC Cancer 2017;17 (Journal Article):1-12. [DOI: 10.1186/s12885-016-3022-6] | A protocol |
| 9 | Bjerre 2021 | Bjerre, Eik Dybboe; Weller, Sarah; Poulsen, Mads Hvid; Madsen, Soren Sorensen; Bjerre, Rie Dybboe; Ostergren, Peter Busch; Borre, Michael; Brasso, Klaus; Midtgaard, Julie  Safety and Effects of Football in Skeletal Metastatic Prostate Cancer: a Subgroup Analysis of the FC Prostate Community Randomised Controlled Trial.  Sports Medicine - Open 2021;7(1):27  2021 | Subgroup analysis, primary trial already included |
| 10 | Bjorke 2020 | Bjorke, Ann Christin Helgesen; Raastad, Truls; Berntsen, Sveinung. Criteria for the determination of maximal oxygen uptake in patients newly diagnosed with cancer: Baseline data from the randomized controlled trial of physical training and cancer (Phys-Can). PLoS ONE [Electronic Resource] 2020;15(6):e0234507. | Wrong study design, baseline data from a RCT-study of patients newly diagnosed with cancer, 17 % prostatcancer, no information on ADT. |
| 11 | Bourke 2018 | Bourke, L.; Stevenson, R.; Turner, R.; Hooper, R.; Sasieni, P.; Greasley, R.; Morrissey, D.; Loosemore, M.; Fisher, A.; Payne, H.; Taylor, S. J. C.; Rosario, D. J.. Exercise training as a novel primary treatment for localised prostate cancer: a multi-site randomised controlled phase II study. Scientific Reports 2018;8(1):8374. | Wrong patient population, no ADT |
| 12 | Brady 2019 | Brady L.; Hayes B.; Sheill G.; Baird A.M.; Guinan E.M.; Stanfill B.; Vlajnic T.; Casey O.; Greene J.P.; Allott E.; Hussey J.M.; Cahill F.; Van Hemelrijck M.; Peat N.; Rudman S.M.; Mucci L.; Sheils O.; John O.; McDermott R.; Finn, S. P. The effect of a structured exercise intervention on CTCs and platelet cloaking in patients with metastatic prostate cancer. Journal of Clinicak Oncology. Volumen 37, Issue 7, Suppl. | An abstract, no information on ADT and wrong outcomes |
| 13 | Brady 2020 | Brady, Lauren; Hayes, Brian; Sheill, Grainne; Baird, Anne-Marie; Guinan, Emer; Stanfill, Bryan; Vlajnic, Tatjana; Casey, Orla; Murphy, Verena; Greene, John; Allott, Emma H.; Hussey, Juliette; Cahill, Fidelma; Van Hemelrijck, Mieke; Peat, Nicola; Mucci, Lorelei; Cunningham, Moya; Grogan, Liam; Lynch, Thomas; Manecksha, Rustom P.; McCaffrey, John; O'Donnell, Dearbhaile; Sheils, Orla; O'Leary, John; Rudman, Sarah; McDermott, Ray; Finn, Stephen  Platelet cloaking of circulating tumour cells in patients with metastatic prostate cancer: Results from ExPeCT, a randomised controlled trial.  PLoS ONE [Electronic Resource] 2020;15(12):e0243928  2020 | Wrong outcomes |
| 14 | Buffart 2015 | Buffart, Laurien M.; Newton, Robert U.; Chinapaw, Mai J.; Taaffe, Dennis R.; Spry, Nigel A.; Denham, James W.; Joseph, David J.; Lamb, David S.; Brug, Johannes; Galvao, Daniel A. The effect, moderators, and mediators of resistance and aerobic exercise on health-related quality of life in older long-term survivors of prostate cancer. Cancer 2015; 121(16):2821-2830. | Wrong patient population |
| 15 | Chan 2020 | Chan, June M.; Van Blarigan, Erin L.; Langlais, Crystal S.; Zhao, Shoujun; Ramsdill, Justin W.; Daniel, Kimi; Macaire, Greta; Wang, Elizabeth; Paich, Kellie; Kessler, Elizabeth R.; Beer, Tomasz M.; Lyons, Karen S.; Broering, Jeanette M.; Carroll, Peter R.; Kenfield, Stacey A.; Winters-Stone, Kerri M.  Feasibility and Acceptability of a Remotely Delivered, Web-Based Behavioral Intervention for Men With Prostate Cancer: Four-Arm Randomized Controlled Pilot Trial.  Journal of Medical Internet Research 2020;22(12):e19238  12 31 2020 | Wrong patient population |
| 16 | Cole 2017a | Cole C.; Peppone L.J.; Kleckner I.; Lin P.J.; Dunne R.F.; Fung C.; Loh K.P.; Asare M.; Mustian K.M.; Morrow, G. R.. Effects of exercise on dyspnea and cancer-related fatigue in patients with prostate cancer. Journal of Clinical Oncology 2017; Conference: 2017 Annual Meeting of the American Society of Clinical Oncology, ASCO. United States (Journal Article): date of Publication: 20 Jun 2017. | An abstract, no usable data |
| 17 | Cole 2017b | Cole C.; Peppone L.; Kamen C.; Fung C.; Loh K.; Dunn R.; Kleckner I.; Lin P.J.; Asare M.; Janelsins M.; Mustian, K. Interrelationship between improvements in aerobic capacity and cancer-related-fatigue in prostate cancer patients. Supportive Care in Cancer 2017; Conference: 2017 International MASCC/ISOO Symposium: Supportie Care in Cancer. United States (Journal Article): date of Publication: 2017. | An abstract, No information on ADT. |
| 18 | Cormie 2013 | Cormie,P.; Newton,R. U.; Spry,N.; Joseph,D.; Taaffe,D. R.; Galvao,D. A. Safety and efficacy of resistance exercise in prostate cancer patients with bone metastases. Prostate cancer and prostatic diseases 2013;16(4):328-335. [DOI: 10.1038/pcan.2013.22 | Preliminary publication of a study. Primary publication already included Cormie2015 |
| 19 | Craike 2016 | Craike, Melinda; Gaskin, Cadeyrn J.; Courneya, Kerry S.; Fraser, Steve F.; Salmon, Jo; Owen, Patrick J.; Broadbent, Suzanne; Livingston, Patricia M. Predictors of adherence to a 12-week exercise program among men treated for prostate cancer: ENGAGE study. Cancer Medicine 2016; 5(5):787-794. | Wrong patient population, only a minority of the patients receive ADT |
| 20 | Craike 2018 | Craike, Melinda J.; Gaskin, Cadeyrn J.; Mohebbi, Mohammadreza; Courneya, Kerry S.; Livingston, Patricia M.. Mechanisms of Physical Activity Behavior Change for Prostate Cancer Survivors: A Cluster Randomized Controlled Trial. Annals of Behavioral Medicine 2018;52(9):798-808. | Wrong patient population, only a minority of the patients receive ADT |
| 21 | Cunningham 2020 | Cunningham E.; Weaver R.R.; Lemonde M.; Dogra S.; Nonoyama, M. L. Nordic Pole Walking for Individuals with Cancer: A Feasibility Randomized Controlled Trial Assessing Physical Function and Health-Related Quality of Life. Rehabilitation Oncology 2020;38(2):81-91. | Wrong patient population, cancer patients |
| 22 | Dieperink 2013 | Dieperink,K. B.; Johansen,C.; Hansen,S.; Wagner,L.; Andersen,K. K.; Minet,L. R.; Hansen,O.. The effects of multidisciplinary rehabilitation: RePCa-a randomised study among primary prostate cancer patients. British journal of cancer 2013;109(12):3005-3013. | Wrong intervention, multidisciplinary intervention, primary pelvic floor muscle training |
| 23 | Dieperink 2017 | Dieperink, Karin B.; Johansen, Christoffer; Hansen, Steinbjorn; Wagner, Lis; K Andersen, Klaus; Minet, Lisbeth R.; Hansen, Olfred. Male coping through a long-term cancer trajectory. Secondary outcomes from a RTC examining the effect of a multidisciplinary rehabilitation program (RePCa) among radiated men with prostate cancer. Acta Oncologica 2017;56(2):254-261. | Wrong intervention, multidisciplinary intervention, primary pelvic floor muscle training |
| 24 | Dorff 2017 | Dorff T.; Gross M.; Quinn D.I.; Pinski J.; Schroeder T.; Groshen S.; DieliConwright C.; Kiwata, J.. Impact of resistance exercise on metabolic syndrome (MetS) parameters in men receiving androgen deprivation therapy (ADT) for prostate cancer. Annals of Oncology 2017; Conference (Journal Article):42n. | An abstract, no usable data |
| 25 | Doyle 2019 | Doyle C.P.; Thirion P.; O'Neill B.; Dunne M.; Curtis, V. P2 RCT of Home-based physical activity in pts treated by ADT and EBRT for localised prostate carcinoma. Radiotherapy and Oncology 2019; Conference: ESTRO 38: Targeting optimal care, together. Italy (Journal Article): date of Publication: Ar 2019. | Wrong intervention, home training no information on supervision |
| 26 | Dunne 2019a | Dunne R.F.; Heckler C.E.; Inglis J.E.; Lin P.J.; Fung C.; Peppone L.J.; Lopez G.; Culakova E.; Kleckner I.; Janelsins M.C.; Jatoi A.; Mohile S.G.; Mustian, K. M. Evaluating the effects of a structured exercise intervention on physical self-worth in men with prostate cancer: Addressing an unmet need. Journal of Clinical Oncology 2019; Conference (Journal Article):2019. | Wrong intervention, an abstract, home-based exercise intervetnion and no information on supervision, no usable data |
| 27 | Dunne 2019b | Dunne R.; Heckler C.; Inglis J.; Lin P.J.; Fung C.; Peppone L.; Lopez G.; Culakova E.; Kleckner I.; Janelsins M.; Jatoi A.; Mohile S.; Mustian, K. Assessing the impact of a structured exercise intervention on physical self-worth in men with prostate cancer: A randomized controlled trial. Supportive Care in Cancer 2019; Conference: 2019 Joint Meeting of the Multinational Association of Supportie Care in Cancer, MASCC and the International Society of Oral Oncology, ISOO. United States (Journal Article): date of Publicaton: June 2019. | Wrong intervention, an abstract, home-based exercise intervetnion and no information on supervision, no usable data |
| 28 | Edmunds 2020 | Edmunds, Kim; Reeves, Penny; Scuffham, Paul; Galvao, Daniel A.; Newton, Robert U.; Jones, Mark; Spry, Nigel; Taaffe, Dennis R.; Joseph, David; Chambers, Suzanne K.; Tuffaha, Haitham. Cost-Effectiveness Analysis of Supervised Exercise Training in Men with Prostate Cancer Previously Treated with Radiation Therapy and Androgen-Deprivation Therapy. Applied Health Economics & Health Policy 2020; (Journal Article). | Wrong patient population, previously treated with ADT |
| 29 | Eriksen 2017 | Eriksen, A. K.; Hansen, R. D.; Larsen, R. G.; Jensen, J. M.; Overgaard, K.; Borre, M.; Kyro, C.; Landberg, R.; Olsen, A.; Tjonneland, A. A lifestyle intervention among elderly men on active surveillance for non-aggressive prostate cancer: a randomised feasibility study with whole-grain rye and exercise. Trials 2017;18 (20):Epub. | Wrong patient population, active surveillance no ADT |
| 30 | Fairman 2019 | Fairman, Ciaran M.; Kendall, Krissy L.; Newton, Robert U.; Hart, Nicolas H.; Taaffe, Dennis R.; Chee, Raphael; Tang, Colin I.; Galvao, Daniel A.. Examining the effects of creatine supplementation in augmenting adaptations to resistance training in patients with prostate cancer undergoing androgen deprivation therapy: a randomised, double-blind, placebo-controlled trial. BMJ Open 2019;9(9):e030080. | Wrong comparator, both groups receives supervised training |
| 31 | Farley 2020 | Farley M.J.; Skinner T.L.; Schaumberg M.A.; Jenkins, D. G. The interplay between inflammatory markers and body composition with 6 months of HIIT in breast, colorectal and prostate cancer survivors. Asia-Pacific Journal of Clinical Oncology.Conference: 47th Annual Scientific Meeting, Quality and Safety, Implementation Science, Cardio-Oncology.Virtual 2020;16(SUPPL 8):203  Blackwell Publishing Ltd 2020 | An abstract for a protocol, no results |
| 32 | Focht 2019 | Focht, Brian C.; Lucas, Alexander R.; Grainger, Elizabeth; Simpson, Christina; Fairman, Ciaran M.; Thomas-Ahner, Jennifer M.; Chaplow, Zachary L.; DeScenza, Victoria R.; Bowman, Jessica; Clinton, Steven K.. Effects of a Group-Mediated Cognitive Behavioral Lifestyle Intervention on Select Social Cognitive Outcomes in Prostate Cancer Patients Undergoing Androgen Deprivation Therapy. Integrative Cancer Therapies 2019;18(Journal Article):1534735419893764. | Wrong outcomes, Focth 2018 already included. |
| 33 | Frawley 2020 | Frawley, Helena C.; Lin, Kuan-Yin; Granger, Catherine L.; Higgins, Rosemary; Butler, Michael; Denehy, Linda. An allied health rehabilitation program for patients following surgery for abdomino-pelvic cancer: a feasibility and pilot clinical study. Supportive Care in Cancer 2020;28 (3):1335-1350. | Wrong study design, Not a RCT and wrong population |
| 34 | Freedland 2019 | Freedland, Stephen J.; Howard, Lauren; Allen, Jenifer; Smith, Jordan; Stout, Jennifer; Aronson, William; Inman, Brant A.; Armstrong, Andrew J.; George, Daniel; Westman, Eric; Lin, Pao-Hwa. A lifestyle intervention of weight loss via a low-carbohydrate diet plus walking to reduce metabolic disturbances caused by androgen deprivation therapy among prostate cancer patients: carbohydrate and prostate study 1 (CAPS1) randomized controlled trial. Prostate Cancer & Prostatic Diseases 2019;22(3):428-437. | Wrong intervention, walking intervention, no information on supervision |
| 35 | Galvao 2014 | Galvao,D. A.; Spry,N.; Denham,J.; Taaffe,D. R.; Cormie,P.; Joseph,D.; Lamb,D. S.; Chambers,S. K.; Newton,R. U.. A multicentre year-long randomised controlled trial of exercise training targeting physical functioning in men with prostate cancer previously treated with androgen suppression and radiation from TROG 03.04 RADAR. European urology 2014;65(5):856-864. [DOI: 10.1016/j.eururo.2013.09.041 | Wrong patient population, previously treated with ADT |
| 36 | Galvao 2016 | Galvao, D. Exercise as a synergistic medicine for prostate cancer. Asia-Pacific Journal of Clinical Oncology 2016; Conference: Annual Scientific Meeting of the Australian and New Zealand Urogenital and Prostate, GU Cancer: Expanding our Horizons, ANZUP 2016. Australia (Journal Article): date of Publication: July 2016. | An abstract for a planned study |
| 37 | Galvao 2017a | Galvao D.A.; Taaffe D.R.; Spry N.; Cormie P.; Joseph D.; Chambers S.K.; PeddleMcintyre C.; Hart N.H.; Denham J.; Baker M.; Newton, R. U.. Efficacy and safety of a modular multi-modal exercise program in prostate cancer patients with bone metastases: A randomized controlled trial. BJU international 2017;Conference(Journal Article):18th. | An abstract with no usable data, full text already included, Galvao 2018 |
| 38 | Galvao 2017b | Galvao, D. Exercise as a synergistic medicine for cancer. Clinical endocrinology 2017; Conference (Journal Article):Enorne. | Wrong study design, an abstract, Not a RCT |
| 39 | Galvao 2018 | Galvao, Daniel A.; Hayne, Dickon; Frydenberg, Mark; Chambers, Suzanne K.; Taaffe, Dennis R.; Spry, Nigel; Scuffham, Paul A.; Ware, Robert S.; Hart, Nicolas H.; Newton, Robert U.. Can exercise delay transition to active therapy in men with low-grade prostate cancer? A multicentre randomised controlled trial. BMJ Open 2018;8(4):e022331. | Wrong patient population, active surveillance no ADT |
| 40 | Galvao 2020 | Galvao, Daniel A.; Taaffe, Dennis R.; Chambers, Suzanne K.; Fairman, Ciaran M.; Spry, Nigel; Joseph, David; Newton, Robert U.  Exercise intervention and sexual function in advanced prostate cancer: a randomised controlled trial.  BMJ supportive & palliative care 2020;(Journal Article):  2020 | Wrong outcome, primary publication (Galvao 2018) already included, no outcomes of interest in this publication |
| 41 | Gaskin 2016 | Gaskin, Cadeyrn J.; Fraser, Steve F.; Owen, Patrick J.; Craike, Melinda; Orellana, Liliana; Livingston, Patricia M.. Fitness outcomes from a randomised controlled trial of exercise training for men with prostate cancer: the ENGAGE study. Journal of Cancer Survivorship 2016;10(6):972-980. | Wrong patient population, only a minority of the patients receive ADT |
| 42 | Gaskin 2017 | Gaskin, Cadeyrn J.; Craike, Melinda; Mohebbi, Mohammadreza; Courneya, Kerry S.; Livingston, Patricia M.. A Clinician Referral and 12-Week Exercise Training Program for Men With Prostate Cancer: Outcomes to 12 Months of the ENGAGE Cluster Randomized Controlled Trial. Journal of Physical Activity & Health 2017;14(5):353-359. | Wrong patient population, only a minority of the patients receive ADT |
| 43 | Gilbert 2016 | Gilbert, Stephen E.; Tew, Garry A.; Fairhurst, Caroline; Bourke, Liam; Saxton, John M.; Winter, Edward M.; Rosario, Derek J. Effects of a lifestyle intervention on endothelial function in men on long-term androgen deprivation therapy for prostate cancer. British journal of cancer 2016;114 (4):401-408. | Wrong outcomes, full text with outcomes of interest already included, Bourke 2014 |
| 44 | Gray 2019 | Gray, Marquita S.; Judd, Suzanne E.; Sloane, Richard; Snyder, Denise C.; Miller, Paige E.; Demark-Wahnefried, Wendy. Rural-urban differences in health behaviors and outcomes among older, overweight, long-term cancer survivors in the RENEW randomized control trial. Cancer Causes & Control 2019;30(4):301-309. | Wrong patient population, cancer patients |
| 45 | Hart 2017 | Hart, Nicolas H.; Newton, Robert U.; Spry, Nigel A.; Taaffe, Dennis R.; Chambers, Suzanne K.; Feeney, Kynan T.; Joseph, David J.; Redfern, Andrew D.; Ferguson, Tom; Galvao, Daniel A.. Can exercise suppress tumour growth in advanced prostate cancer patients with sclerotic bone metastases? A randomised, controlled study protocol examining feasibility, safety and efficacy. BMJ Open 2017;7(5):e014458. | A protocol |
| 46 | Herbert 2012 | Hebert,J. R.; Hurley,T. G.; Harmon,B. E.; Heiney,S.; Hebert,C. J.; Steck,S. E.. A diet, physical activity, and stress reduction intervention in men with rising prostate-specific antigen after treatment for prostate cancer. Cancer epidemiology 2012;36(2):e128-36. | Wrong patient population, excludes patients receiving ADT |
| 47 | Hojan 2015 | Hojan K.; Milecki P.; KwiatkowskaBorowczyk E.; Leporowska, E. Physical exercises, inflammation, fatigue and aerobic fitness in prostate cancer patients undergoing antiandrogen-and radiotherapy. Archives of Physical Medicine and Rehabilitation.Conference: 2015 American Congress of Rehabilitation Medicine Annual Conference, ACRM 2015.Dallas, TX United States.Conference Publication: (var.pagings) 2015; 96 (10):e5. | An abstract, full text already included Hojan 2016 and Hojan 2017 |
| 48 | Hvid 2016 | Hvid,Thine; Lindegaard,Birgitte; Winding,Kamilla; Iversen,Peter; Brasso,Klaus; Solomon,Thomas P. J.; Pedersen,Bente K.; Hojman,Pernille. Effect of a 2-year home-based endurance training intervention on physiological function and PSA doubling time in prostate cancer patients. Cancer Causes & Control 2016;27(2):165-174Netherlands 2016 | Wrong patient population, patients following radical prostatectomy or patients on active surveillance |
| 49 | Jones 2014 | Jones,L. W.; Hornsby,W. E.; Freedland,S. J.; Lane,A.; West,M. J.; Moul,J. W.; Ferrandino,M. N.; Allen,J. D.; Kenjale,A. A.; Thomas,S. M.; Herndon,J. E.,2nd; Koontz,B. F.; Chan,J. M.; Khouri,M. G.; Douglas,P. S.; Eves,N. D. Effects of nonlinear aerobic training on erectile dysfunction and cardiovascular function following radical prostatectomy for clinically localized prostate cancer. European urology 2014;65 (5):852-855. | Wrong patient population, patients following radical prostatectomy, No ADT |
| 50 | Kenfield 2018 | Kenfield S.A.; Blarigan E.V.; Ameli N.; Lavaki E.; Monroy C.; Tantum L.K.; Newton R.U.; Zhang L.; Cooperberg M.R.; Carroll P.; Chan, J. M.. Prostate 8 study: A pilot randomized controlled trial (RCT) of a web-based lifestyle intervention versus control group among men with prostate cancer. Journal of Clinical Oncology 2018Conference: 2018 Genitourinary Cancers Symposium. United States (Journal Article): date of Publication: February 2018. | An abstract, wrong intervention, web-based lifestyle intervention |
| 51 | Kim 2018 | Kim, Soo Hyun; Seong, Do Hwan; Yoon, Sang Min; Choi, Young Deuk; Choi, Eunju; Song, Youngkyu; Song, Hosook. The Effect on Bone Outcomes of Home-based Exercise Intervention for Prostate Cancer Survivors Receiving Androgen Deprivation Therapy: A Pilot Randomized Controlled Trial. Cancer nursing 2018;41(5):379-388. | Wrong intervention, Home-based training with telephone counselling according to Banduras theory of self-efficacy, no supervision according to our definition of supervision were the exercise should be instructed, supervised, and monitored by a health care professional |
| 52 | Kim 2020 | Kim S.H.; Joung J.Y.; Lee K.H.; Song M.K.; Yoo J.S.; Chung, S. H.  A randomized control study of a prospective questionnaire analysis about the efficacy of smart health after-care including the physical muscle enforced function test in patients with advanced or metastatic prostate cancer who underwent an androgen deprivation test after primary curative therapy.  Journal of Urology 2020;Conference: 2020 Annual Meeting of the American Urological Association. Washington, DC United States(Journal Article):ate of Pubaton: Ar 2020  Lippincott Williams and Wilkins 2020 | Wrong comparator, both groups received exercise therapy |
| 53 | Kiwata 2017 | Kiwata J.L.; Dorff T.B.; Schroeder E.T.; DieliConwright, C. M. Effect of a supervised exercise intervention on sarcopenic obesity and metabolic syndrome in prostate cancer patients: A randomized pilot study. Cancer research 2017; Conference (Journal Article):Ameran. | An abstract, full-text available, Dawson 2018 |
| 54 | Lam 2017 | Lam T.; Cheema B.; Hayden A.; Gurney H.; Gounden S.; Reddy N.; Stone G.; McLean M.; Birznience, V.. ADT in prostate cancer patients: Prevention of adverse effects using a 6-month home-based progressive resistance training program. Clinical endocrinology 2017; Conference (Journal Article):Enorne. | Wrong intervention, home-based training with telephone support, no supervision according to our definition of supervision |
| 55 | Lam 2019 | Lam, Teresa; McLean, Mark; Hayden, Amy; Poljak, Anne; Cheema, Birinder; Gurney, Howard; Stone, Glenn; Bahl, Neha; Reddy, Navneeta; Shahidipour, Haleh; Birzniece, Vita. A potent liver-mediated mechanism for loss of muscle mass during androgen deprivation therapy. Endocrine Connections 2019; 8 (5):605-615. | Wrong intervention, home-based training with telephone support, no supervision according to our definition of supervision |
| 56 | Lam 2020 | Lam, Teresa; Cheema, Birinder; Hayden, Amy; Lord, Stephen R.; Gurney, Howard; Gounden, Shivanjini; Reddy, Navneeta; Shahidipour, Haleh; Read, Scott; Stone, Glenn; McLean, Mark; Birzniece, Vita  Androgen deprivation in prostate cancer: benefits of home-based resistance training.  Sports Medicine - Open 2020;6(1):59  2020 | Wrong intervention, minimal supervision, 2 times in the first week, hereafter only supervision every 12^th^ week. |
| 57 | Livingston 2015 | Livingston, Patricia M.; Craike, Melinda J.; Salmon, Jo; Courneya, Kerry S.; Gaskin, Cadeyrn J.; Fraser, Steve F.; Mohebbi, Mohammadreza; Broadbent, Suzanne; Botti, Mari; Kent, Bridie; ENGAGE Uro-Oncology Clinicians' Group. Effects of a clinician referral and exercise program for men who have completed active treatment for prostate cancer: A multicenter cluster randomized controlled trial (ENGAGE). Cancer 2015; 121 (15):2646-2654. | Wrong patient population, only a minority of the patients receive ADT |
| 58 | Lyons 2016 | Lyons, Karen S.; Winters-Stone, Kerri M.; Bennett, Jill A.; Beer, Tomasz M. The effects of partnered exercise on physical intimacy in couples coping with prostate cancer.. Health Psychology 2016; 35 (5):509-513. | Wrong intervention, couple intervention (patient and partner) |
| 59 | Mardani 2021 | Mardani, Abbas; Pedram Razi, Shadan; Mazaheri, Reza; Haghani, Shima; Vaismoradi, Mojtaba  Effect of the exercise programme on the quality of life of prostate cancer survivors: A randomized controlled trial.  International journal of nursing practice 2021;27(2):e12883  2021 | Wrong intervention, no information of supervision |
| 60 | Mareschal 2015 | Mareschal J.; Frambati L.; Biason E.; PhilippParadisi S.; Rigoli P.; Weber K.; Zilli T.; Miralbell R.; Pichard, C.. Prostate cancer and androgen deprivation in frail patients: A 2YR prospective multidisciplinary program. Preliminary data. Supportive Care in Cancer.Conference: 2015 International MASCC/ISOO Symposium: Supportive Care in Cancer. Copenhagen Denmark. Conference Publication: (var.pagings) 2015; 23 (1 SUPPL. 1):S277. | Wrong study design, an abstract, Not an RCT, no control group |
| 61 | Martin 2015 | Martin, Eric A.; Battaglini, Claudio L.; Hands, Beth; Naumann, Fiona. Higher-Intensity Exercise Results in More Sustainable Improvements for VO2peak for Breast and Prostate Cancer Survivors. Oncology nursing forum 2015;42(3):241-249. | Wrong patient population, only a minority receive ADT |
| 62 | Martin 2016 | Martin, Eric; Battaglini, Claudio; Hands, Beth; Naumann, Fiona L. Higher-intensity exercise helps cancer survivors remain motivated. Journal of Cancer Survivorship 2016;10 (3):524-533. | Wrong patient population, breast and prostate cancer |
| 63 | McGowan 2013 | McGowan,E. L.; North,S.; Courneya,K. S.. Randomized controlled trial of a behavior change intervention to increase physical activity and quality of life in prostate cancer survivors. Annals of Behavioral Medicine: A Publication of the Society of Behavioral Medicine 2013; 46(3):382-393. | Wrong intervention, a behavior change intervention |
| 64 | Monga 2007 | Monga,U.; Garber,S. L.; Thornby,J.; Vallbona,C.; Kerrigan,A. J.; Monga,T. N.; Zimmermann,K. P. Exercise prevents fatigue and improves quality of life in prostate cancer patients undergoing radiotherapy. Archives of Physical Medicine and Rehabilitation 2007; 88(11):1416-1422. | Wrong patient population, patients with localized prostate cancer undergoing radiotherapy |
| 65 | Mustian 2015 | Mustian K.; Janelsins M.; Peppone L.; Kamen C.; Heckler, C. Exercise effects on muscular strength, cancer-related fatigue, and mitochondrial and nuclear gene expression in skeletal muscle among older prostate cancer patients.. Supportive Care in Cancer.Conference: 2015 International MASCC/ISOO Symposium: Supportive Care in Cancer.Copenhagen Denmark.Conference Publication: (var.pagings) 2015; 23(1 SUPPL. 1):S89-S90. | Wrong population, wrong intervention, only 6 weeks training, an abstract, no usable data, 53% receive ADT |
| 66 | Newton 2009 | Newton,Robert U.; Taaffe,Dennis R.; Spry,Nigel; Gardiner,Robert A.; Levin,Gregory; Wall,Bradley; Joseph,David; Chambers,Suzanne K.; Galvao, Daniel A. A phase III clinical trial of exercise modalities on treatment side-effects in men receiving therapy for prostate cancer. BMC Cancer 2009; 9(Journal Article):210 England 2009. | A protocol |
| 67 | Newton 2012 | Newton,Robert U.; Taaffe,Dennis R.; Spry,Nigel; Cormie,Prue; Chambers,Suzanne K.; Gardiner,Robert A.; Shum,David Hk; Joseph,David; Galvao,Daniel A.  Can exercise ameliorate treatment toxicity during the initial phase of testosterone deprivation in prostate cancer patients? Is this more effective than delayed rehabilitation?  BMC Cancer 2012;12(Journal Article):432  England 2012 | A protocol |
| 68 | Newton 2013 | Newton R.; Cormie P.; Galvao D.; Spry N.; Joseph D. Can exercise prevent treatment toxicity in prostate cancer patients initiating androgen suppression therapy: A randomised controlled trial.BJU international 2013;112(Web Page):63  Blackwell Publishing Ltd 2013 | An abstract, full text already included Cormie 2015 |
| 69 | Newton 2017 | Newton R.U.; Galvao D.A.; Spry N.; Joseph D.; Chambers S.K.; Gardiner R.A.; Hayne D.; Hart N.H.; Wall B.A.; Bolam K.A.; Taaffe, D. R.. Exercise medicine to arrest bone loss in men with prostate cancer undergoing androgen deprivation therapy: A 12-month randomized controlled trial. BJU international 2017;Conference(Journal Article):18th. | An abstract, full text already included, Taaffe 2017 |
| 70 | Newton 2018 | Newton, Robert U.; Kenfield, Stacey A.; Hart, Nicolas H.; Chan, June M.; Courneya, Kerry S.; Catto, James; Finn, Stephen P.; Greenwood, Rosemary; Hughes, Daniel C.; Mucci, Lorelei; Plymate, Stephen R.; Praet, Stephan F. E.; Guinan, Emer M.; Van Blarigan, Erin L.; Casey, Orla; Buzza, Mark; Gledhill, Sam; Zhang, Li; Galvao, Daniel A.; Ryan, Charles J.; Saad, Fred. Intense Exercise for Survival among Men with Metastatic Castrate-Resistant Prostate Cancer (INTERVAL-GAP4): a multicentre, randomised, controlled phase III study protocol. BMJ Open 2018;8(5):e022899. | A protocol |
| 71 | Newton 2019 | Newton, Robert U.; Christophersen, Claus T.; Fairman, Ciaran M.; Hart, Nicolas H.; Taaffe, Dennis R.; Broadhurst, David; Devine, Amanda; Chee, Raphael; Tang, Colin I.; Spry, Nigel; Galvao, Daniel A. Does exercise impact gut microbiota composition in men receiving androgen deprivation therapy for prostate cancer? A single-blinded, two-armed, randomised controlled trial. BMJ Open 2019; 9 (4):e024872. | A protocol |
| 72 | Newton 2021 | Newton, Robert U.; Mavropalias, Georgios; Fragala, Maren S.; Kraemer, William J.; Hakkinen, Keijo; Taaffe, Dennis R.; Spry, Nigel; Joseph, David; Galvao, Daniel A.  Radiotherapy before or during androgen-deprivation therapy does not blunt the exercise-induced body composition protective effects in prostate cancer patients: A secondary analysis of two randomized controlled trials.  Experimental gerontology 2021;151(Journal Article):111427  2021 | Wrong outcomes, secondary analyses of two trials already included (Cormie 2015, Taaffe 2019), no outcomes of interest in this publication |
| 73 | Nilsen 2016a | Nilsen T.S.; Thorsen L.; Kirkegaard C.; Ugelstad I.; Fossa S.D.; Raastad, T.. The effect of strength training on muscle cellular stress in prostate cancer patients on ADT. Endocrine Connections 2016;5(2):74-82. | Wrong outcome, primary publication (Nielsen 2015) already included, no outcomes of interest in this publication |
| 74 | Nilsen 2016b | Nilsen, T. S.; Thorsen, L.; Fossa, S. D.; Wiig, M.; Kirkegaard, C.; Skovlund, E.; Benestad, H. B.; Raastad, T. Effects of strength training on muscle cellular outcomes in prostate cancer patients on androgen deprivation therapy. Scandinavian Journal of Medicine & Science in Sports 2016; 26 (9):1026-1035. | Wrong outcome, primary publication (Nielsen 2015) already included, no outcomes of interest in this publication |
| 75 | Nilsen 2018 | Nilsen, Tormod S.; Scott, Jessica M.; Michalski, Meghan; Capaci, Catherine; Thomas, Samantha; Herndon, James E. 2nd; Sasso, John; Eves, Neil D.; Jones, Lee W.. Novel Methods for Reporting of Exercise Dose and Adherence: An Exploratory Analysis. Medicine & Science in Sports & Exercise 2018;50(6):1134-1141. | Wrong patient population patients following radical prostatectomy |
| 76 | Norris 2015 | Norris, M. K.; Bell, G. J.; North, S.; Courneya, K. S. Effects of resistance training frequency on physical functioning and quality of life in prostate cancer survivors: a pilot randomized controlled trial. Prostate Cancer & Prostatic Diseases 2015; 18 (3):281-287. | Wrong comparator, comparison of two different training programs (training 2 days a week vs 3 days a week) |
| 77 | O'Neill 2015 | O'Neill, Roisin F.; Haseen, Farhana; Murray, Liam J.; O'Sullivan, Joe M.; Cantwell, Marie M.. A randomised controlled trial to evaluate the efficacy of a 6-month dietary and physical activity intervention for patients receiving androgen deprivation therapy for prostate cancer. Journal of Cancer Survivorship 2015; 9 (3):431-440. | Wrong intervention, recommendations of daily walking, no supervision |
| 78 | O'Neill 2019 | Oneill M.; Santa Mina D.; Sabiston C.; Tomlinson G.; Alibhai, S. M. Benefits of physical activity on reducing cancer-related fatigue for older men with prostate cancer: a randomized controlled trial. Journal of Geriatric Oncology 2019; Conference (Journal Article):19th. | Wrong intervention, no supervised training |
| 79 | Owen 2017 | Owen, Patrick J.; Daly, Robin M.; Livingston, Patricia M.; Mundell, Niamh L.; Dalla Via, Jack; Millar, Jeremy L.; Fraser, Steve F.. Efficacy of a multi-component exercise programme and nutritional supplementation on musculoskeletal health in men treated with androgen deprivation therapy for prostate cancer (IMPACT): study protocol of a randomised controlled trial. Trials [Electronic Resource] 2017; 18 (1):451. | A protocol |
| 80 | Papadopoulos 2020 | Papadopoulos, Efthymios; Mina, Daniel Santa; Culos-Reed, Nicole; Durbano, Sara; Ritvo, Paul; Sabiston, Catherine M.; Krahn, Murray; Tomlinson, George; O'Neill, Meagan; Iqbal, Amna; Timilshina, Narhari; Matthew, Andrew; Warde, Padraig; Alibhai, Shabbir M. H.Effects of six months of aerobic and resistance training on metabolic markers and bone mineral density in older men on androgen deprivation therapy for prostate cancer. Journal of Geriatric Oncology 2020; 11(7):1074-1077 (Journal Article). | Wrong comparator, three groups, all groups receive supervision |
| 81 | Park 2012 | Park,S. W.; Kim,T. N.; Nam,J. K.; Ha,H. K.; Shin,D. G.; Lee,W.; Kim,M. S.; Chung,M. K.. Recovery of overall exercise ability, quality of life, and continence after 12-week combined exercise intervention in elderly patients who underwent radical prostatectomy: a randomized controlled study. Urology 2012;80(2):299-305. [DOI: 10.1016/j.urology.2011.12.060 | Wrong patient population, patients receiving radical prostatectomy |
| 82 | Park 2018 | Park Y.H.; Lee J.I.; Lee J.Y.; Cheong I.Y.; Hwang J.H.; Seo S.I.; Lee K.H.; Yoo J.S.; Chung S.H.; Ko Y.S.; Lee Y.H.; Lee, S. I. Internet- and mobile-based lifestyle intervention for prostate cancer patients on androgen deprivation therapy: Prospective, multicenter, randomized trial. Journal of Clinical Oncology 2018; Conference (Journal Article):2018. | Wrong intervention, a mobile application and a wearble device providing general health information, no supervised training |
| 83 | Pernar 2017 | Pernar, Claire H.; Fall, Katja; Rider, Jennifer R.; Markt, Sarah C.; Adami, Hans-Olov; Andersson, Sven-Olof; Valdimarsdottir, Unnur; Andren, Ove; Mucci, Lorelei A. A Walking Intervention Among Men With Prostate Cancer: A Pilot Study. Clinical Genitourinary Cancer 2017; 15 (6):e1021-e1028. | Wrong intervention, intervention was participation in a walking group, no supervised training |
| 84 | Piraux 2021 | Piraux, Elise; Caty, Gilles; Renard, Laurette; Vancraeynest, David; Tombal, Bertrand; Geets, Xavier; Reychler, Gregory  Effects of high-intensity interval training compared with resistance training in prostate cancer patients undergoing radiotherapy: a randomized controlled trial.  Prostate Cancer & Prostatic Diseases 2021;24(1):156-165  2021 | Wrong patient population |
| 85 | Saad 2016 | Saad F.; Kenfield S.A.; Chan J.M.; Hart N.H.; Courneya K.S.; Catto J.; Finn S.P.; Greenwood R.; Hughes D.C.; Mucci L.A.; Plymate S.R.; Pollak M.N.; Praet S.F.E.; Russell A.P.; Guinan E.M.; Van Blarigan E.; Casey O.; Buzza M.; Ryan C.J.; Newton, R. U. Intense exercise for survival among men with metastatic castrate-resistant prostate cancer (INTERVAL - MCRPC): A Movember funded multicenter, randomized, controlled phase III study. Journal of Clinical Oncology 2016; Conference (Journal Article):2016. | An abstract of a protocol |
| 86 | Sajid 2016 | Sajid, Saleha; Dale, William; Mustian, Karen; Kotwal, Ashwin; Heckler, Charles; Porto, Michelle; Fung, Chunkit; Mohile, Supriya G.. Novel physical activity interventions for older patients with prostate cancer on hormone therapy: A pilot randomized study. Journal of Geriatric Oncology 2016; 7 (2):71-80. | Wrong intervention, no supervised training |
| 87 | Schega 2015 | Schega, Lutz; Torpel, Alexander; Hein, Nico; Napiontek, Andre; Wenzel, Constanze; Becker, Tim. Evaluation of a supervised multi-modal physical exercise program for prostate cancer survivors in the rehabilitation phase: Rationale and study protocol of the ProCaLife study. Contemporary Clinical Trials 2015;45(Pt B):311-319. | A protocol, wrong study design, not a RCT-design, the control group consists of those not wanting the intervention |
| 88 | Sheill 2017 | Sheill, Grainne; Brady, Lauren; Guinan, Emer; Hayes, Brian; Casey, Orla; Greene, John; Vlajnic, Tatjana; Cahill, Fidelma; Van Hemelrijck, Mieke; Peat, Nicola; Rudman, Sarah; Hussey, Juliette; Cunningham, Moya; Grogan, Liam; Lynch, Thomas; Manecksha, Rustom P.; McCaffrey, John; Mucci, Lorelei; Sheils, Orla; O'Leary, John; O'Donnell, Dearbhaile M.; McDermott, Ray; Finn, Stephen. The ExPeCT (Examining Exercise, Prostate Cancer and Circulating Tumour Cells) trial: study protocol for a randomised controlled trial. Trials [Electronic Resource] 2017; 18 (1):456. | A protocol |
| 89 | Sheill 2019 | Sheill G.; Brady L.; Guinan E.M.; Hussey J.M.; Hayes B.; Baird A.M.; Stanfill B.; Casey O.; Murphy V.; Rudman S.M.; Peat N.; Sheils O.; Cahill F.; Van Hemelrijck M.; McCaffrey J.; aO'Donnell D.M.; Mucci L.; Grogan W.; McDermott R.; Finn, S. P. A randomized trial of exercise on quality of life in men with metastatic prostate cancer: The ExPeCT Trial. Journal of Clinical Oncology 2019; Conference: 2019 Supportie Care in Oncology Symposium. United States (Journal Article): date of Publication: November 2019. | An abstract, no usable data |
| 90 | Singh 2017 | Singh F.; Newton R.U.; Taaffe D.R.; Spry N.; Joseph D.; Chambers S.K.; Gardiner R.A.; Galvao, D. A.. Year-long study evaluating the effect of exercise on quality of life and psychological distress in men with prostate cancer undergoing androgen deprivation therapy. BJU international 2017;Conference(Journal Article):18th. | An abstract, full text already included, Taaffe 2017 |
| 91 | Storck 2020 | Storck L.J.; Ruehlin M.; Gaeumann S.; Gisi D.; Schmocker M.; Meffert P.J.; Imoberdorf R.; Pless M.; Ballmer, P. E.  Effect of a leucine-rich supplement in combination with nutrition and physical exercise in advanced cancer patients: A randomized controlled intervention trial.  Clinical Nutrition 2020;39(12):3637-3644  Churchill Livingstone 2020 | Wrong patient population |
| 92 | Taaffe 2018 | Taaffe, Dennis R.; Buffart, Laurien M.; Newton, Robert U.; Spry, Nigel; Denham, James; Joseph, David; Lamb, David; Chambers, Suzanne K.; Galvao, Daniel A.. Time on androgen deprivation therapy and adaptations to exercise: secondary analysis from a 12-month randomized controlled trial in men with prostate cancer. BJU international 2018;121(2):194-202. | Wrong patient population, previously treated with ADT |
| 93 | Teleni 2015 | Teleni L.; Chan R.; Chan A.; Isenring E.A.; Vela I.; Inder W.J.; McCarthy, A. L. Dietary and exercise interventions to improve quality oflife, metabolic risk factors and androgen deficiency symptoms in men with prostate cancer undergoing androgen deprivation therapy. Supportive Care in Cancer.Conference: 2015 International MASCC/ISOO Symposium: Supportive Care in Cancer.Copenhagen Denmark.Conference Publication: (var.pagings) 2015; 23 (1 SUPPL. 1):S171. | Wrong study design, abstract for a systematic review |
| 94 | Trinth 2021 | Trinh, Linda; Kramer, Arthur F.; Rowland, Kendrith; Strom, Dominick A.; Wong, Jaime N.; McAuley, Edward  A pilot feasibility randomized controlled trial adding behavioral counseling to supervised physical activity in prostate cancer survivors: behavior change in prostate cancer survivors trial (BOOST).  Journal of Behavioral Medicine 2021;44(2):172-18604 2021 | Wrong patient population |
| 95 | Uth 2016a | Uth, Jacob; Hornstrup, Therese; Christensen, Jesper F.; Christensen, Karl B.; Jorgensen, Niklas R.; Helge, Eva W.; Schmidt, Jakob F.; Brasso, Klaus; Helge, Jorn W.; Jakobsen, Markus D.; Andersen, Lars L.; Rorth, Mikael; Midtgaard, Julie; Krustrup, Peter. Efficacy of recreational football on bone health, body composition, and physical functioning in men with prostate cancer undergoing androgen deprivation therapy: 32-week follow-up of the FC prostate randomised controlled trial. Osteoporosis International 2016;27(4):1507-1518. | Wrong outcome, primary publication (Uth 2014) already included, no outcomes of interest in this publication |
| 96 | Uth 2016b | Uth, Jacob; Hornstrup, Therese; Christensen, Jesper F.; Christensen, Karl B.; Jorgensen, Niklas R.; Helge, Eva W.; Schmidt, Jakob F.; Brasso, Klaus; Helge, Jorn W.; Jakobsen, Markus D.; Andersen, Lars L.; Rorth, Mikael; Midtgaard, Julie; Krustrup, Peter. Football training in men with prostate cancer undergoing androgen deprivation therapy: activity profile and short-term skeletal and postural balance adaptations. European journal of applied physiology 2016;116(3):471-480. | Wrong outcome, primary publication (Uth 2014) already included, no outcomes of interest in this publication |
| 97 | Uth 2018 | Uth, J.; Fristrup, B.; Haahr, R. D.; Brasso, K.; Helge, J. W.; Rorth, M.; Midtgaard, J.; Helge, E. W.; Krustrup, P.. Football training over 5 years is associated with preserved femoral bone mineral density in men with prostate cancer. Scandinavian Journal of Medicine & Science in Sports 2018;28 (Suppl 1):61-73. | Wrong outcome, primary publication (Uth 2014) already included, no outcomes of interest in this publication |
| 98 | Villumsen 2019 | Villumsen B.R.; Jorgensen M.G.; Frystyk J.; Hordam B.; Borre, M. Home-based 'exergaming' was safe and significantly improved 6-min walking distance in patients with prostate cancer: a single-blinded randomised controlled trial. BJU international 2019;124 (4):600-608. | Wrong intervention, home-based training, no supervision |
| 99 | Westley 2019 | Westley, R. L.; Alexander, S.; D'aquino, A.; Murray, J.; Dearnaley, D. Exploring the Value of a Pre-trial Outlining Exercise in the POPS Trial, which Evaluated the Localising Device ProSpare in Prostate Bed Radiotherapy. Clinical oncology 2019; 31 (2):e25-e25. | Wrong intervention, intervention was radiotherapy, both groups received exercise before the intervention, an abstract |
| 100 | Wilson 2017 | Wilson R.L.; Newton R.U.; Galvao D.A.; Spry N.; Singh F.; Joseph D.; Chambers S.K.; Gardiner R.A.; Wall B.A.; Taaffe, D. R. Contrasting exercise modes enhance muscle strength and physical function in prostate cancer survivors undertaking androgen deprivation therapy: A 12-month randomized controlled trial. BJU international 2017; Conference (Journal Article):18th. | An abstract, full text already included, Taaffe 2017 |
| 101 | Windsor 2004 | Windsor,P. M.; Nicol,K. F.; Potter,J.. A randomized, controlled trial of aerobic exercise for treatment-related fatigue in men receiving radical external beam radiotherapy for localized prostate carcinoma. Cancer 2004;101(3):550-557. | Wrong patient population, patient receiving radiotherapy |
| 102 | Winters-Stone 2015 | Winters-Stone, Kerri M.; Dieckmann, Nathan; Maddalozzo, Gianni F.; Bennett, Jill A.; Ryan, Christopher W.; Beer, Tomasz M.. Resistance Exercise Reduces Body Fat and Insulin During Androgen-Deprivation Therapy for Prostate Cancer. Oncology nursing forum 2015; 42 (4):348-356. | Wrong outcome, primary publication (Winters-Stone 2015) already included, no outcomes of interest in this publication |
| 103 | Winters-Stone 2016 | Winters-Stone, Kerri M.; Lyons, Karen S.; Dobek, Jessica; Dieckmann, Nathan F.; Bennett, Jill A.; Nail, Lillian; Beer, Tomasz M. Benefits of partnered strength training for prostate cancer survivors and spouses: results from a randomized controlled trial of the Exercising Together project.. Journal of Cancer Survivorship 2016;10 (4):633-644. | Wrong intervention, couple intervention (patient and partner) |
| 104 | Zopf 2015 | Zopf, Eva M.; Bloch, Wilhelm; Machtens, Stefan; Zumbe, Jurgen; Rubben, Herbert; Marschner, Stefan; Kleinhorst, Christian; Schulte-Frei, Birgit; Herich, Lena; Felsch, Moritz; Predel, Hans-Georg; Braun, Moritz; Baumann, Freerk T. Effects of a 15-Month Supervised Exercise Program on Physical and Psychological Outcomes in Prostate Cancer Patients Following Prostatectomy: The ProRehab Study.. Integrative Cancer Therapies 2015;14(5):409-418. | Wrong patient population, Prostate Cancer Patients Following Prostatectomy, Patients were excluded if they were scheduled to ADT |

**Supplementary Table S2 AMSTAR assessment of the included systematic review**

**
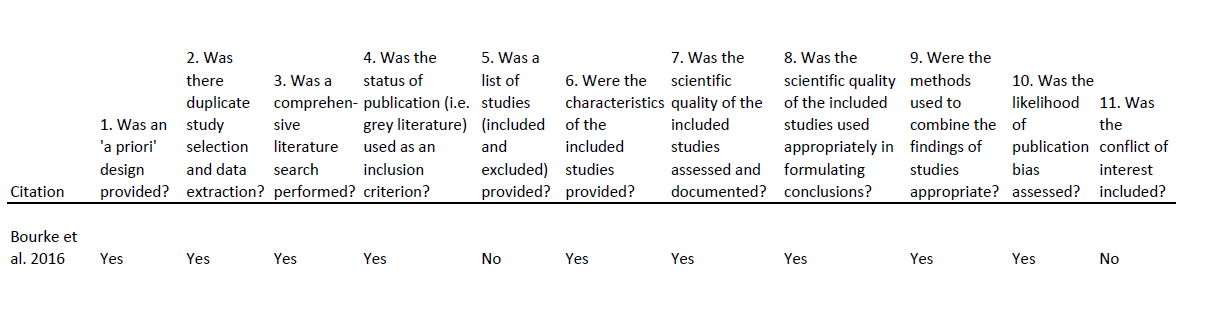
**

**Supplementary Figures S3 Forrest plots for critical and important outcomes.**

Forest plots for critical and important outcomes from the included trials. Risk of bias assessment. Green (+): indicates low risk of bias, red (-): indicates high risk of bias, yellow (?): indicates unclear risk of bias.

Fig S3a. Diagnose-specific quality of life. Supervised exercise therapy vs no exercise


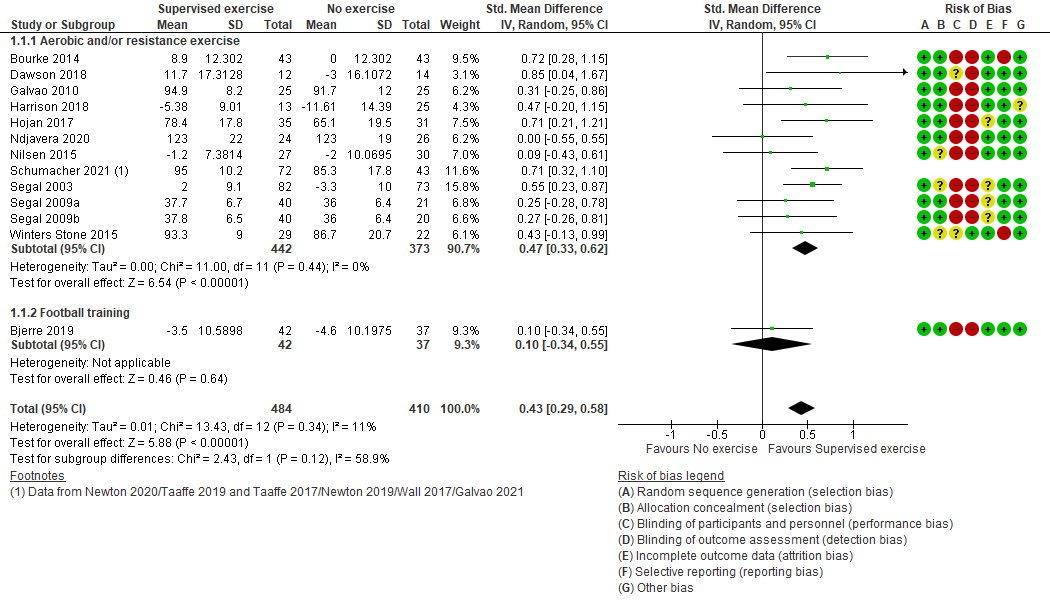


Fig S3b. Health related quality of life, SF-36 mental component. Supervised exercise therapy vs no exercise


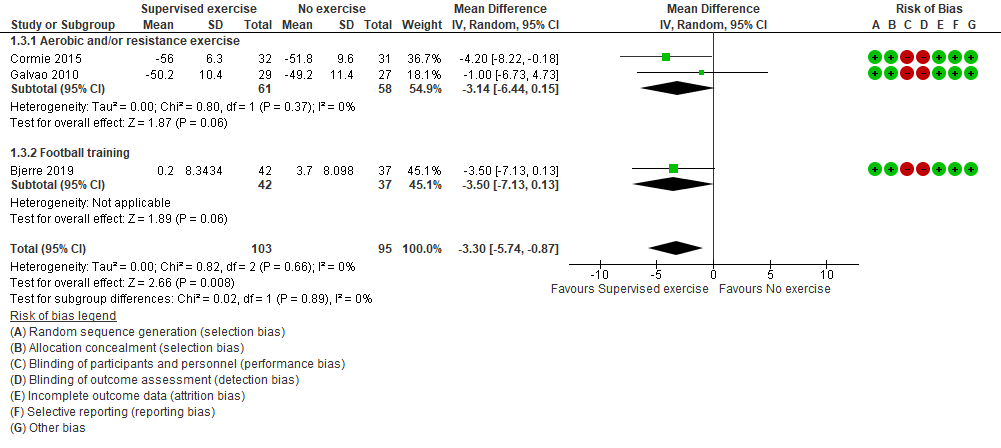


Fig S3c. Health related quality of life, SF-36 physical component. Supervised exercise therapy vs no exercise


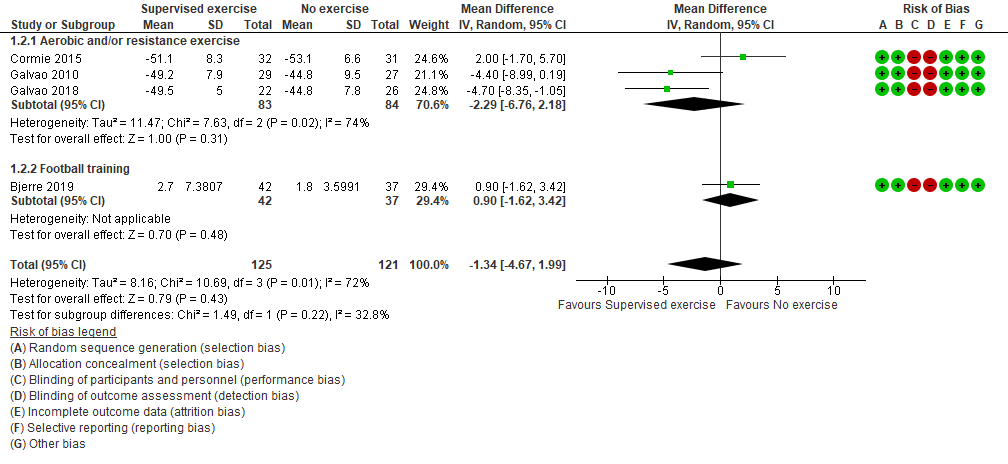


Fig. S3d. Physical performance, measured by walking performance. Supervised exercise therapy vs no exercise


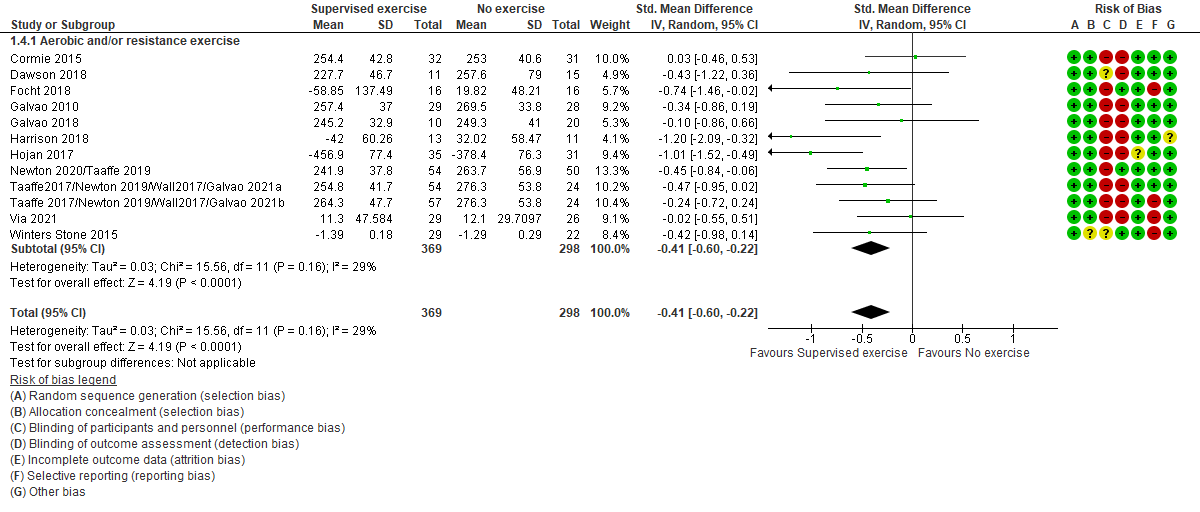


Fig. S3e. Physical performance, sit to stand, performance. Supervised exercise therapy vs no exercise


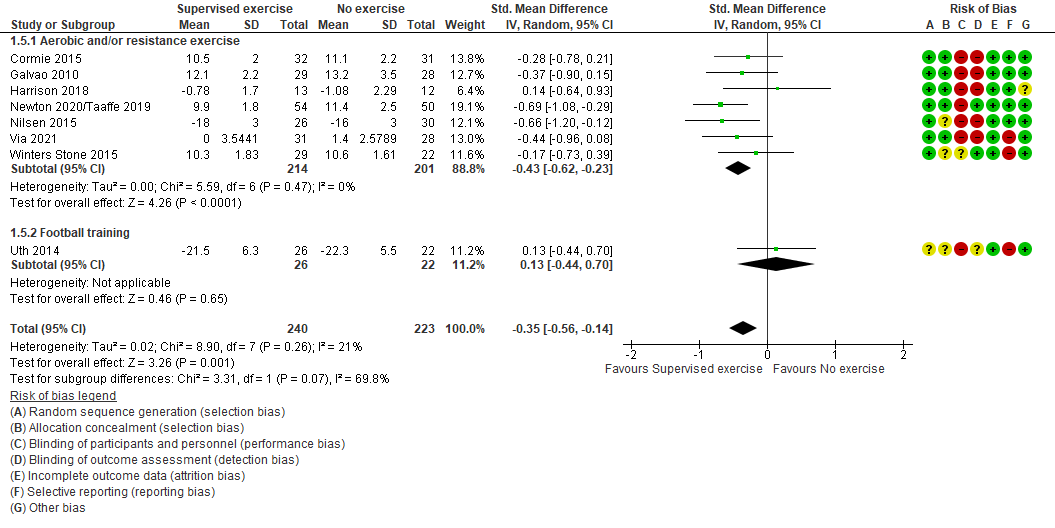


Fig. S3f. Muscle strength. Supervised exercise therapy vs no exercise


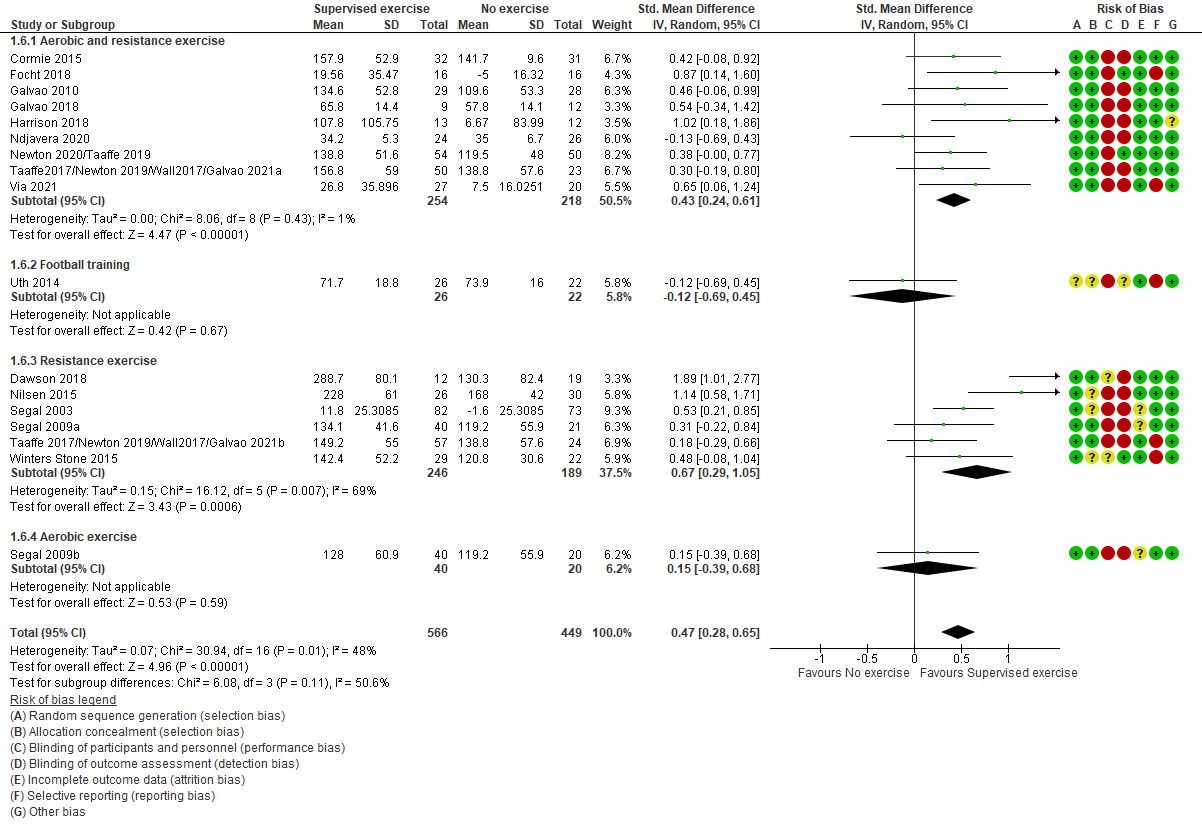


Fig. S3g. Vo2 peak. Supervised exercise therapy vs no exercise


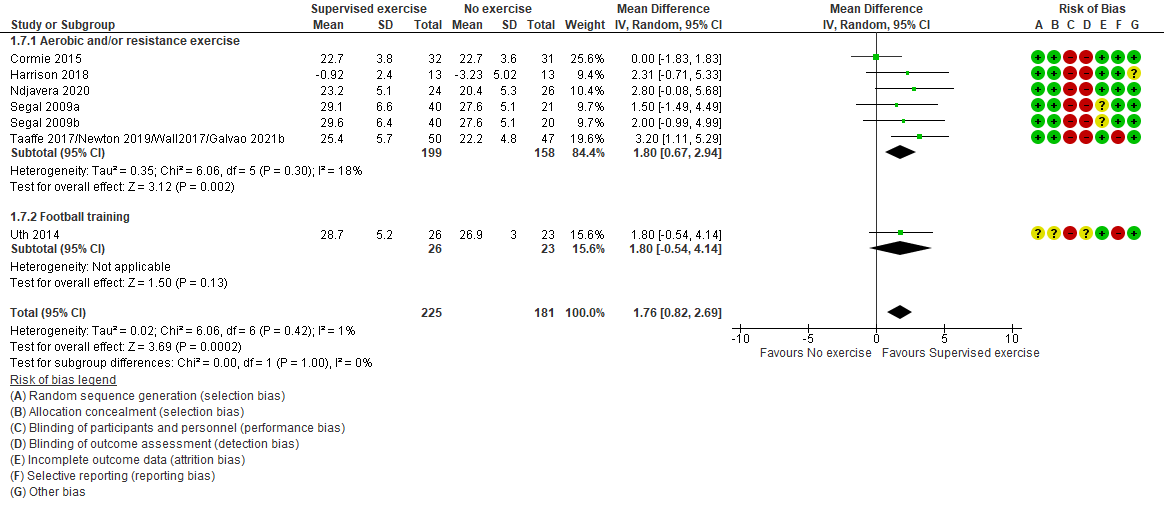


Fig. S3h. Depressive symptoms. Supervised exercise therapy vs no exercise, mean difference BSI-18


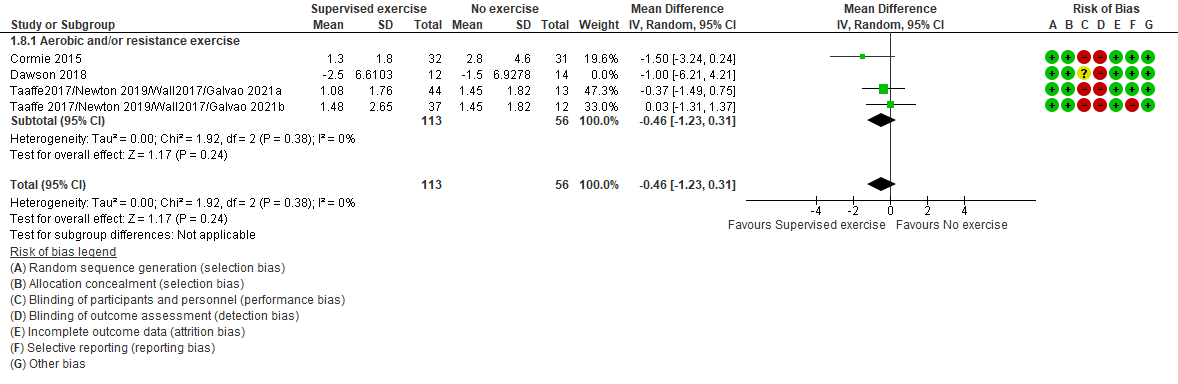


Fig. S3i. Depressive symptoms. Supervised exercise therapy vs no exercise, SMD


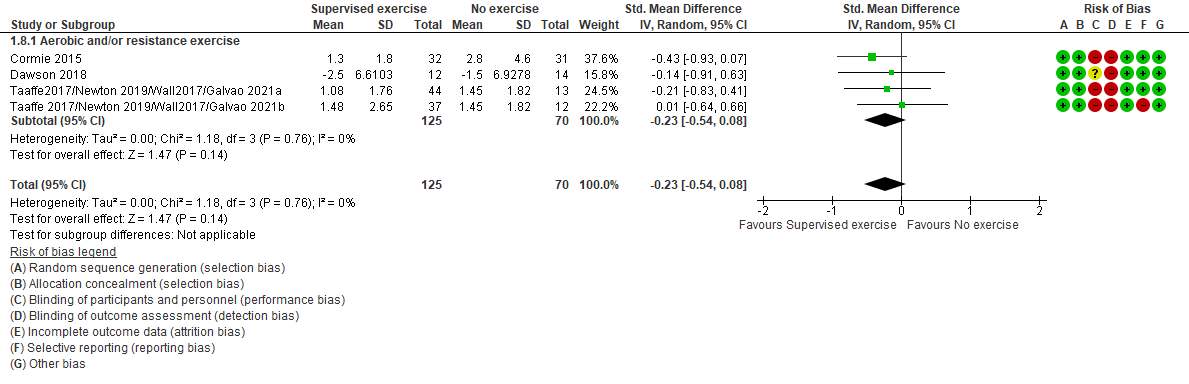


Fig. S3j. Fractures, number of patients with a fracture. Supervised exercise therapy vs no exercise. Risk difference analysis.


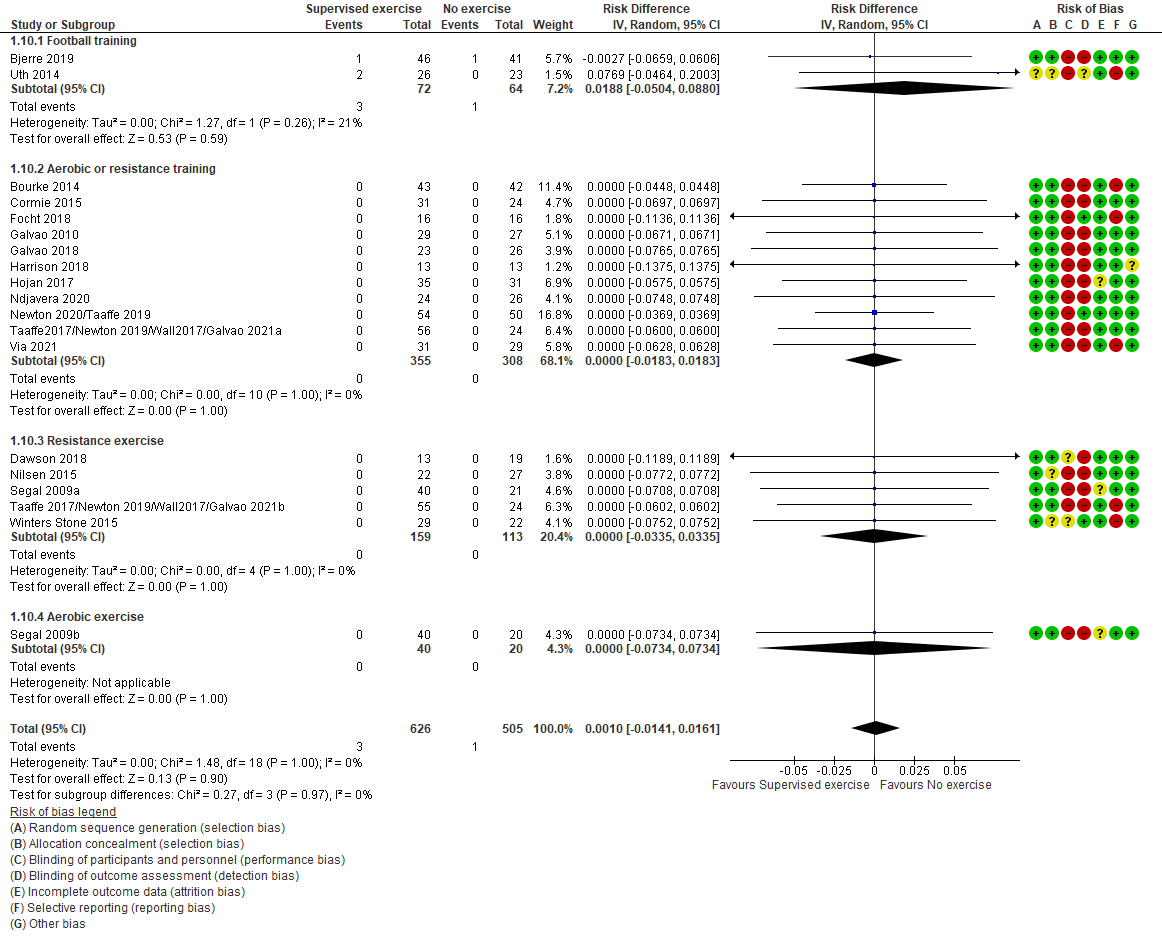


Fig. S3k. Fractures, number of patients with a fracture. Supervised exercise therapy vs no exercise. Risk ratio analysis.


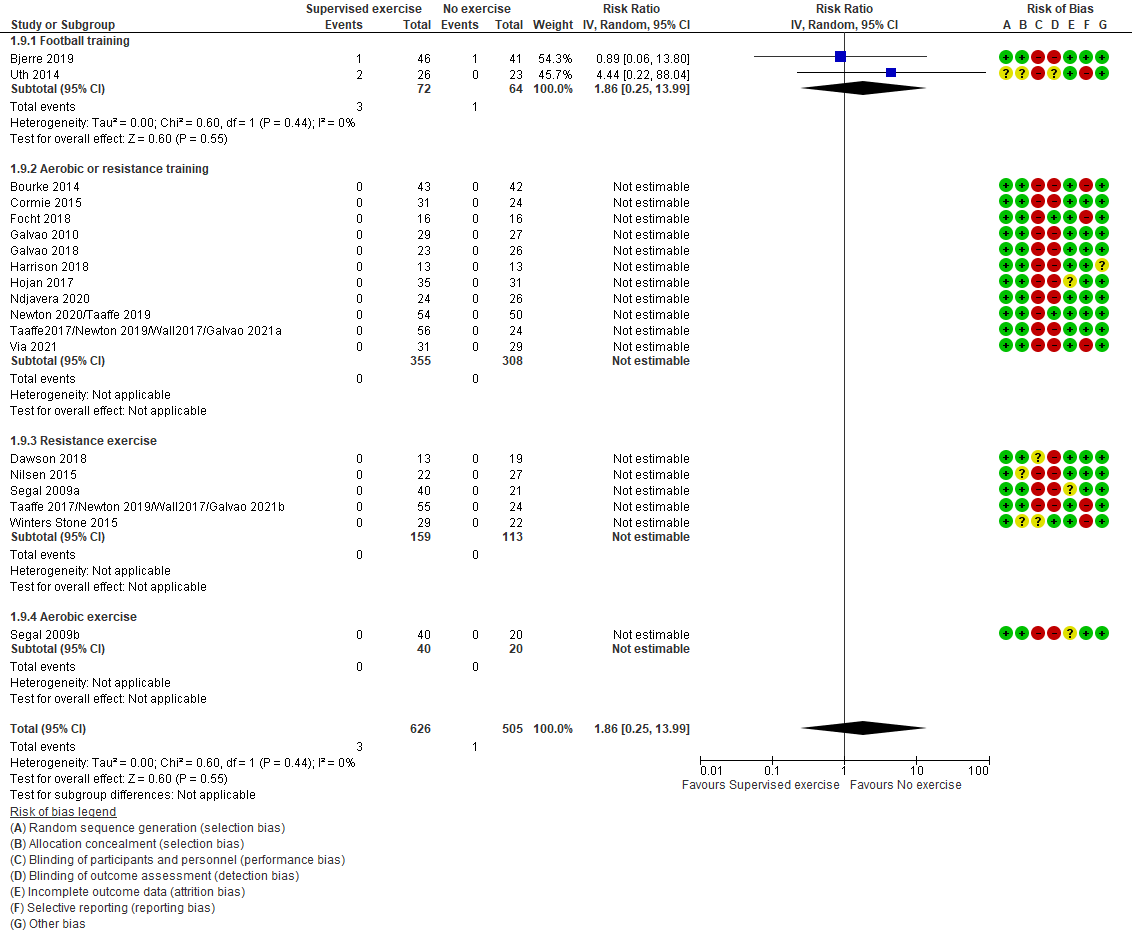


Fig. S3l. Exercise related injuries, number of patients with injuries. Supervised exercise therapy vs no exercise. Risk difference analysis.


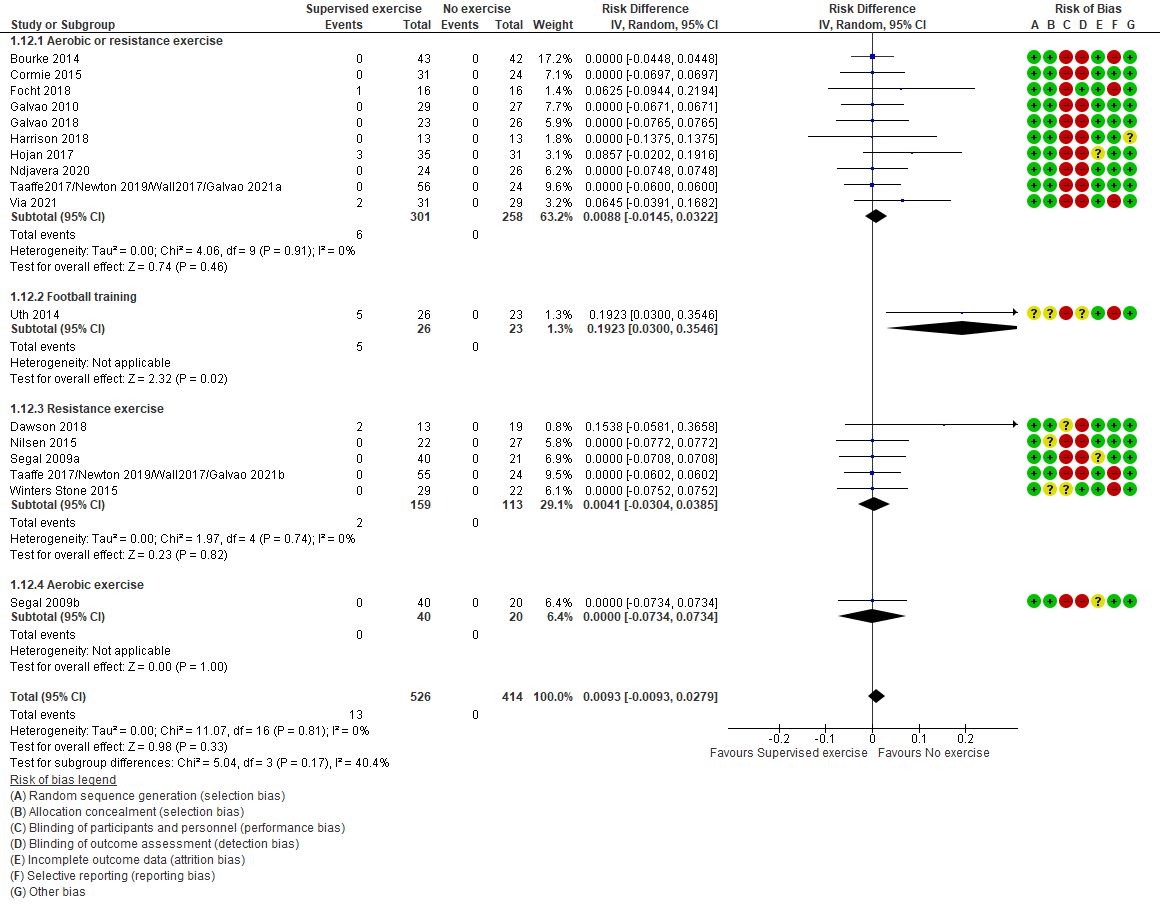


Fig. S3m. Exercise related injuries, number of patients with injuries. Supervised exercise therapy vs no exercise. Risk ratio analysis.


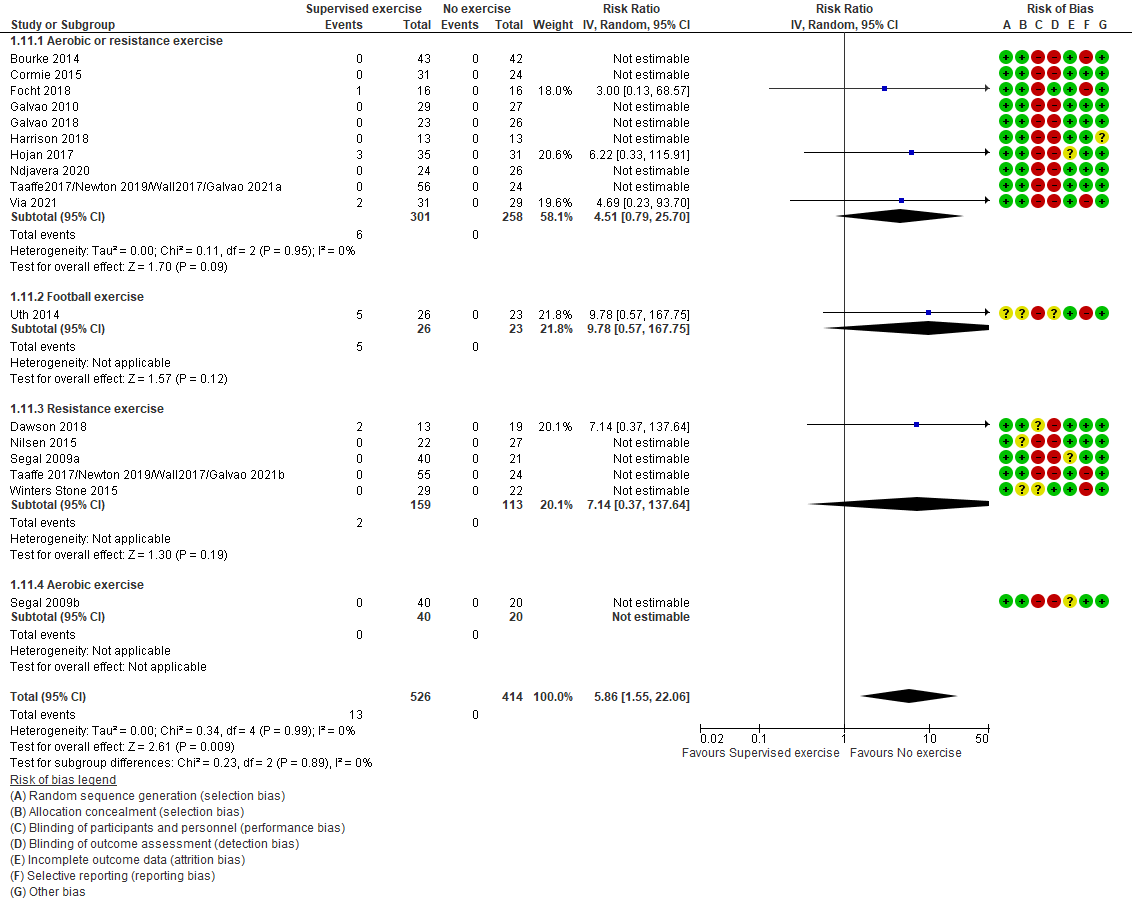


Fig. S3n. Dropout all causes. Supervised exercise therapy vs no exercise. Risk ratio analysis. Sub group start of exercise


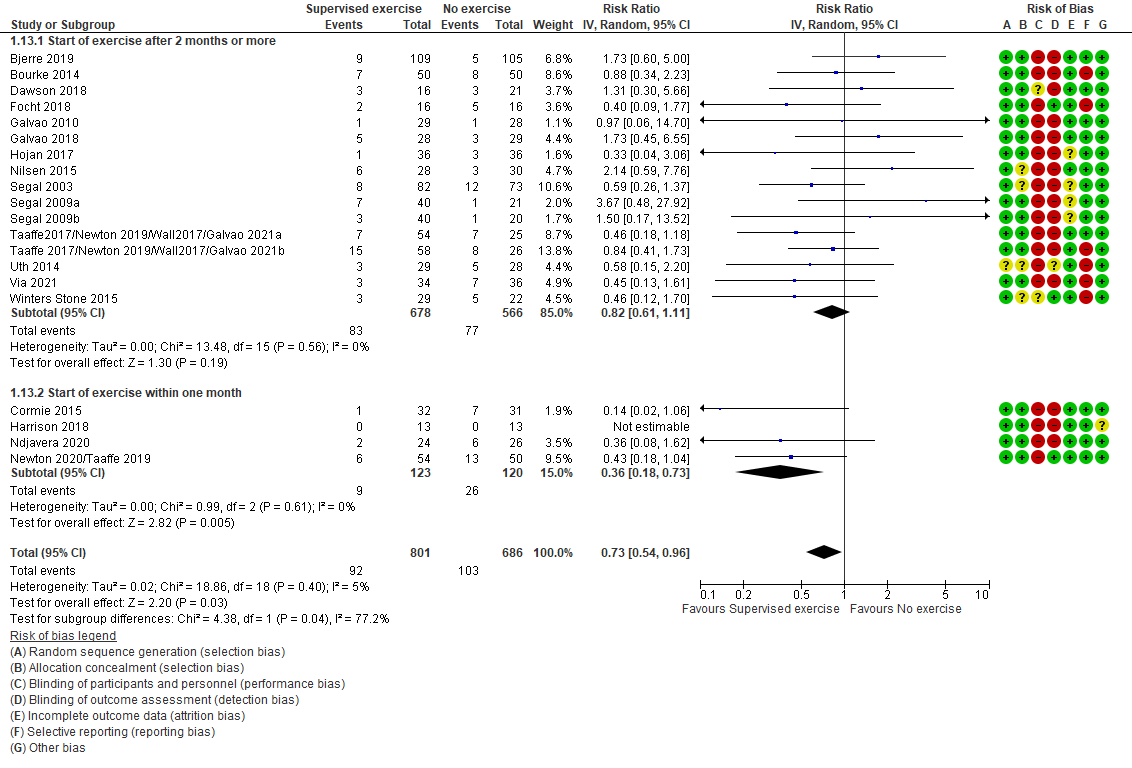


Fig. S3o. Dropout all causes. Supervised exercise therapy vs no exercise. Risk ratio analysis. Sub group type of exercise


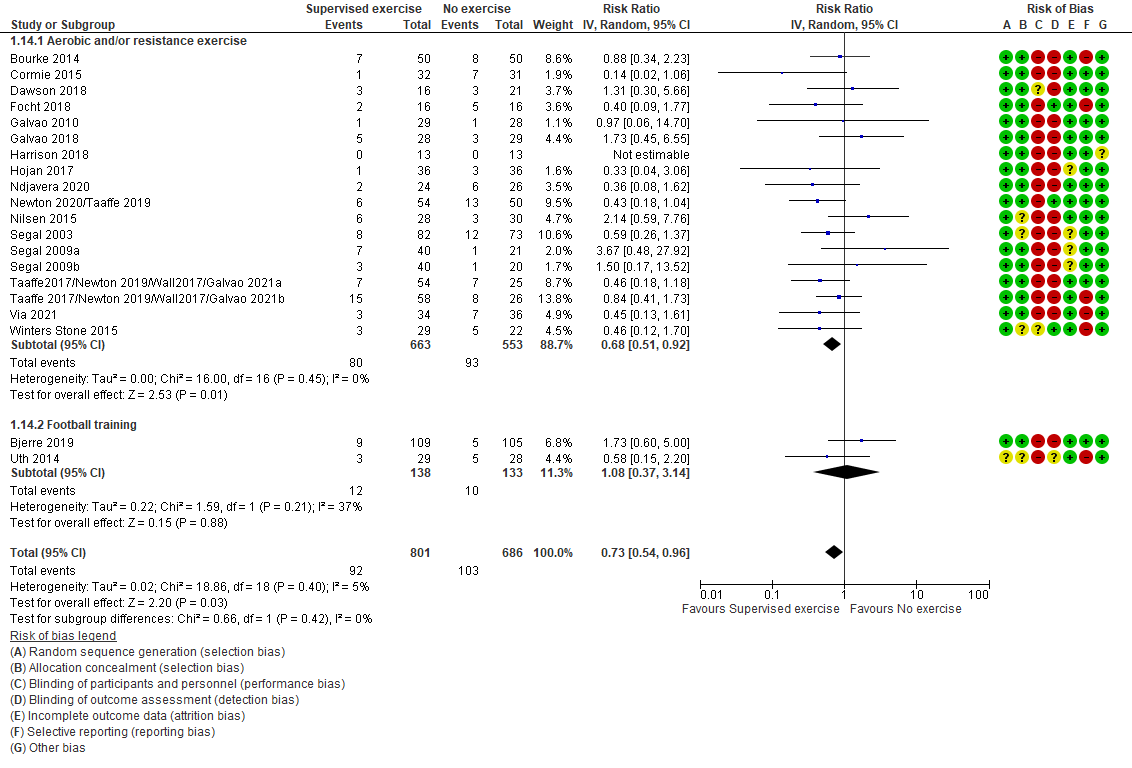


**Supplementary Figures S3. Funnel plots**

Funnel plot of comparison: Supervised training vs Usual care, outcome: Diagnose specific quality of life.


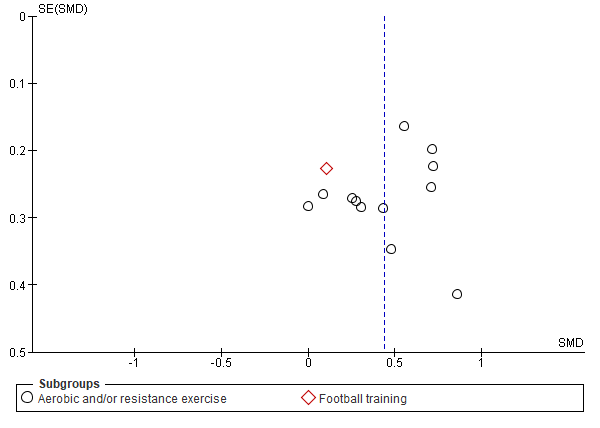


Funnel plot of comparison: Supervised training vs No supervised training, outcome: Physical performance measured by walking performance.


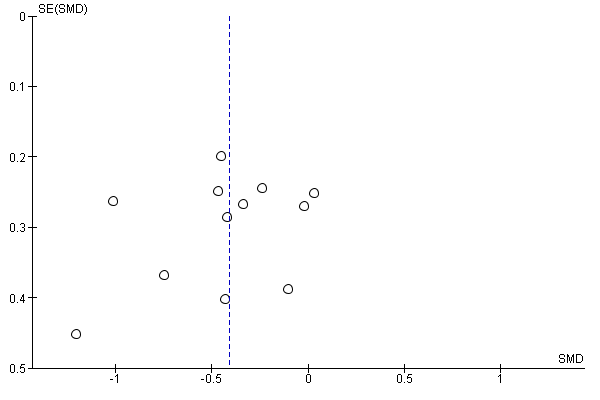


Funnel plot of comparison: Supervised training vs Usual care, outcome: Muscle strength.


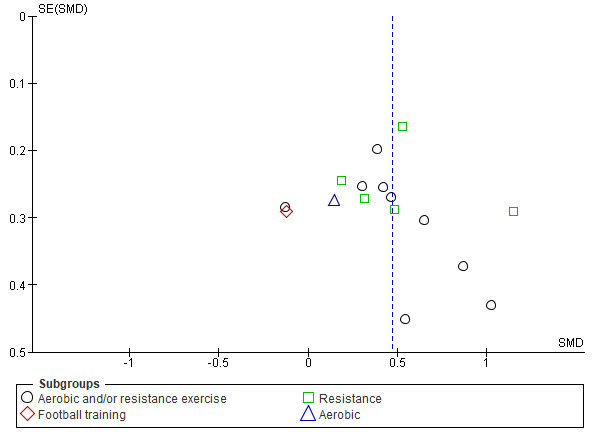


Funnel plot of comparison: Supervised training vs Usual care, outcome: Fractures, number of patients, risk difference analysis.


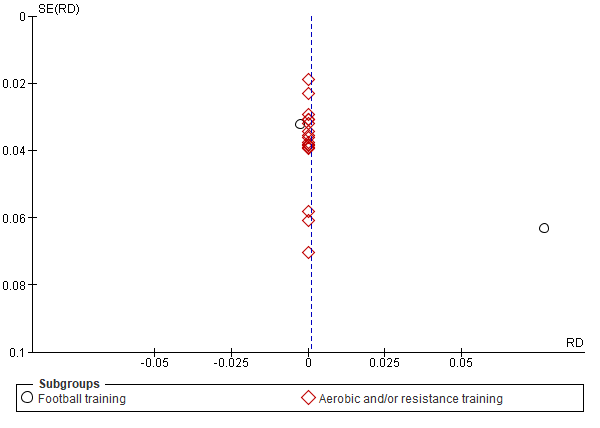


Funnel plot of comparison: Supervised training vs No supervised training, outcome: Exercise related injuries, number of patients, risk difference analysis.


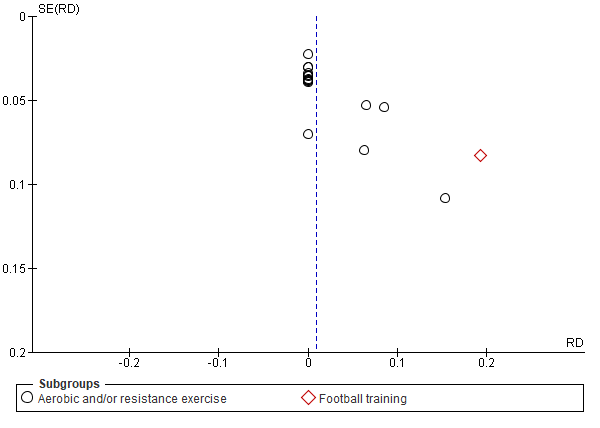


Funnel plot of comparison: Supervised training vs No supervised training, outcome: Dropout all causases, risk difference analysis.


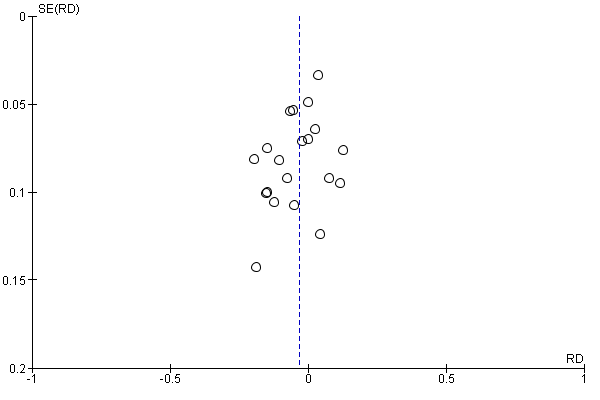


**Supplementary appendix**

**SEARCH DOCUMENTATION (Supplementary appendix)**

| Project title | Rehabilitation after prostate cancer |
| --- | --- |
| Lead / project manager/ method consultant | Marie-Louise Kirkegaard Mikkelsen, Danish Health Authority |
| Lead / method consultant | Anja Ussing, Danish Health Authority |
| Search specialist | Kirsten Birkefoss, Danish Health Authority |
| Last updated | 16.06.2021 |

**SEARCH STRATEGY – Original studies**

| **PICO** | **Should patients with prostate cancer, who begin castration-based treatment, be offered moderate to intensive supervised training instead of usual care?** |
| --- | --- |
| **Search terms** | See search terms in the search protocol below |
| **Inclusion and exclusion criterias** | Language: English, Danish, Norwegian, Swedish  Pub Year: 2005-2020  Population: Men with prostate cancer  Pub.types: Systematic reviews, meta analyses, randomized controlled trials or controlled trals |

**Information sources**

| **DATABASE** | **INTERFACE** |
| --- | --- |
| **Medline** | OVID |
| **EMBASE** | OVID |
| **PsycINFO** | OVID |
| **CINAHL** | EBSCO |
| **PEDRO** | Internet |

**Note**

- Search terms and inclusion/exclusion criterias are adapted to the different database languages.
- Duplicates have as thoroughly as possible been removed by the help of RefWorks.
- References found has been transferred to Covidence

**Search for systematic reviews and meta analyses**

Search date: 18.01.2016

**Medline**

Database(s): **Ovid MEDLINE(R) In-Process & Other Non-Indexed Citations and Ovid MEDLINE(R)** 1946 to Present
Search Strategy:

| **#** | **Searches** | **Results** |
| --- | --- | --- |
| 1 | exp Prostatic neoplasms/ | 100405 |
| 2 | ((prostatic or prostate) adj2 (cancer* or neoplasm* or tumo?r* or carcinoma* or oncolog*)).ti,ab,kw,sh. | 97795 |
| 3 | exp Castration/ or exp Androgen Antagonists/ | 65151 |
| 4 | ((androgen or hormon*) adj3 (deprivation or suppress* or ablation or block*)).ti,ab,kw,sh. | 13298 |
| 5 | ((hormone or ablation) adj3 therapy).ti,ab,kw,sh. | 26542 |
| 6 | 1 or 2 or 3 or 4 or 5 | 206360 |
| 7 | exp exercise/ | 133634 |
| 8 | exp exercise therapy/ | 34561 |
| 9 | exp Sports/ | 139940 |
| 10 | exp Physical Exertion/ | 53702 |
| 11 | exp Muscle strength/ | 20902 |
| 12 | exp Exercise Movement Techniques/ | 5879 |
| 13 | exp "Physical Education and Training"/ | 14128 |
| 14 | exp Hydrotherapy/ | 18159 |
| 15 | Weight-Bearing/ | 16201 |
| 16 | (exercis* or train* or ((muscl* or resistance or physical or endurance or strength* or aquatic*) adj3 (training or exercise* or strength* or fit* or condition* or performance))).ti,ab,kw,sh. | 856355 |
| 17 | (aerobic* or anaerobic* or movement therapy or watergym* or water gym* or aquagym or gym* or hydrotherap* or pilates or spinning).ti,ab,kw. | 122740 |
| 18 | (Skate* or skating or Jog* or running or zumba or Swim* or Bicycl* or cycling or walk* or powerwalk* or rowing or pacing or paced or titreted or soccer or football or ball game* or (play* adj2 ball) or (weight* adj3 (lift*or train* or exercis*))).ti,ab,kw. | 210331 |
| 19 | 7 or 8 or 9 or 10 or 11 or 12 or 13 or 14 or 15 or 16 or 17 or 18 | 1217164 |
| 20 | 6 and 19 | 6776 |
| 21 | limit 20 to (systematic reviews or meta analysis) | 312 |
| 22 | ((systematic adj3 (review* or overview* or study or studies or search* or approach*)) or meta analy* or meta-analy* or metaanaly*).ti,ab,kw,sh,pt. | 179982 |
| 23 | (pooled adj1 (data or analys*)).ti,ab. | 10418 |
| 24 | (pubmed or medline or embase or cochrane or "web of science" or psycinfo or psychinfo or scopus).ti,ab,jw. | 114054 |
| 25 | 22 or 23 or 24 | 237582 |
| 26 | 20 and 25 | 289 |
| 27 | 21 or 26 | 390 |
| 28 | limit 27 to (yr="2005 -Current" and (danish or english or german or norwegian or swedish)) | 299 |

**Embase**

Database(s): **Embase** 1974 to 2016 January 15
Search Strategy:

| **#** | **Searches** | **Results** |
| --- | --- | --- |
| 1 | exp Prostate cancer/ or exp Prostate tumor/ | 176115 |
| 2 | ((prostatic or prostate) adj2 (cancer* or neoplasm* or tumo?r* or carcinoma* or oncolog*)).ti,ab,kw,sh. | 146205 |
| 3 | exp Castration/ or exp Androgen Antagonists/ or exp androgen deprivation therapy/ or hormonal therapy/ | 101431 |
| 4 | ((androgen or hormon*) adj3 (deprivation or suppress* or ablation or block*)).ti,ab,kw,sh. | 19115 |
| 5 | (((hormone or ablation) adj3 therapy) or adt).ti,ab,kw,sh. | 44677 |
| 6 | 1 or 2 or 3 or 4 or 5 | 309314 |
| 7 | exp exercise/ | 245663 |
| 8 | exp exercise therapy/ | 55643 |
| 9 | exp Sport/ | 115992 |
| 10 | exp Physical Exertion/ | 245663 |
| 11 | exp Muscle strength/ | 41106 |
| 12 | exp Exercise Movement Techniques/ | 55643 |
| 13 | exp "Physical Education and Training"/ | 10906 |
| 14 | exp Hydrotherapy/ | 3729 |
| 15 | Weight-Bearing/ | 22027 |
| 16 | (exercis* or train* or ((muscl* or resistance or physical or endurance or strength* or aquatic*) adj3 (training or exercise* or strength* or fit* or condition* or performance))).ti,ab,kw,sh. | 1118597 |
| 17 | (aerobic* or anaerobic* or movement therapy or watergym* or water gym* or aquagym or gym* or hydrotherap* or pilates or spinning).ti,ab,kw. | 151987 |
| 18 | (Skate* or skating or Jog* or running or zumba or Swim* or Bicycl* or cycling or walk* or powerwalk* or rowing or pacing or paced or titreted or soccer or football or ball game* or (play* adj2 ball) or (weight* adj3 (lift*or train* or exercis*))).ti,ab,kw. | 273609 |
| 19 | 7 or 8 or 9 or 10 or 11 or 12 or 13 or 14 or 15 or 16 or 17 or 18 | 1533687 |
| 20 | 6 and 19 | 11194 |
| 21 | limit 20 to ("systematic review" or meta analysis) | 434 |
| 22 | ((systematic adj3 (review* or overview* or study or studies or search* or approach*)) or meta analy* or meta-analy* or metaanaly*).ti,ab,kw,sh,pt. | 253708 |
| 23 | (pooled adj1 (data or analys*)).ti,ab. | 15685 |
| 24 | (pubmed or medline or embase or cochrane or "web of science" or psycinfo or psychinfo or scopus).ti,ab,jw. | 144095 |
| 25 | 22 or 23 or 24 | 329247 |
| 26 | 20 and 25 | 746 |
| 27 | 21 or 26 | 773 |
| 28 | limit 27 to (yr="2005 -Current" and (danish or english or german or norwegian or swedish)) | 641 |

**Cochrane Reviews**

**ID Search Hits**

#1 [mh ^"Prostatic neoplasms"] 3572

#2 ((prostatic or prostate) near/2 (cancer* or neoplasm* or tumo*r* or carcinoma* or oncolog*)) 7419

#3 MeSH descriptor: [Castration] explode all trees 766

#4 MeSH descriptor: [Androgen Antagonists] explode all trees 764

#5 ((androgen or hormone) near/3 (deprivation or suppression or ablation or block*)):ti,ab,kw 1125

#6 ((hormone or ablation) near/3 therapy):ti,ab,kw 5512

#7 #1 or #2 or #3 or #4 or #5 or #6 13416

#8 MeSH descriptor: [Exercise] explode all trees 14454

#9 MeSH descriptor: [Exercise Therapy] explode all trees 7533

#10 MeSH descriptor: [Sports] explode all trees 10301

#11 MeSH descriptor: [Physical Exertion] explode all trees 3341

#12 MeSH descriptor: [Muscle Strength] explode all trees 2737

#13 MeSH descriptor: [Exercise Movement Techniques] explode all trees 1342

#14 MeSH descriptor: [Physical Education and Training] explode all trees 1387

#15 MeSH descriptor: [Hydrotherapy] explode all trees 1293

#16 MeSH descriptor: [Weight-Bearing] explode all trees 745

#17 (exercis* or train* or ((muscl* or resistance or physical or endurance or strength* or aquatic*) near/3 (training or exercise* or strength* or fit* or condition* or performance))):ti,ab,kw 84927

#18 (aerobic* or anaerobic* or movement therapy or watergym* or water gym* or aquagym or gym* or hydrotherap* or pilates or spinning):ti,ab,kw 12178

#19 (Skate* or skating or Jog* or running or zumba or Swim* or Bicycl* or cycling or walk* or powerwalk* or rowing or pacing or paced or titreted or soccer or football or ball game* or (play* near/2 ball) or (weight* near/3 (lift* or train* or exercis*))):ti,ab,kw 24588

#20 #8 or #9 or #10 or #11 or #12 or #13 or #14 or #15 or #16 or #17 or #18 or #19 100635

#21 #7 and #20 Publication Year from 2005 to 2016, in Cochrane Reviews (Reviews and Protocols) and Other Reviews 60

**Cinahl**

| **#** | **Query** | **Limiters/Expanders** | **Results** | **Action** |
| --- | --- | --- | --- | --- |
| S16 | s9 AND s14 | Limiters - Published Date: 20050101-20151231; Language: Danish, English, German, Norwegian, Swedish | 34 |  |
| S15 | s9 AND s14 |  | 38 |  |
| S14 | S10 OR S11 OR S12 OR S13 |  | 133,715 |  |
| S13 | AB (pubmed or medline or embase or cochrane or "web of science" or psycinfo or psychinfo or scopus) |  | 25,750 |  |
| S12 | TX (pooled N1 (data or analys*)) |  | 2,621 |  |
| S11 | TX (((systematic or method*) N3 (review* or overview* or study or studies or search* or approach*)) or meta analy* or meta-analy* or metaanaly*) |  | 121,963 |  |
| S10 | PT (Systematic Review or Meta Analysis) |  | 40,906 |  |
| S9 | s3 AND s8 |  | 474 |  |
| S8 | S4 OR S5 OR S6 OR S7 |  | 253,083 |  |
| S7 | (Skate* or skating or Jog* or running or zumba or Swim* or Bicycl* or cycling or walk* or powerwalk* or rowing or pacing or paced or titreted or soccer or football or ball game* or (play* N2 ball) or (weight* N3 (lift* or train* or exercis*))) |  | 58,183 |  |
| S6 | (aerobic* or anaerobic* or movement therapy or watergym* or water gym* or aquagym or gym* or hydrotherap* or pilates or spinning) |  | 14,621 |  |
| S5 | (exercis* or train* or ((muscl* or resistance or physical or endurance or strength* or aquatic*) N3 (training or exercise* or strength* or fit* or condition* or performance))) |  | 196,804 |  |
| S4 | MH ("Exercise+" OR "Exercise therapy+" OR "Sports+" OR "Physical Exertion+" OR "Muscle strength+" OR "Exercise Movement Techniques+" OR "Physical Education and Training+" OR "Hydrotherapy+" OR "Weight-Bearing") |  | 98,820 |  |
| S3 | S1 OR S2 |  | 12,661 |  |
| S2 | ((prostatic or prostate) N2 (cancer* or neoplasm* or tumo?r* or carcinoma* or oncolog*)) |  | 12,661 |  |
| S1 | (MH "Prostatic Neoplasms+") |  | 11,391 |  |

**Pedro 30 hits**

Abstract & Title: prostate cancer

Method: systematic review

Published since: 2005

**Search for randomized controlled studies/controlled studies**

Search date: 18.01.2016

**Medline**

Database(s): **Ovid MEDLINE(R) In-Process & Other Non-Indexed Citations and Ovid MEDLINE(R)** 1946 to Present
Search Strategy:

| **#** | **Searches** | **Results** |
| --- | --- | --- |
| 1 | exp Prostatic neoplasms/ | 101156 |
| 2 | ((prostatic or prostate) adj2 (cancer* or neoplasm* or tumo?r* or carcinoma* or oncolog*)).ti,ab,kw,sh. | 98888 |
| 3 | exp Castration/ or exp Androgen Antagonists/ | 65362 |
| 4 | ((androgen* or hormon*) adj3 (deprivation or suppress* or suppress* or ablat* or block* or withdraw* or effect* or therapy or treatment)).ti,ab,kw. | 87238 |
| 5 | ((hormon* or ablation or endocrine or antiandrogen or anti-androgen) adj3 (therapy or treat*)).ti,ab,kw. | 64148 |
| 6 | 1 or 2 or 3 or 4 or 5 | 261948 |
| 7 | exp exercise/ | 134945 |
| 8 | exp exercise therapy/ | 34963 |
| 9 | exp Sports/ | 141073 |
| 10 | exp Physical Exertion/ | 53802 |
| 11 | exp Muscle strength/ | 21247 |
| 12 | exp Exercise Movement Techniques/ | 5913 |
| 13 | exp "Physical Education and Training"/ | 14164 |
| 14 | exp Hydrotherapy/ | 18209 |
| 15 | Weight-Bearing/ | 16323 |
| 16 | (exercis* or train*).ti,ab,kw,sh. | 605302 |
| 17 | ((muscl* or resistance or physical or endurance or strength* or aquatic*) adj3 (training or exercise* or strength* or fit* or condition*)).ti,ab,kw. | 329896 |
| 18 | (aerobic* or anaerobic* or movement therapy or watergym* or water gym* or aquagym or gym* or hydrotherap* or pilates or spinning).ti,ab,kw. | 123876 |
| 19 | (Skate* or skating or Jog* or powerwalk* or zumba or Swim* or Bicycl* or cycling or walk* or rowing or pacing or paced or titreted or soccer or football or handball or cricket or hockey or baseball or basketball or volleyball or ball game* or (play* adj2 ball) or (weight* adj3 (lift* or train* or exercis*))).ti,ab,kw. | 227278 |
| 20 | 7 or 8 or 9 or 10 or 11 or 12 or 13 or 14 or 15 or 16 or 17 or 18 or 19 | 1232553 |
| 21 | 6 and 20 | 8959 |
| 22 | limit 21 to (randomized controlled trial or controlled clinical trial) | 680 |
| 23 | (((random* or control?ed or crossover or cross-over or blind* or mask*) adj3 (trial*1 or study or studies or analy*)) or rct).ti,ab,kw. | 468533 |
| 24 | (placebo* or single-blind* or double-blind* or triple-blind*).ti,ab,kw. | 225335 |
| 25 | ((single or double or triple) adj2 (blind* or mask*)).ti,ab,kw. | 139885 |
| 26 | 23 or 24 or 25 | 548388 |
| 27 | 21 and 26 | 971 |
| 28 | 22 or 27 | 1223 |
| 29 | limit 28 to (yr="2015 -Current" and (danish or english or german or norwegian or swedish)) | 79 |

**Embase**

Database(s): **Embase** 1974 to 2016 February 23
Search Strategy:

| **#** | **Searches** | **Results** |
| --- | --- | --- |
| 1 | exp Prostate cancer/ or exp Prostate tumor/ | 177381 |
| 2 | ((prostatic or prostate) adj2 (cancer* or neoplasm* or tumo?r* or carcinoma* or oncolog*)).ti,ab,kw,sh. | 147496 |
| 3 | exp Castration/ or exp Androgen Antagonists/ or exp androgen deprivation therapy/ or hormonal therapy/ | 102030 |
| 4 | ((androgen* or hormon*) adj3 (deprivation or suppress* or supress* or ablat* or block* or withdraw* or effect* or therapy or treatment)).ti,ab,kw. | 114466 |
| 5 | ((hormon* or ablation or endocrine or antiandrogen or anti-androgen) adj3 (therapy or treat*)).ti,ab,kw. | 89890 |
| 6 | 1 or 2 or 3 or 4 or 5 | 370510 |
| 7 | exp exercise/ | 247854 |
| 8 | exp exercise therapy/ | 56133 |
| 9 | exp Sports/ | 117011 |
| 10 | exp Physical Exertion/ | 247854 |
| 11 | exp Muscle strength/ | 41660 |
| 12 | exp Exercise Movement Techniques/ | 56133 |
| 13 | exp "Physical Education and Training"/ | 10962 |
| 14 | exp Hydrotherapy/ | 3749 |
| 15 | Weight-Bearing/ | 22153 |
| 16 | (exercis* or train*).ti,ab,kw,sh. | 823087 |
| 17 | ((muscl* or resistance or physical or endurance or strength* or aquatic*) adj3 (training or exercise* or strength* or fit* or condition*)).ti,ab,kw. | 397995 |
| 18 | (aerobic* or anaerobic* or movement therapy or watergym* or water gym* or aquagym or gym* or hydrotherap* or pilates or spinning).ti,ab,kw. | 153257 |
| 19 | (Skate* or skating or Jog* or powerwalk* or zumba or Swim* or Bicycl* or cycling or walk* or rowing or pacing or paced or titreted or soccer or football or handball or cricket or hockey or baseball or basketball or volleyball or ball game* or (play* adj2 ball) or (weight* adj3 (lift* or train* or exercis*))).ti,ab,kw. | 293835 |
| 20 | 7 or 8 or 9 or 10 or 11 or 12 or 13 or 14 or 15 or 16 or 17 or 18 or 19 | 1549403 |
| 21 | 6 and 20 | 13832 |
| 22 | limit 21 to (randomized controlled trial or controlled clinical trial) | 982 |
| 23 | (((random* or control?ed or crossover or cross-over or blind* or mask*) adj3 (trial*1 or study or studies or analy*)) or rct).ti,ab,kw. | 634432 |
| 24 | (placebo* or single-blind* or double-blind* or triple-blind*).ti,ab,kw. | 303599 |
| 25 | ((single or double or triple) adj2 (blind* or mask*)).ti,ab,kw. | 186153 |
| 26 | 23 or 24 or 25 | 741870 |
| 27 | 21 and 26 | 1685 |
| 28 | 22 or 27 | 2028 |
| 29 | limit 28 to (yr="2015 -Current" and (danish or english or german or norwegian or swedish)) | 126 |

**Cinahl**

| **#** | **Query** | **Limiters/Expanders** | **Results** |
| --- | --- | --- | --- |
| S14 | (S10 OR S11 OR S12) AND (S8 AND S13) | Limiters - Published Date: 20150101-20160231; Language: Danish, English, German, Norwegian, Swedish | 14 |
| S13 | S10 OR S11 OR S12 |  | 127,808 |
| S12 | TX (placebo* or single-blind* or double-blind* or triple-blind* or ((single or double or triple) N1 (blind* or mask*)) |  | 47,379 |
| S11 | TX (((random* or control#ed or crossover or cross-over or blind* or mask*) N3 (trial* or study or studies or analy*)) or rct) |  | 121,614 |
| S10 | PT (Randomized Controlled Trial) |  | 30,281 |
| S9 | s3 AND s8 |  | 1,176 |
| S8 | (S1 or S2 or S3) AND (S4 OR S5 OR S6 OR S7) |  | 1,598 |
| S7 | (Skate* or skating or Jog* or running or zumba or Swim* or Bicycl* or cycling or walk* or powerwalk* or rowing or pacing or paced or titreted or soccer or football or handball or cricket or baseball or basketball or hockey or volleyball or ball game* or (play* N2 ball) or (weight* N3 (lift* or train* or exercis*))) |  | 64,187 |
| S6 | (aerobic* or anaerobic* or movement therapy or watergym* or water gym* or aquagym or gym* or hydrotherap* or pilates or spinning) |  | 14,708 |
| S5 | (exercis* or train* or ((muscl* or resistance or physical or endurance or strength* or aquatic*) N3 (training or exercise* or strength* or fit* or condition* or performance))) |  | 197,822 |
| S4 | MH ("Exercise+" OR "Exercise therapy+" OR "Sports+" OR "Physical Exertion+" OR "Muscle strength+" OR "Exercise Movement Techniques+" OR "Physical Education and Training+" OR "Hydrotherapy+" OR "Weight-Bearing") |  | 99,338 |
| S3 | TX ((androgen* or hormon*) N3 (deprivation or suppress* or supress* or ablat* or block* or withdraw* or effect* or therapy or treatment)) OR TX ((hormon* or ablation or endocrine or antiandrogen or anti-androgen) N3 (therapy or treat*)) |  | 15,812 |
| S2 | ((prostatic or prostate) N2 (cancer* or neoplasm* or tumo?r* or carcinoma* or oncolog*)) |  | 12,722 |
| S1 | (MH "Prostatic Neoplasms+") |  | 11,423 |

**Pedro**

**Abstract & Title:** Prostate cancer, Prostate tumor

**Therapy:** Fitness training OR Strength training

**Method:** Clinical trial

**Published since:** 2015

14 hits

**Updated search for randomized controlled studies/controlled studies 2016-2020**

Search date: 25.06.2020

**Medline**

Database(s): **Ovid MEDLINE(R) and Epub Ahead of Print, In-Process & Other Non-Indexed Citations, Daily and Versions(R)**1946 to June 23, 2020
Search Strategy:

| **#** | **Searches** | **Results** |
| --- | --- | --- |
| 1 | exp Prostatic neoplasm/ | 127546 |
| 2 | ((prostatic or prostate or ((male* or men) and (urogenital or genital))) and (cancer* or neoplas* or tumo?r* or carcinoma* or oncolog* or postcancer or post-cancer)).ti,bt,ab,kf. | 160550 |
| 3 | Prostatectomy/ | 28539 |
| 4 | (androgen deprivation therapy or prostatect* or post-prostatect* or castration).ti,bt,ab,kf. | 54310 |
| 5 | or/1-4 | 203592 |
| 6 | exp exercise/ | 194050 |
| 7 | exp exercise therapy/ | 50442 |
| 8 | exp Sports/ | 181956 |
| 9 | exp Physical Exertion/ | 56266 |
| 10 | exp Muscle strength/ | 33536 |
| 11 | exp Exercise Movement Techniques/ | 8155 |
| 12 | exp "Physical Education and Training"/ | 13435 |
| 13 | physical therapy modalities/ | 36579 |
| 14 | Weight-Bearing/ | 20158 |
| 15 | (exercis* or train* or stepped care or physical therap* or physiotherap* or kinesiotherapy).ti,bt,kf. | 287636 |
| 16 | (physical adj1 (education or program*)).ti,bt,ab,kf. | 5800 |
| 17 | ((exercise or movement) adj3 (session* or training or technique* or physical or isometric or therap* or program* or class*)).ti,bt,ab,kf. | 69086 |
| 18 | ((physical therapy or physiotherapy) adj3 (exercise* or session* or training or technique* or isometric or program* or class*)).ti,bt,ab,kf. | 4562 |
| 19 | ((muscl* or neuromuscul* or neuro-muscul* or resistance or physical or kinesiotherap* or kinesitherap* or endurance or strength* or balance or postur* or gait or threshold* or treadmill* or aquatic* or trunk or graded or gradual or progressive or controlled or incremented or symptom-based or symptombased or symptom based or guided or supervis* or coached or coaching or stepped care or stepwise or step-wise) adj3 (train* or retrain* or re-train* or exercise* or rehabilitation or strength* or fitness or fit or condition* or exposure or activit* or performance or exertion or load*)).ti,bt,ab,kf. | 720662 |
| 20 | (active adj1 rehabilitation).ti,bt,ab,kf. | 443 |
| 21 | (aerobic* or anaerobic* or movement therapy or pilates or watergym* or wateraerobic* or water-gym* or water-aerobic* or aquagym or ((water or aqva or aqua or pool) adj1 (aerobic* or gym*)) or gym or gymnastic* or ((warm water or aqvatic or aquatic) adj4 (exercis* or train* or rehab*))).ti,bt,ab,kf. | 150391 |
| 22 | (running or skating or skiing or Jog* or zumba or kettlebelt or Swim* or Bicycl* or cycling or bicycling or fitness bike* or exercise bike* or spinning or walking or rowing or rower or water-rower* or waterrower* or cross-train* or crosstrain* or pacing or paced or titreted or soccer or football or handball or cricket or hockey or baseball or basketball or volleyball or ball game* or (play* adj2 ball) or (weight* adj3 (lift* or train* or exercis*))).ti,bt,ab,kf. | 334456 |
| 23 | ((upper body or lower body) adj2 (exercise* or training or program*)).ti,bt,ab,kf. | 852 |
| 24 | ((mckenzie or mechanical or Alexander or William or Feldenkrais) adj2 (technique* or model* or tool* or exercise* or therapy)).ti,bt,ab,kf. | 7232 |
| 25 | ((non-pharma* or nonpharma* or non pharma* or non-drug or nondrug or non drug or non-surgical or nonsurgical or non surgical or non-surgery or nonsurgery or non surgery or non-invasive or noninvasive or non invasive or complimentary or integrat* or holistic or multi-modal* or multimodal* or Multidisciplinary or Multi-disciplinary or Interdisciplinary or Inter-disciplinary or complex or multimodal* or multi-modal* or cross-disciplinary or crossdisciplinary or multi-dimensional or multidimensional or multi-facet* or multifacet* or comprehensive or multiple or rehabilitati*) adj6 (therap* or treat* or intervention* or action* or program* or strateg* or protocol* or support* or approach* or evaluation or rehabilitation or care)).ti,bt,kf. | 180800 |
| 26 | or/6-25 | 1605154 |
| 27 | 5 and 26 | 5574 |
| 28 | limit 27 to (randomized controlled trial or controlled clinical trial) | 358 |
| 29 | (((random* or cluster-random* or quasi-random* or control?ed or crossover or cross-over or blind* or mask*) adj4 (trial*1 or study or studies or analy*)) or rct).ti,bt,ab,kf,hw. | 1059438 |
| 30 | (placebo* or single-blind* or double-blind* or triple-blind*).ti,bt,kf,hw. | 224687 |
| 31 | ((single or double or triple) adj2 (blind* or mask*)).ti,bt,kf,hw. | 196015 |
| 32 | ((patient* or person* or participant* or population* or allocat* or assign*) adj3 random*).ti,bt,ab,kf. | 247365 |
| 33 | or/29-32 | 1143492 |
| 34 | 27 and 33 | 824 |
| 35 | 28 or 34 | 824 |
| 36 | limit 35 to (yr="2015-2020" and (english or danish or norwegian or swedish)) | 356 |

**Embase**

Database(s): **Embase**1974 to 2020 June 24
Search Strategy:

| **#** | **Searches** | **Results** |
| --- | --- | --- |
| 1 | exp Prostate cancer/ or exp Prostate tumor/ | 240885 |
| 2 | androgen deprivation therapy/ | 12204 |
| 3 | Prostatectomy/ | 55850 |
| 4 | ((prostatic or prostate or ((male* or men) and (urogenital or genital))) and (cancer* or neoplas* or tumo?r* or carcinoma* or oncolog* or postcancer or post-cancer)).ti,ab,kw. | 237352 |
| 5 | (androgen deprivation therapy or prostatect* or post-prostatect* or castration).ti,ab,kw. | 80518 |
| 6 | or/1-5 | 310954 |
| 7 | exp exercise/ | 337556 |
| 8 | exp kinesiotherapy/ | 77424 |
| 9 | physiotherapy/ | 83871 |
| 10 | home physiotherapy/ | 319 |
| 11 | physical activity/ or cycling/ or jogging/ or jumping/ or lifting effort/ or running/ or scratching/ or stretching/ or swimming/ or exp walking/ or weight bearing/ or weight lifting/ | 359162 |
| 12 | exp Muscle strength/ | 62692 |
| 13 | exp Physical education/ | 11160 |
| 14 | (exercis* or train* or stepped care or physical therap* or physiotherap* or kinesiotherapy).ti,kw. | 365591 |
| 15 | (physical adj1 (education or program*)).ti,ab,kw. | 6961 |
| 16 | ((exercise or movement) adj3 (session* or training or technique* or physical or isometric or therap* or program* or class*)).ti,ab,kw. | 94745 |
| 17 | ((physical therapy or physiotherapy) adj3 (exercise* or session* or training or technique* or isometric or program* or class*)).ti,ab,kw. | 8055 |
| 18 | ((muscl* or neuromuscul* or neuro-muscul* or resistance or physical or kinesiotherap* or kinesitherap* or endurance or strength* or balance or postur* or gait or threshold* or treadmill* or aquatic* or trunk or graded or gradual or progressive or controlled or incremented or symptom-based or symptombased or symptom based or guided or supervis* or coached or coaching or stepped care or stepwise or step-wise) adj3 (train* or retrain* or re-train* or exercise* or rehabilitation or strength* or fitness or fit or condition* or exposure or activit* or performance or exertion or load*)).ti,ab,kw. | 885672 |
| 19 | (active adj1 rehabilitation).ti,ab,kw. | 656 |
| 20 | (aerobic* or anaerobic* or movement therapy or pilates or watergym* or wateraerobic* or water-gym* or water-aerobic* or aquagym or ((water or aqva or aqua or pool) adj1 (aerobic* or gym*)) or gym or gymnastic* or ((warm water or aqvatic or aquatic) adj4 (exercis* or train* or rehab*))).ti,ab,kw. | 181078 |
| 21 | (running or skating or skiing or Jog* or zumba or kettlebelt or Swim* or Bicycl* or cycling or bicycling or fitness bike* or exercise bike* or spinning or walking or rowing or rower or water-rower* or waterrower* or cross-train* or crosstrain* or pacing or paced or titreted or soccer or football or handball or cricket or hockey or baseball or basketball or volleyball or ball game* or (play* adj2 ball) or (weight* adj3 (lift* or train* or exercis*))).ti,ab,kw. | 421787 |
| 22 | ((upper body or lower body) adj2 (exercise* or training or program*)).ti,ab,kw. | 967 |
| 23 | ((mckenzie or mechanical or Alexander or William or Feldenkrais) adj2 (technique* or model* or tool* or exercise* or therapy)).ti,ab,kw. | 8382 |
| 24 | ((non-pharma* or nonpharma* or non pharma* or non-drug or nondrug or non drug or non-surgical or nonsurgical or non surgical or non-surgery or nonsurgery or non surgery or non-invasive or noninvasive or non invasive or complimentary or integrat* or holistic or multi-modal* or multimodal* or Multidisciplinary or Multi-disciplinary or Interdisciplinary or Inter-disciplinary or complex or multimodal* or multi-modal* or cross-disciplinary or crossdisciplinary or multi-dimensional or multidimensional or multi-facet* or multifacet* or comprehensive or multiple or rehabilitati*) adj6 (therap* or treat* or intervention* or action* or program* or strateg* or protocol* or support* or approach* or evaluation or rehabilitation or care)).ti,kw. | 250469 |
| 25 | or/7-24 | 2081626 |
| 26 | 6 and 25 | 11286 |
| 27 | limit 26 to (randomized controlled trial or controlled clinical trial) | 762 |
| 28 | (((random* or cluster-random* or quasi-random* or control?ed or crossover or cross-over or blind* or mask*) adj4 (trial*1 or study or studies or analy*)) or rct).ti,ab,kw. | 972056 |
| 29 | (placebo* or single-blind* or double-blind* or triple-blind*).ti,ab. | 398953 |
| 30 | ((single or double or triple) adj2 (blind* or mask*)).ti,ab. | 240156 |
| 31 | ((patient* or person* or participant* or population* or allocat* or assign*) adj3 random*).ti,ab. | 342913 |
| 32 | or/28-31 | 1240649 |
| 33 | 26 and 32 | 1397 |
| 34 | 27 or 33 | 1590 |
| 35 | limit 34 to (yr="2015-2020" and (english or danish or norwegian or swedish)) | 691 |

**Cinahl**

| **#** | **Query** | **Limiters/Expanders** | **Results** |
| --- | --- | --- | --- |
| S21 | S15 AND S20 | Limiters - Published Date: 20150101-20200630; Language: Danish, English, Norwegian, Swedish | 151 |
| S20 | S16 OR S17 OR S18 OR S19 |  | 406,492 |
| S19 | ((patient* or person* or participant* or population* or allocat* or assign*) N3 (random* or blind* or mask*)) |  | 151,499 |
| S18 | (placebo* or single-blind* or double-blind* or triple-blind* or ((single or double or triple) N1 (blind* or mask*)) |  | 112,566 |
| S17 | (((random* or cluster-random* or quasi-random* or control#ed or crossover or cross-over or blind* or mask*) N4 (trial* or study or studies or analy*)) or rct) |  | 336,563 |
| S16 | PT Randomized Controlled Trial OR MH "Randomized Controlled Trial+" OR PT "Controlled Clinical Trial" OR MH "Controlled Clinical Trial+" |  | 131,813 |
| S15 | S3 AND S14 |  | 2,094 |
| S14 | S4 OR S5 OR S6 OR S7 OR S8 OR S9 OR S10 OR S11 OR S12 OR S13 |  | 711,134 |
| S13 | TI ((non-surgical or nonsurgical or non surgical or non-surgery or nonsurgery or non surgery or non-invasive or noninvasive or non invasive or complimentary or integrat* or holistic or multi-modal* or multimodal* or Multidisciplinary or Multi-disciplinary or Interdisciplinary or Inter-disciplinary or complex or multimodal* or multi-modal* or cross-disciplinary or crossdisciplinary or multi-dimensional or multidimensional or multi-facet* or multifacet* or comprehensive or multiple) N3 (therap* or treat* or intervention* or action* or program* or strateg* or protocol* or support* or approach* or evaluation or care)) OR SU ((non-surgical or nonsurgical or non surgical or non-surgery or nonsurgery or non surgery or non-invasive or noninvasive or non invasive or complimentary or integrat* or holistic or multi-modal* or multimodal* or Multidisciplinary or Multi-disciplinary or Interdisciplinary or Inter-disciplinary or complex or multimodal* or multi-modal* or cross-disciplinary or crossdisciplinary or multi-dimensional or multidimensional or multi-facet* or multifacet* or comprehensive or multiple) N3 (therap* or treat* or intervention* or action* or program* or strateg* or protocol* or support* or approach* or evaluation or care)) |  | 105,557 |
| S12 | ((upper body or lower body) N2 (exercise* or training or program*)) |  | 588 |
| S11 | (running or skating or skiing or Jog* or zumba or kettlebelt or Swim* or Bicycl* or cycling or bicycling or fitness bike* or exercise bike* or spinning or walk* or rowing or rower or water-rower* or waterrower* or cross-train* or crosstrain* or pacing or paced or titreted or soccer or football or handball or cricket or hockey or baseball or basketball or volleyball or ball game* or (play* N2 ball) or (weight* N3 (lift* or train* or exercis*))) |  | 148,900 |
| S10 | (aerobic* or anaerobic* or movement therapy or watergym* or wateraerobic* or water-gym* or water-aerobic* or aquagym or ((water or aqva or aqua or pool) N1 (aerobic* or gym*)) or gym or gymnastic* or hydrotherap* or ((warm water or aqvatic or aquatic) N4 (exercis* or train* or rehab*))) |  | 38,030 |
| S9 | TI rehabilitation OR SU rehabilitation |  | 157,016 |
| S8 | ((muscl* or neuromuscul* or neuro-muscul* or resistance or physical or kinesitherap* or kinesiotherap* or endurance or strength* or balance or postur* or gait or threshold* or treadmill* or aquatic* or trunk or graded or gradual or progressive or controlled or incremented or symptom-based or symptombased or symptom based or guided or supervis* or coached or coaching or stepped care or stepwise or step-wise) N3 (train* or retrain* or re-train* or exercise* or strength* or fitness or fit or condition* or exposure or activit* or performance or exertion or load*)) |  | 540,294 |
| S7 | ((exercise or movement or physical therapy or physiotherapy or rehabilitative) N3 (training or intervention or technique* or program* or strateg* or approach or session* or class* or isometric)) |  | 156,376 |
| S6 | TI physical N1 (education or program*) OR SU physical N1 (education or program*) |  | 10,662 |
| S5 | TI (exercis* or train* or stepped care or physical therap* or physiotherap* or kinesiotherapy) |  | 147,458 |
| S4 | MH "Physical therapy" or MH "Exercise+" or MH "Exercise therapy+" or MH Sports+ or MH "Physical Exertion+" or MH "Muscle strength+" or MH "Home rehabilitation+" or MH "Rehabilitation, Community-Based" or MH "Exercise Movement Techniques+" or MH "physical therapy modalities+ or MH "Physical Education and Training+" or MH "Weight-Bearing" |  | 234,549 |
| S3 | S1 OR S2 |  | 45,117 |
| S2 | ((prostatic or prostate or ((male* or men) and (urogenital or genital))) and (cancer* or neoplas* or tumo#r* or carcinoma* or oncolog* or postcancer or post-cancer)) or (androgen deprivation therapy or prostatect* or post-prostatect* or castration) |  | 45,117 |
| S1 | MH "Prostatic Neoplasms+" or MH Prostatectomy |  | 33,133 |

**PEDRO**

61 hits

Abstract & Title: Prostate cancer OR Prostatic neoplasm*

Method: Clinical Trial

Published since: 2015

**Updated search for randomized controlled studies/controlled studies 2020-2021**

Search date: 16.06.2021

**Medline**

Database(s): Ovid MEDLINE(R) and Epub Ahead of Print, In-Process, In-Data-Review & Other Non-Indexed Citations, Daily and Versions(R) 1946 to June 15, 2021 
Search Strategy:

| # | Searches | Results |
| --- | --- | --- |
| 1 | exp Prostatic neoplasm/ | 133895 |
| 2 | ((prostatic or prostate or ((male* or men) and (urogenital or genital))) and (cancer* or neoplas* or tumo?r* or carcinoma* or oncolog* or postcancer or post-cancer)).ti,bt,ab,kf. | 170365 |
| 3 | Prostatectomy/ | 29649 |
| 4 | (androgen deprivation therapy or prostatect* or post-prostatect* or castration).ti,bt,ab,kf. | 57604 |
| 5 | or/1-4 | 214594 |
| 6 | exp exercise/ | 210950 |
| 7 | exp exercise therapy/ | 54877 |
| 8 | exp Sports/ | 192987 |
| 9 | exp Physical Exertion/ | 56842 |
| 10 | exp Muscle strength/ | 37162 |
| 11 | exp Exercise Movement Techniques/ | 8852 |
| 12 | exp "Physical Education and Training"/ | 13729 |
| 13 | physical therapy modalities/ | 37970 |
| 14 | Weight-Bearing/ | 20917 |
| 15 | (exercis* or train* or stepped care or physical therap* or physiotherap* or kinesiotherapy).ti,bt,kf. | 311593 |
| 16 | (physical adj1 (education or program*)).ti,bt,ab,kf. | 6321 |
| 17 | ((exercise or movement) adj3 (session* or training or technique* or physical or isometric or therap* or program* or class*)).ti,bt,ab,kf. | 74885 |
| 18 | ((physical therapy or physiotherapy) adj3 (exercise* or session* or training or technique* or isometric or program* or class*)).ti,bt,ab,kf. | 5007 |
| 19 | ((muscl* or neuromuscul* or neuro-muscul* or resistance or physical or kinesiotherap* or kinesitherap* or endurance or strength* or balance or postur* or gait or threshold* or treadmill* or aquatic* or trunk or graded or gradual or progressive or controlled or incremented or symptom-based or symptombased or symptom based or guided or supervis* or coached or coaching or stepped care or stepwise or step-wise) adj3 (train* or retrain* or re-train* or exercise* or rehabilitation or strength* or fitness or fit or condition* or exposure or activit* or performance or exertion or load*)).ti,bt,ab,kf. | 784440 |
| 20 | (active adj1 rehabilitation).ti,bt,ab,kf. | 482 |
| 21 | (aerobic* or anaerobic* or movement therapy or pilates or watergym* or wateraerobic* or water-gym* or water-aerobic* or aquagym or ((water or aqva or aqua or pool) adj1 (aerobic* or gym*)) or gym or gymnastic* or ((warm water or aqvatic or aquatic) adj4 (exercis* or train* or rehab*))).ti,bt,ab,kf. | 159747 |
| 22 | (running or skating or skiing or Jog* or zumba or kettlebelt or Swim* or Bicycl* or cycling or bicycling or fitness bike* or exercise bike* or spinning or walking or rowing or rower or water-rower* or waterrower* or cross-train* or crosstrain* or pacing or paced or titreted or soccer or football or handball or cricket or hockey or baseball or basketball or volleyball or ball game* or (play* adj2 ball) or (weight* adj3 (lift* or train* or exercis*))).ti,bt,ab,kf. | 357911 |
| 23 | ((upper body or lower body) adj2 (exercise* or training or program*)).ti,bt,ab,kf. | 915 |
| 24 | ((mckenzie or mechanical or Alexander or William or Feldenkrais) adj2 (technique* or model* or tool* or exercise* or therapy)).ti,bt,ab,kf. | 7717 |
| 25 | ((non-pharma* or nonpharma* or non pharma* or non-drug or nondrug or non drug or non-surgical or nonsurgical or non surgical or non-surgery or nonsurgery or non surgery or non-invasive or noninvasive or non invasive or complimentary or integrat* or holistic or multi-modal* or multimodal* or Multidisciplinary or Multi-disciplinary or Interdisciplinary or Inter-disciplinary or complex or multimodal* or multi-modal* or cross-disciplinary or crossdisciplinary or multi-dimensional or multidimensional or multi-facet* or multifacet* or comprehensive or multiple or rehabilitati*) adj6 (therap* or treat* or intervention* or action* or program* or strateg* or protocol* or support* or approach* or evaluation or rehabilitation or care)).ti,bt,kf. | 197531 |
| 26 | or/6-25 | [1726217](tel:1726217) |
| 27 | 5 and 26 | 6008 |
| 28 | limit 27 to (randomized controlled trial or controlled clinical trial) | 396 |
| 29 | (((random* or cluster-random* or quasi-random* or control?ed or crossover or cross-over or blind* or mask*) adj4 (trial*1 or study or studies or analy*)) or rct).ti,bt,ab,kf,hw. | [1127384](tel:1127384) |
| 30 | (placebo* or single-blind* or double-blind* or triple-blind*).ti,bt,kf,hw. | 234391 |
| 31 | ((single or double or triple) adj2 (blind* or mask*)).ti,bt,kf,hw. | 204885 |
| 32 | ((patient* or person* or participant* or population* or allocat* or assign*) adj3 random*).ti,bt,ab,kf. | 265990 |
| 33 | or/29-32 | [1217233](tel:1217233) |
| 34 | 27 and 33 | 900 |
| 35 | 28 or 34 | 900 |
| 36 | limit 35 to (yr="2020-2021" and (english or danish or norwegian or swedish)) | 131 |

**Embase**

Database(s): Embase 1974 to 2021 June 15 
Search Strategy:

| # | Searches | Results |
| --- | --- | --- |
| 1 | exp Prostate cancer/ or exp Prostate tumor/ | 256519 |
| 2 | androgen deprivation therapy/ | 13641 |
| 3 | Prostatectomy/ | 58915 |
| 4 | ((prostatic or prostate or ((male* or men) and (urogenital or genital))) and (cancer* or neoplas* or tumo?r* or carcinoma* or oncolog* or postcancer or post-cancer)).ti,ab,kw. | 253103 |
| 5 | (androgen deprivation therapy or prostatect* or post-prostatect* or castration).ti,ab,kw. | 86214 |
| 6 | or/1-5 | 330912 |
| 7 | exp exercise/ | 364888 |
| 8 | exp kinesiotherapy/ | 84331 |
| 9 | physiotherapy/ | 89944 |
| 10 | home physiotherapy/ | 354 |
| 11 | physical activity/ or cycling/ or jogging/ or jumping/ or lifting effort/ or running/ or scratching/ or stretching/ or swimming/ or exp walking/ or weight bearing/ or weight lifting/ | 392750 |
| 12 | exp Muscle strength/ | 68781 |
| 13 | exp Physical education/ | 11778 |
| 14 | (exercis* or train* or stepped care or physical therap* or physiotherap* or kinesiotherapy).ti,kw. | 396026 |
| 15 | (physical adj1 (education or program*)).ti,ab,kw. | 7660 |
| 16 | ((exercise or movement) adj3 (session* or training or technique* or physical or isometric or therap* or program* or class*)).ti,ab,kw. | 102760 |
| 17 | ((physical therapy or physiotherapy) adj3 (exercise* or session* or training or technique* or isometric or program* or class*)).ti,ab,kw. | 8746 |
| 18 | ((muscl* or neuromuscul* or neuro-muscul* or resistance or physical or kinesiotherap* or kinesitherap* or endurance or strength* or balance or postur* or gait or threshold* or treadmill* or aquatic* or trunk or graded or gradual or progressive or controlled or incremented or symptom-based or symptombased or symptom based or guided or supervis* or coached or coaching or stepped care or stepwise or step-wise) adj3 (train* or retrain* or re-train* or exercise* or rehabilitation or strength* or fitness or fit or condition* or exposure or activit* or performance or exertion or load*)).ti,ab,kw. | 966593 |
| 19 | (active adj1 rehabilitation).ti,ab,kw. | 715 |
| 20 | (aerobic* or anaerobic* or movement therapy or pilates or watergym* or wateraerobic* or water-gym* or water-aerobic* or aquagym or ((water or aqva or aqua or pool) adj1 (aerobic* or gym*)) or gym or gymnastic* or ((warm water or aqvatic or aquatic) adj4 (exercis* or train* or rehab*))).ti,ab,kw. | 193112 |
| 21 | (running or skating or skiing or Jog* or zumba or kettlebelt or Swim* or Bicycl* or cycling or bicycling or fitness bike* or exercise bike* or spinning or walking or rowing or rower or water-rower* or waterrower* or cross-train* or crosstrain* or pacing or paced or titreted or soccer or football or handball or cricket or hockey or baseball or basketball or volleyball or ball game* or (play* adj2 ball) or (weight* adj3 (lift* or train* or exercis*))).ti,ab,kw. | 452668 |
| 22 | ((upper body or lower body) adj2 (exercise* or training or program*)).ti,ab,kw. | 1038 |
| 23 | ((mckenzie or mechanical or Alexander or William or Feldenkrais) adj2 (technique* or model* or tool* or exercise* or therapy)).ti,ab,kw. | 8983 |
| 24 | ((non-pharma* or nonpharma* or non pharma* or non-drug or nondrug or non drug or non-surgical or nonsurgical or non surgical or non-surgery or nonsurgery or non surgery or non-invasive or noninvasive or non invasive or complimentary or integrat* or holistic or multi-modal* or multimodal* or Multidisciplinary or Multi-disciplinary or Interdisciplinary or Inter-disciplinary or complex or multimodal* or multi-modal* or cross-disciplinary or crossdisciplinary or multi-dimensional or multidimensional or multi-facet* or multifacet* or comprehensive or multiple or rehabilitati*) adj6 (therap* or treat* or intervention* or action* or program* or strateg* or protocol* or support* or approach* or evaluation or rehabilitation or care)).ti,kw. | 272820 |
| 25 | or/7-24 | 2251567 |
| 26 | 6 and 25 | 12242 |
| 27 | limit 26 to (randomized controlled trial or controlled clinical trial) | 842 |
| 28 | (((random* or cluster-random* or quasi-random* or control?ed or crossover or cross-over or blind* or mask*) adj4 (trial*1 or study or studies or analy*)) or rct).ti,ab,kw. | [1054899](tel:1054899) |
| 29 | (placebo* or single-blind* or double-blind* or triple-blind*).ti,ab. | 422454 |
| 30 | ((single or double or triple) adj2 (blind* or mask*)).ti,ab. | 253412 |
| 31 | ((patient* or person* or participant* or population* or allocat* or assign*) adj3 random*).ti,ab. | 370614 |
| 32 | or/28-31 | [1339457](tel:1339457) |
| 33 | 26 and 32 | 1526 |
| 34 | 27 or 33 | 1730 |
| 35 | limit 34 to (yr="2020-2021" and (english or danish or norwegian or swedish)) | 189 |

**Cinahl**

| AM |
| --- |

| # | Query | Limiters/Expanders | Results |
| --- | --- | --- | --- |
| S21 | S15 AND S20 | Limiters - Published Date: [20200601-20210631](tel:20200601-20210631); Language: Danish, English, Norwegian, Swedish | 19 |
| S20 | S16 OR S17 OR S18 OR S19 |  | 562,238 |
| S19 | ((patient* or person* or participant* or population* or allocat* or assign*) N3 (random* or blind* or mask*)) |  | 366,741 |
| S18 | (placebo* or single-blind* or double-blind* or triple-blind* or ((single or double or triple) N1 (blind* or mask*)) |  | 116,180 |
| S17 | (((random* or cluster-random* or quasi-random* or control#ed or crossover or cross-over or blind* or mask*) N4 (trial* or study or studies or analy*)) or rct) |  | 533,733 |
| S16 | PT Randomized Controlled Trial OR MH "Randomized Controlled Trial+" OR PT "Controlled Clinical Trial" OR MH "Controlled Clinical Trial+" |  | 128,727 |
| S15 | S3 AND S14 |  | 3,900 |
| S14 | S4 OR S5 OR S6 OR S7 OR S8 OR S9 OR S10 OR S11 OR S12 OR S13 |  | 998,717 |
| S13 | TI ((non-surgical or nonsurgical or non surgical or non-surgery or nonsurgery or non surgery or non-invasive or noninvasive or non invasive or complimentary or integrat* or holistic or multi-modal* or multimodal* or Multidisciplinary or Multi-disciplinary or Interdisciplinary or Inter-disciplinary or complex or multimodal* or multi-modal* or cross-disciplinary or crossdisciplinary or multi-dimensional or multidimensional or multi-facet* or multifacet* or comprehensive or multiple) N3 (therap* or treat* or intervention* or action* or program* or strateg* or protocol* or support* or approach* or evaluation or care)) OR SU ((non-surgical or nonsurgical or non surgical or non-surgery or nonsurgery or non surgery or non-invasive or noninvasive or non invasive or complimentary or integrat* or holistic or multi-modal* or multimodal* or Multidisciplinary or Multi-disciplinary or Interdisciplinary or Inter-disciplinary or complex or multimodal* or multi-modal* or cross-disciplinary or crossdisciplinary or multi-dimensional or multidimensional or multi-facet* or multifacet* or comprehensive or multiple) N3 (therap* or treat* or intervention* or action* or program* or strateg* or protocol* or support* or approach* or evaluation or care)) |  | 104,977 |
| S12 | ((upper body or lower body) N2 (exercise* or training or program*)) |  | 585 |
| S11 | (running or skating or skiing or Jog* or zumba or kettlebelt or Swim* or Bicycl* or cycling or bicycling or fitness bike* or exercise bike* or spinning or walk* or rowing or rower or water-rower* or waterrower* or cross-train* or crosstrain* or pacing or paced or titreted or soccer or football or handball or cricket or hockey or baseball or basketball or volleyball or ball game* or (play* N2 ball) or (weight* N3 (lift* or train* or exercis*))) |  | 165,246 |
| S10 | (aerobic* or anaerobic* or movement therapy or watergym* or wateraerobic* or water-gym* or water-aerobic* or aquagym or ((water or aqva or aqua or pool) N1 (aerobic* or gym*)) or gym or gymnastic* or hydrotherap* or ((warm water or aqvatic or aquatic) N4 (exercis* or train* or rehab*))) |  | 37,327 |
| S9 | TI rehabilitation OR SU rehabilitation |  | 153,126 |
| S8 | ((muscl* or neuromuscul* or neuro-muscul* or resistance or physical or kinesitherap* or kinesiotherap* or endurance or strength* or balance or postur* or gait or threshold* or treadmill* or aquatic* or trunk or graded or gradual or progressive or controlled or incremented or symptom-based or symptombased or symptom based or guided or supervis* or coached or coaching or stepped care or stepwise or step-wise) N3 (train* or retrain* or re-train* or exercise* or strength* or fitness or fit or condition* or exposure or activit* or performance or exertion or load*)) |  | 534,102 |
| S7 | ((exercise or movement or physical therapy or physiotherapy or rehabilitative) N3 (training or intervention or technique* or program* or strateg* or approach or session* or class* or isometric)) |  | 153,546 |
| S6 | TI physical N1 (education or program*) OR SU physical N1 (education or program*) |  | 10,523 |
| S5 | TI (exercis* or train* or stepped care or physical therap* or physiotherap* or kinesiotherapy) |  | 146,715 |
| S4 | MH "Physical therapy" or MH "Exercise+" or MH "Exercise therapy+" or MH Sports+ or MH "Physical Exertion+" or MH "Muscle strength+" or MH "Home rehabilitation+" or MH "Rehabilitation, Community-Based" or MH "Exercise Movement Techniques+" or MH "physical therapy modalities+ or MH "Physical Education and Training+" or MH "Weight-Bearing" |  | 230,153 |
| S3 | S1 OR S2 |  | 46,252 |
| S2 | ((prostatic or prostate or ((male* or men) and (urogenital or genital))) and (cancer* or neoplas* or tumo#r* or carcinoma* or oncolog* or postcancer or post-cancer)) or (androgen deprivation therapy or prostatect* or post-prostatect* or castration) |  | 46,252 |
| S1 | MH "Prostatic Neoplasms+" or MH Prostatectomy |  | 33,309 |

**PEDRO**

16 hits

Abstract & Title: Prostate cancer OR Prostatic neoplasm*

Method: Clinical Trial

Published since: 2020
